# Synthesis of 1,3-Dihydro-2,1-benzisoxazoles by Intramolecular C–N Coupling: Evaluation as an Indane Bioisostere

**DOI:** 10.1021/acsmedchemlett.6c00429

**Published:** 2026-09-27

**Authors:** Thomas D. Beckler, David Crich

**Affiliations:** † Department of Chemistry, 1355 University of Georgia, Athens, Georgia 30602, United States; ‡ Innovations in Drug Discovery Program, Department of Pharmaceutical and Biomedical Sciences, University of Georgia, Athens, Georgia 30602, United States; § Complex Carbohydrate Research Center, University of Georgia, Athens, Georgia 30602, United States

**Keywords:** Hydroxylamine, Benzisoxazole, Bioisostere, MT1, MT2, Indane

## Abstract

Despite the ever-increasing interest in isoxazole-based compounds, benzo-fused isoxazolidines, or 1,3-dihydro-2,1-benzisoxazoles, are very rare. Seeking to fill this gap, we present a method for the synthesis of 1,3-dihydro-2,1-benzisoxazoles by intramolecular Cu-mediated or Pd-catalyzed C–N coupling of bromoaryl benzylic hydroxylamine derivatives, which can be further derivatized through a simple reductive amination sequence. This approach overcomes the limitations of previous methods and provides access to multiple novel heterocyclic scaffolds. As a proof of concept, we apply the method to the synthesis of a benzisoxazoline analogue of the metabolically labile chiral indane-based MT1/MT2 receptor agonist ramelteon, ultimately furnishing a compound with a >10-fold improvement in metabolic stability in human hepatocytes. Support for the bioisosteric nature of the 1,3-dihydro-2,1-benzisoxazole and indane cores was provided in the context of the ramelteon analogue by DFT calculations.

## Graphical abstract

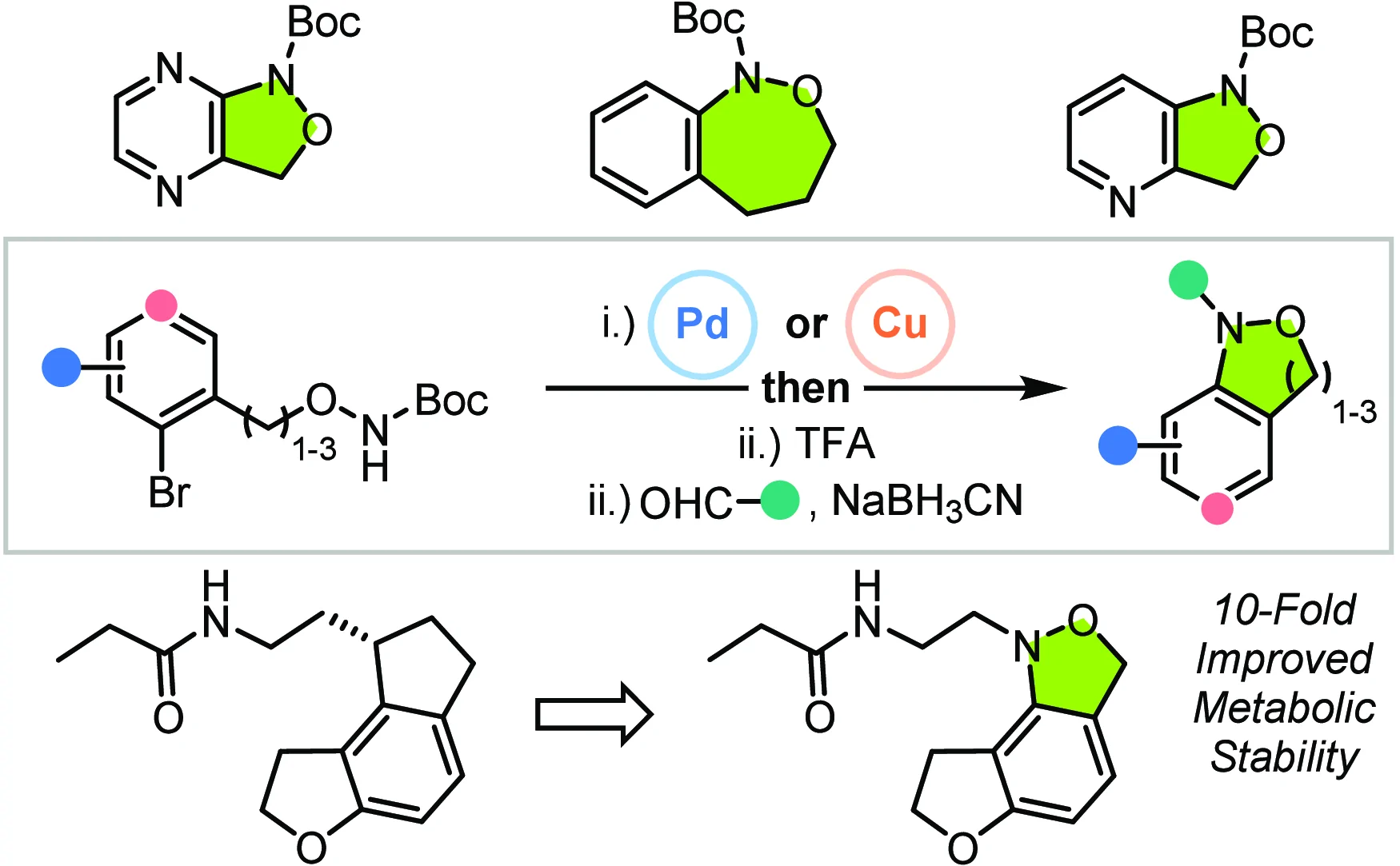

Nitrogen- and oxygen-containing heterocycles are ubiquitous motifs in medicinal chemistry and are present in a large proportion (>50%^1^ and 25%,^2^ respectively) of all small-molecule drugs. The ever-increasing proportion of nitrogen-heterocycle-containing drugs,^3^ coupled with the increasing complexity of synthetic targets and constant mutation of small-molecule binding sites, provides strong impetus for the design of novel heterocyclic frameworks. Featuring both nitrogen and oxygen, the isoxazole class of heterocycle and its reduced congeners, the isoxazolines and isoxazolidines, are widely regarded as privileged scaffolds in organic and medicinal chemistry^4–8^ and can be found in numerous natural products, including zeteketoxin AB,^9^ subereamollines A and B,^10^ and aerothionin,^11^ and in many pharmaceuticals, such as the WHO essential antibiotic cloxacillin,^7^ the GP inhibitor roxifiban,^7^ and the clinical candidate EGFR inhibitor enozertinib^12^ (Figure 1a). Moreover, interest in this family of heterocycles continues to grow, as revealed by a database search,^13–15^ which showed an exponential increase in reported isoxazole-based compounds since 2000 as well as the increasing number of isoxazole­(idine)-containing FDA-approved drugs.^16^ Given this burgeoning interest in isoxazoles and their reduced forms, it is surprising that very few members of the benzo-fused isoxazolidines, or 1,3-dihydro-2,1-benzisoxazoles, and an equally limited range of methods to access them, have been described^17–23^ thereby providing a rare opportunity for *de novo* heterocycle synthesis (Figure 1b).

**Figure 1:**
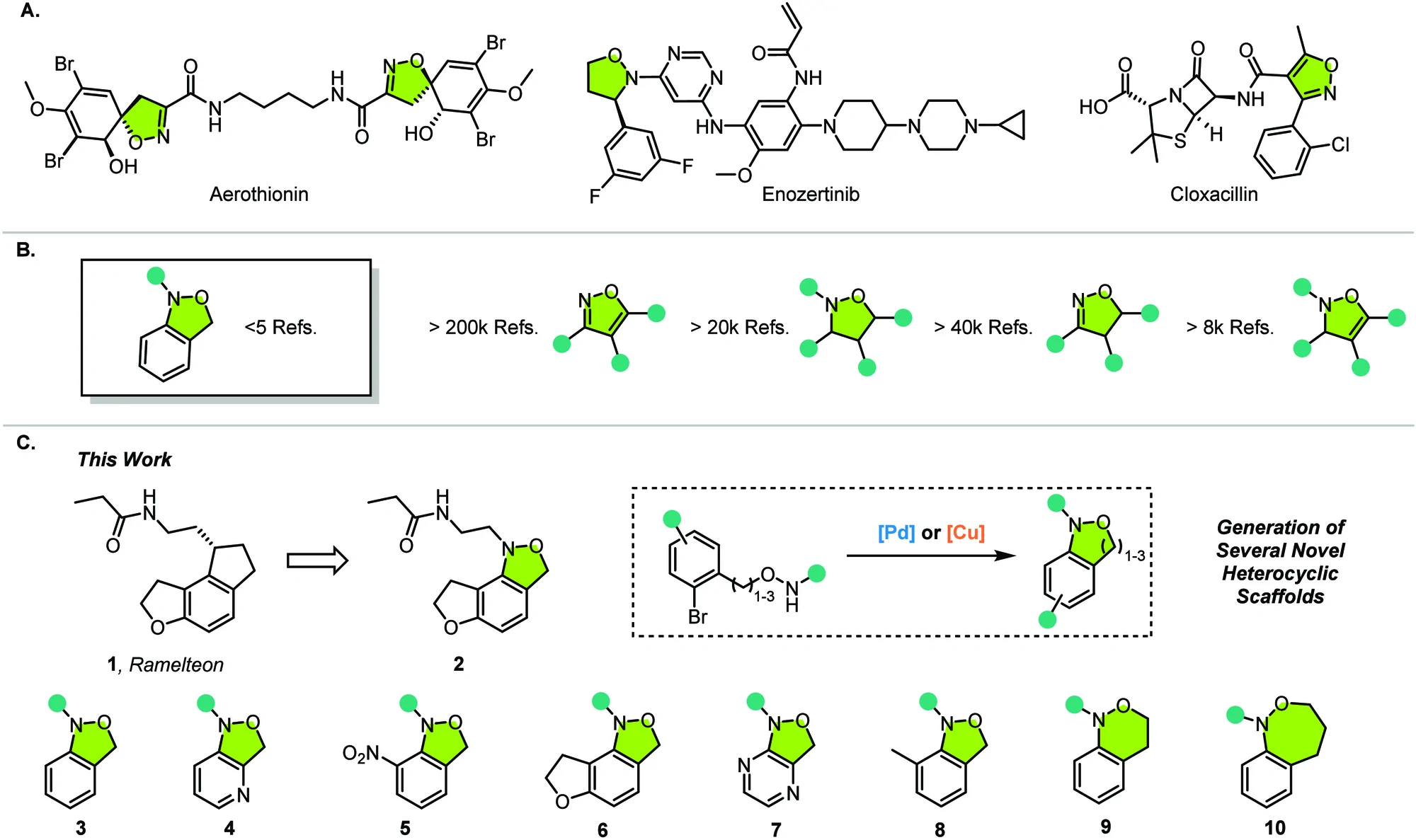
(a) Drugs and natural products containing an isoxazole derivative. (b) Current reference counts of isoxazole derivatives as reflected by a Reaxys substructure search (April 2026), depicting the relative scarcity of the 1,3-dihydro-2,1-benzisoxazole core.^13–15^ (c) Preparation of hydroxalog **2** and heterocycles **3**–**10** via intramolecular C–N bond formation.

The 1,3-dihydro-2,1-benzisoxazole core embodies a trisubstituted hydroxylamine moiety, a functional group for which we have been developing synthetic methods^17,22,24–26^ so as to enable their use as bioisosteres for a range of structural features.^27–32^ Attractive features of the trisubstituted hydroxylamines, or hydroxalogs, include the low p*K*_a_ (∼5.9) of the conjugate acid^33,34^ and a low barrier to stereomutation by pyramidal inversion at nitrogen coupled with rotation about the N–O bond (∼15 kcal mol^–1^),^35,36^ enabling their use as an adaptable achiral replacement for stereogenic centers.^27,37,38^ Additionally, hydroxalogs offer the possibly of prolonged half-lives when used as isosteric replacements of metabolic hotspots such as benzylic C–H bonds; finally, despite common perceptions to the contrary, hydroxalogs are not inherently mutagenic or genotoxic.^27–32^

Accordingly, we embarked on a program targeting the synthesis and medicinal chemistry of 1,3-dihydro-2,1-benzisoxazoles. Our initial approaches to this scaffold relied upon the reduction of more readily accessible benzisoxazolones^17,18^ or intramolecular N–O bond formation.^22^ While these methods were successful, they suffered from either a narrow substrate scope or the reliance on the preparation and use of peroxide-based electrophiles. Seeking to develop a more broadly applicable method and inspired by the widespread application of Pd- and Cu-catalyzed C–N bond-forming reactions in pharmaceutical development and production,^39,40^ we set out to develop methods for the synthesis of 1,3-dihydro-2,1-benzisoxazoles by intramolecular C–N cross-coupling through Pd-catalyzed Buchwald–Hartwig or Cu-mediated Ullman–Goldberg-type couplings and report here on the reduction to practice of these strategies. To illustrate the potential of the new methodology, we selected the picomolar MT1/MT2 dual receptor agonist ramelteon,^41–43^ replacing its indane subunit, with its known benzylic C–H bond metabolic hotspot,^44^ by the achiral 1,3-dihydro-2,1-benzisoxazole nucleus (Figure 1c), thereby achieving an improved half-life in human hepatocytes, with additional support for the bioisosteric replacement provided by a docking study and DFT-based conformational analysis.

The Wuest^45^ and Tomkinson^46,47^ laboratories have demonstrated that *N*-acyl-*N*,*O*-disubstituted hydroxylamines are competent nucleophiles in intermolecular C–N cross-couplings with aryl halides under Pd or Cu catalysis, while the Lebel group showed that *N*-aryl hydroxylamines can be prepared by intermolecular Cu-mediated coupling with a hypervalent iodine–hydroxylamine reagent.^48^ Extrapolating from these intermolecular reactions and the many syntheses of indolines by Pd- or Cu-catalyzed cyclization of *o*-(aminoethyl)­aryl halides,^49–52^ we hypothesized that 1,3-dihydro-2,1-benzisoxazoles could be accessed by intramolecular transition metal-mediated coupling of haloaryl benzylic hydroxylamine derivatives. To test this proposal, we synthesized benzylic hydroxylamine **17** by *O*-alkylation of *N*-Boc-hydroxylamine with 2-bromobenzyl bromide.^53^ Gratifyingly, subjecting **17** to ring closure with the Pd SPhos G3 precatalyst provided the desired benzisoxazole **18** in 57% yield (Scheme 1). Given the incompatibility of our previous protocol^17^ with substitution in the arene ring ortho to the isoxazole nitrogen, we next synthesized the *o*-methyl derivative **19** and were pleased to find that Pd-catalyzed cyclization afforded the product **20** smoothly in 81% yield (Scheme 1). Further extrapolation of the method to the heteroatom-containing substrate benzofuran **21** afforded the desired 1,3-dihydro-2,1-benzisoxazole **22** but only in 23% yield with significant recovery of the starting material (Scheme 1). Attempts to optimize this cyclization by employing different ligand and catalyst systems resulted only in diminished yields (Figure S1). As Cu-based systems are widely used in intramolecular couplings to form indolines,^51,52^ we turned to Cu-mediated coupling, which led to a slightly improved yield of 30% but with reduced reaction time, fewer side products, and full consumption of the starting materials. Further tuning of the reaction conditions led to a significantly improved yield of 62% under operationally simple conditions (Figure S2). To our knowledge, this method is the first report of an intramolecular Cu-mediated aryl C–N coupling of a hydroxylamine derivative, while the Pd-based method is only the second example of its class^21^ and the first without the need for directing groups.

**Scheme 1:**
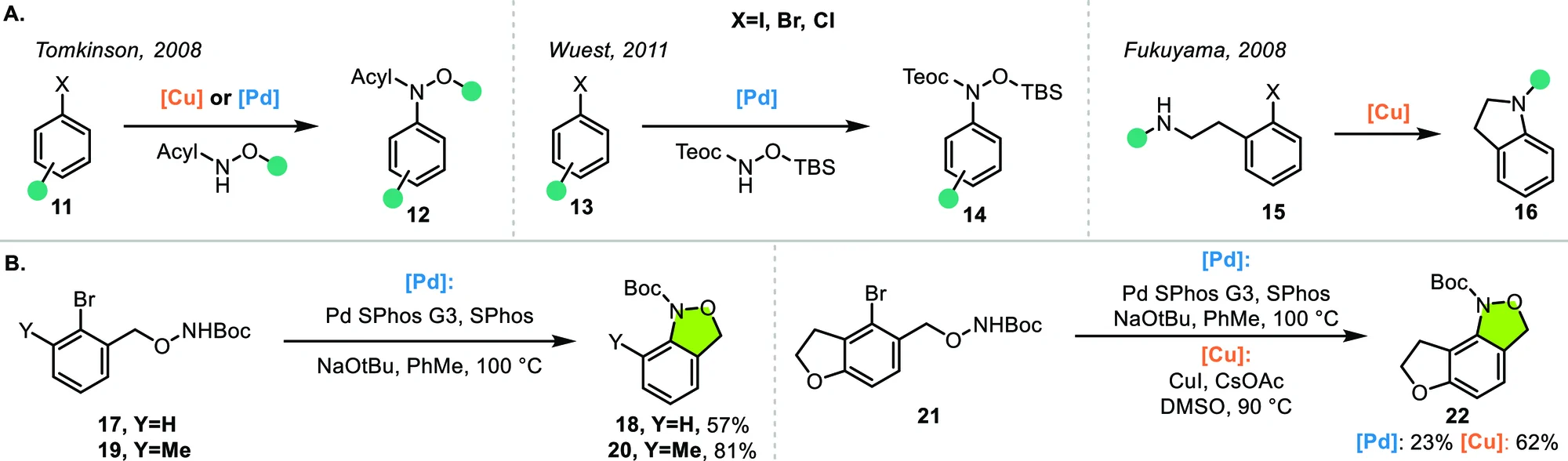
(A) Precedent for Intermolecular Arylation of *N*-Acyl Hydroxylamines and for Intramolecular C–N Bond Formation; (B) Initial Demonstrations of the Intramolecular Pd- and Cu-Mediated Arylation of Hydroxylamines

We briefly explored the scope of these methods focusing on substrates with ortho substituents, heteroarene scaffolds, and expanded ring sizes, none of which were accessible by our previous methods (Table 1). Thus, we targeted the core scaffolds **18**–**29** and applied both the Pd and Cu cross-coupling methods. The precursors were obtained commercially as the corresponding benzyl bromides or methylarene derivatives, with the latter being first subjected to radical bromination and then alkylation with *N*-Boc-hydroxylamine (General Procedures A and B in the Supporting Information (SI)). Cyclization to give the sterically encumbered *o*-nitroarene **23** derivative proceeded smoothly under both conditions, albeit with reduced yields, demonstrating the protocol’s tolerance of ortho substitution. We next turned to heterocyclic and heteroarene scaffolds, beginning with the fused pyridine and pyrazine derivatives **24** and **25**, both of which could be prepared in fair to good yields by application of the Cu-mediated method but failed in the Pd-based sequence. Extension of these protocols to the synthesis of expanded six- and seven-membered rings was also successful, furnishing **26** and **27** by both the Pd and Cu methods. The limitations of the method were revealed by the failure to access pyrimidine **28** and the formation of only trace amounts of thiophene **29**. In general, while the Cu- and Pd-catalyzed methods performed similarly for simple arene scaffolds, the Cu-based approach functioned better for heteroatom-containing systems. Interestingly, several examples, primarily when using the Cu method, produced the corresponding 1,2-amino aldehyde as a byproduct, which likely arises from oxidative addition of the metal species into the N–O bond^54–56^ and subsequent β-hydride elimination with concomitant N–O cleavage. This suggests an interesting potential synthetic application of these compounds as latent 1,2-amino aldehyde precursors. In any case, the resultant aldehyde byproduct could be easily removed by column chromatography or sodium bisulfite workup.^57,58^ Overall, these methods successfully overcame the shortcomings of our previous methodologies and provided access to novel heterocycles such as **24**, **25**, and **27**.

**Scheme 2:**
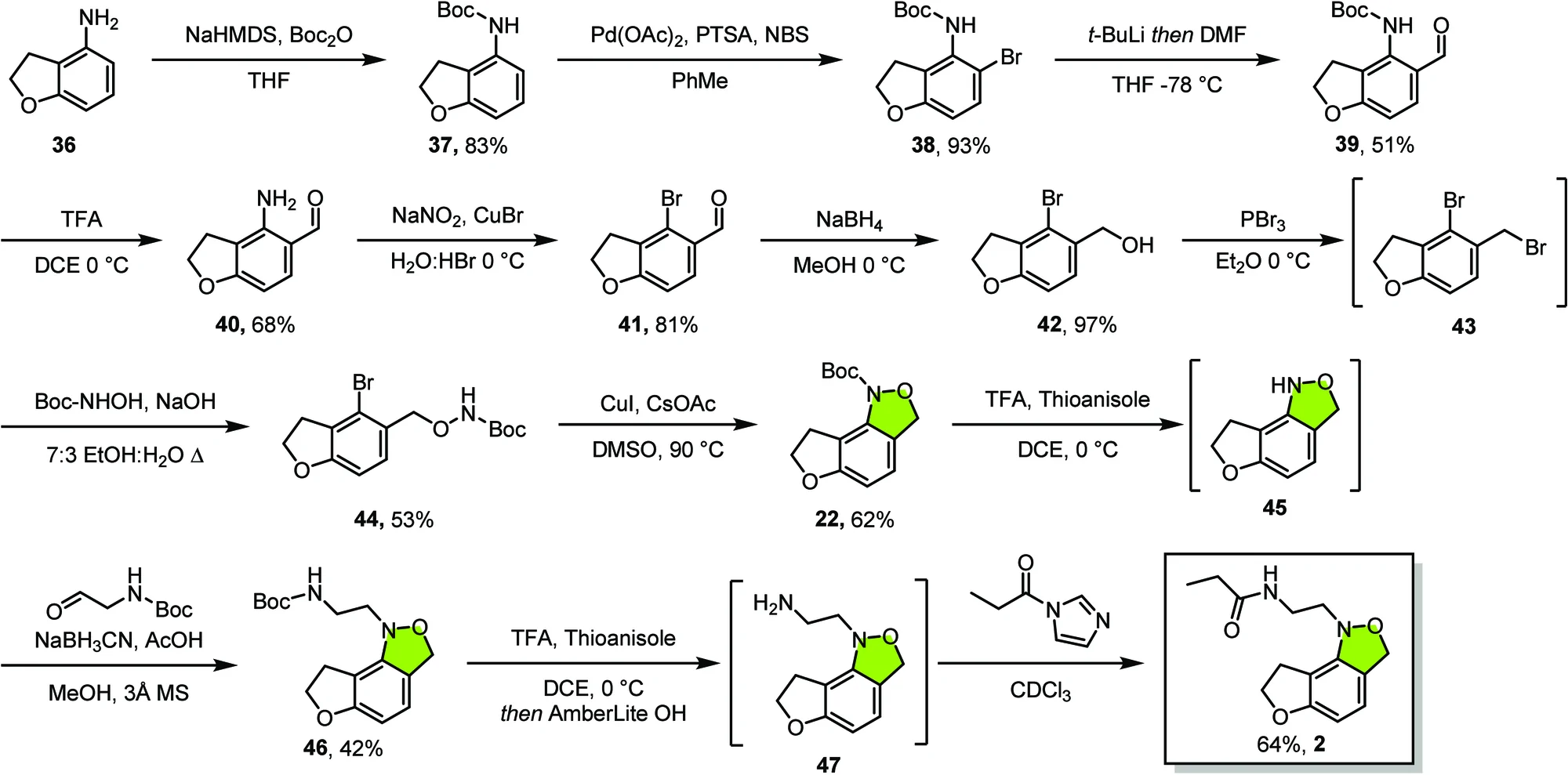
Synthesis of Hydroxalog **2** Starting from Benzofuranamine **36**

**Table 1:**
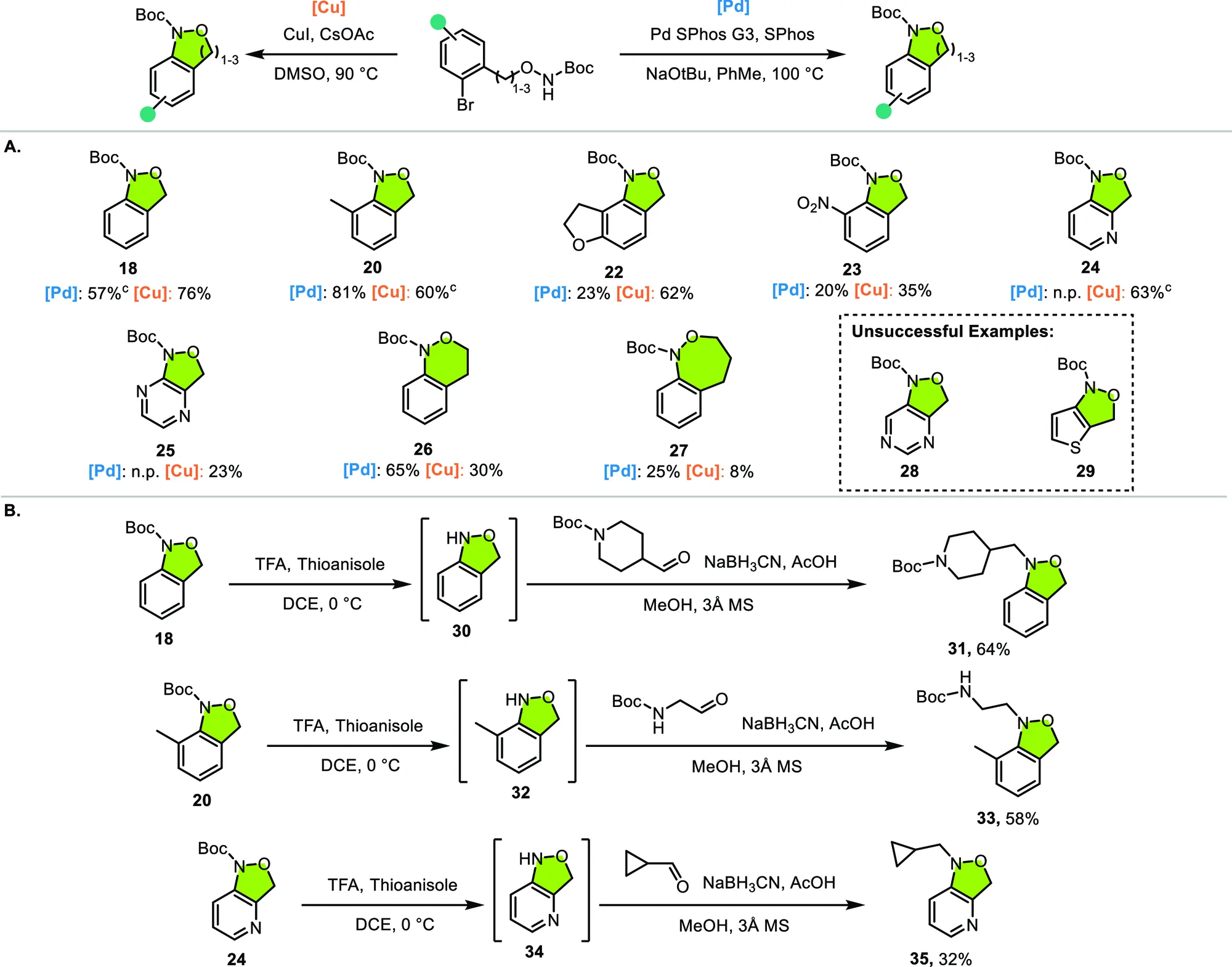
Scope of Pd and Cu Cross Hydroxylamine Couplings and Subsequent Derivatization through Reductive Amination^a^ ^,^ ^b^

a Yields are reported after isolation.

b All reactions were performed on a 0.05–0.3 mmol scale, unless stated otherwise.

c The reaction was conducted on a >1 mmol scale.

To further elaborate on the new cores, we functionalized **18**, **20**, and **24** at nitrogen through a simple deprotection and reductive amination sequence. Beginning with **18**, Boc removal with TFA in the presence of thioanisole furnished the corresponding secondary hydroxylamine, which was subjected to reductive amination with *N*-Boc-4-formyl-piperidine, providing the drug-like scaffold **31** in 64% yield over two steps. Similarly, *o*-methyl species **20** was alkylated with *N*-Boc-2-aminoacetaldehyde to furnish **33** in 58% overall yield. Finally, the fused pyridine **24** was subjected to the same conditions in the presence of cyclopropanecarboxaldehyde to afford **35** in 32% over two steps.

To evaluate the 1,3-dihydro-2,1-benzisoxazole core in a biologically active scaffold, we turned to the ramelteon hydroxalog **2**, starting from commercially available 2,3-dihydrobenzofuranamine **36** (Scheme 2). The amine was first Boc-protected to produce **37**, which underwent directed *ortho*-selective C–H bromination^59^ to furnish bromide **38** in 93% yield. The use of NaHMDS as the base proved to be critical in order to obtain a high yield of the mono-Boc-protected amine while minimizing side product formation.^60^ Compound **38** was then subjected to *t*-BuLi-mediated lithium–halogen exchange and subsequent trapping with DMF to furnish aldehyde **39** in 51% yield. Boc removal with TFA then yielded aniline **40**. Sandmeyer bromination^61^ gave aryl bromide **41** in 81% yield. Borohydride reduction of **41** provided benzyl alcohol **42** in excellent yield without the need for additional purification, and subsequent bromination with PBr_3_ furnished the corresponding benzyl bromide **43**. While **43** could be isolated by silica gel column chromatography, higher overall yields were obtained by immediately subjecting it to alkylation with *N*-Boc-hydroxylamine to furnish *O*-benzyl hydroxylamine **44** in 53% over two steps.

Compound **44** was then subjected to the Cu-mediated cyclization protocol, furnishing 1,3-dihydro-2,1-benzisoxazole **22** in 62% yield. The Boc group was removed to give **45** in the standard manner, and the resultant hydroxylamine was subjected to reductive amination with *N*-Boc-2-aminoacetaldehyde to afford amine **46** in 42% yield over two steps. Boc removal from **46** with TFA cleanly afforded the corresponding primary amine as its TFA salt, and after liberation of the free base using AmberLite, installation of the amide was achieved by treatment of **47** with propionyl imidazolide, furnishing the target hydroxalog **2**.

As expected, introduction of the trisubstituted hydroxylamine in **2** resulted in a decrease in logP and logD by ∼1 unit (Figure 2) with respect to the parent ramelteon **1**. The aqueous solubility of **2** remained high, and the membrane permeability was virtually unchanged in both MDCKII-MDR1 and Caco-2 assays, with efflux ratios of 0.78 and 0.76 for **1** and 0.86 and 0.72 for **2**, respectively (Figure 2). Gratifyingly, in human hepatocytes there was a 10-fold reduction in clearance, resulting in a 10-fold increase in *t*_1/2_ of hydroxalog **2** over the parent, demonstrating that replacement of the labile tertiary benzylic position in ramelteon with a trisubstituted hydroxylamine significantly improves the metabolic stability, consistent with our earlier matched molecular pair analysis.^28^ The activity of **2** and ramelteon **1** against the melatonin receptors MT1 and MT2 was then assessed by means of the β-arrestin (tango) assay,^62,63^ in which the drug was incubated across 16 different concentrations in HTLA cells transfected with GPCR constructs followed by the addition of BrightGlo reagent, with the agonist activity subsequently measured as a function of luminescence (Figure 2). Ultimately, hydroxalog **2** exhibited nanomolar activity against MT1 and MT2 (EC_50_ = 59.4 and 495 nM, respectively) but was nevertheless a much weaker agonist than the picomolar ramelteon **1** (0.0597 and 59.4 nM).

**Figure 2:**
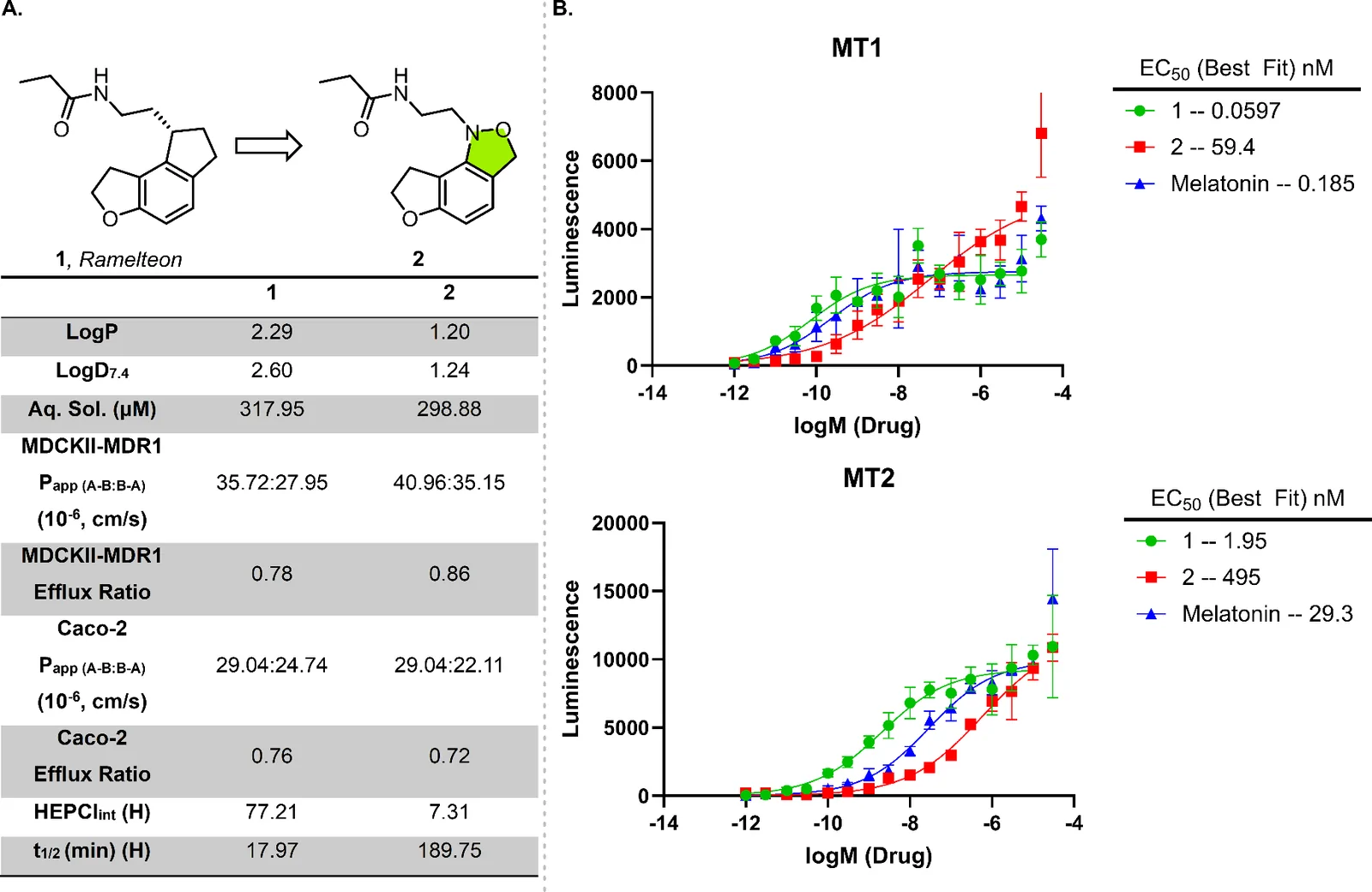
(a) *In vitro* absorption, distribution, metabolism, and excretion (ADME) profiles of **1** and **2**. Abbreviations: H, human; HEPCl_int_, intrinsic clearance in hepatocytes; *t*_1/2_, half-life; MDCKII, Madin–Darby canine kidney; MDR1, multidrug resistance 1 (or P-glycoprotein); *P*_app_, apparent permeability. (b) Agonist activity of **1** and **2** against MT1 and MT2 via the β-arrestin (tango) assay^62,63^ evaluated across 16 different concentrations. IC_50_ values (nM) are reported beside the dose–response curves and represent the mean (best fit).

To investigate the reduction in potency, we performed a computation-based conformational analysis of ramelteon **1** and hydroxalog **2**. To this end, a conformer search employing GOAT^64^ with the semiempirical tight binding method (GFN2-xTB)^65,66^ and analytical linearized Poisson–Boltzmann (ALPB)^67^ implicit water solvation yielded global minima that were further optimized using DFT at the PBE0-D3/def2-TZVP^68–72^ level of theory with CPCM water solvation^72,73^ using Orca 6.1.1.^74,75^ This workflow furnished **48** and **49**, the ground-state conformers of **1** and **2**, respectively, which were validated through vibrational frequency calculations (Figure 3a). Further verification of **48** and **49** was achieved through additional conformer searches using GOAT in the gas phase as well as through CREST^76–78^ with optimization at the AIMNet2­(ωB97M-D3)^79,80^ level of theory using Rowan (see the SI for discussion of the computational workflow).^81^ Consistent with expectation and an X-ray crystal structure of an earlier 1,3-dihydro-2,1-benzisoxazole,^17^ the hydroxylamine nitrogen in **49** is pyramidal with a 334° sum of bond angles.

**Figure 3:**
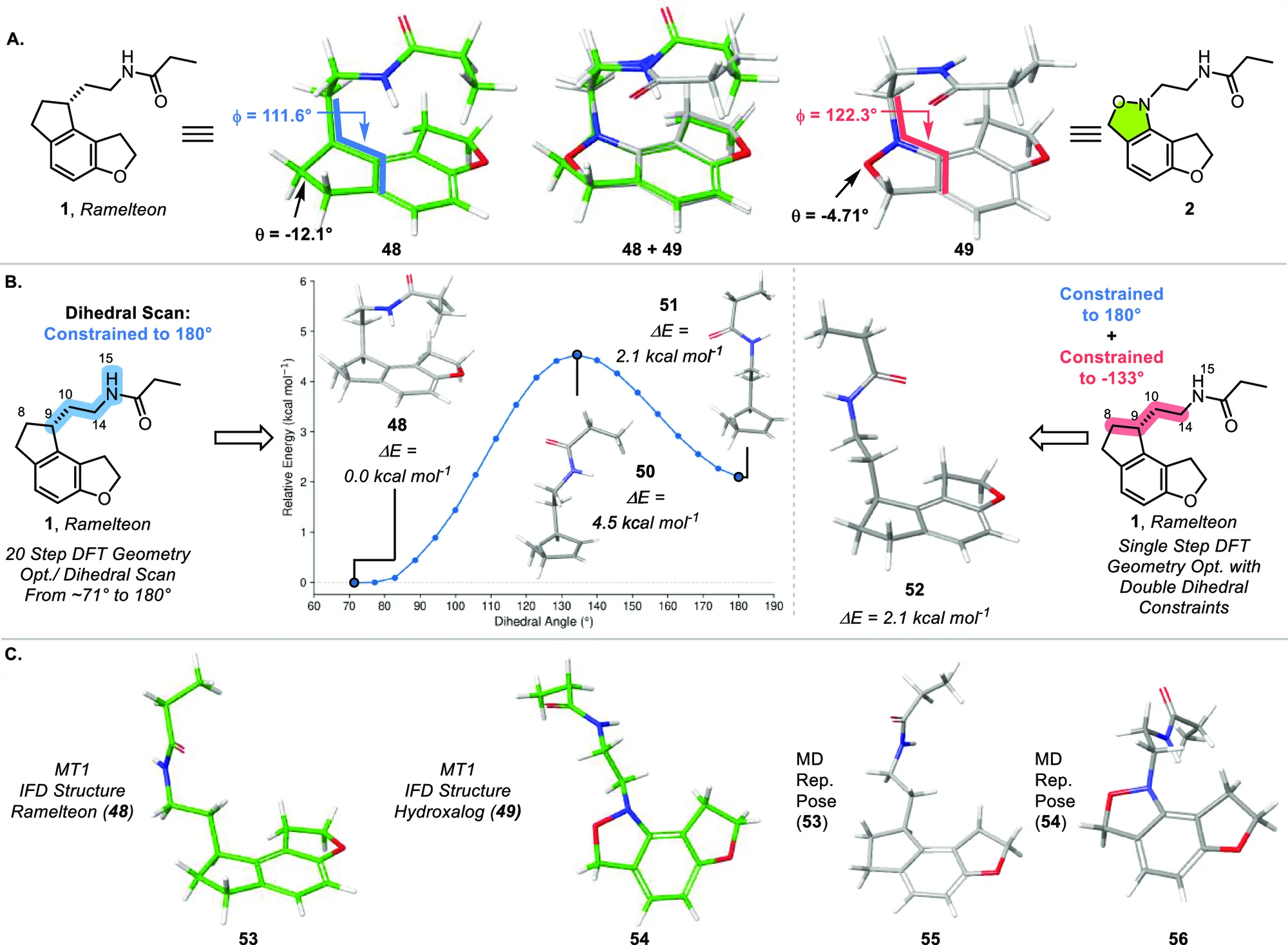
Computational investigation of the structural differences between ramelteon **1** and hydroxalog **2**. All DFT calculations were performed at the PBE0-D3/def2-TZVP^68–72^ level of theory. Docking structures were generated through IFD using Schrödinger 2025.^84–88^ (a) Ground-state conformations of **1** and **2** generated through GOAT XTB^64,65^ with DFT geometry optimization using Orca.^74,75^ (b) Dihedral scan of the ethylene linker in **1** generated by optimization of a structure resembling bound and unbound crystal structures. (c) Induced fit docked (green) and MD representative poses (grey) ligand structures of **1** and **2**.

The ground-state structures **48** and **49** overlay well (Figure 3a, middle), and it is evident that the overall shape of the tricyclic core and side chain is conserved. Closer inspection of these structures reveals minor differences in the conformations of the cyclopentene and isoxazoline rings coupled with opposite orientations of the side-chain amide group, as shown in the overlaid structure (Figure 3a, middle). More precisely, both rings adopt an envelope conformation, but with varying degrees of deviation of the flap from the mean plane. Thus, in **48** the flap is lowered 12.3° below the mean plane of the cyclopentene ring, while in **49** the flap subtends an angle of 4.71° with respect to the mean plane of the isoxazoline ring. Satisfyingly, while the conformation of the five-membered rings in **48** and **49** differs slightly, which we attribute to the differing torsional interactions around the two rings, the exocyclic bond to the side chain is pseudoequatorial for both **48** (torsion angle Φ = 111.6° for CH_2_–CH–C=C highlighted in blue) and **49** (torsion angle Φ = 122.3° for CH_2_–N–C=C highlighted in red) (Figure 3A). Therefore, despite slight differences in conformation and torsion angle, the side chains of **48** and **49** occupy the same space, supporting the design hypothesis.

In both **48** and **49**, even in the presence of implicit water, the side chain adopts a gauche conformation and folds over the tricyclic core. However, while the side chains of **48** and **49** occupy the same overall space, the orientation of the amide group is reversed from **48** to **49**, possibly reflecting a favorable parallel alignment of the amide and hydroxylamine dipoles in the latter.^82^ To rule out local minima entrapment and validate the gauche conformation, we generated the anti conformer through a 20 step DFT-based relaxed dihedral scan (PBE0-D3/def2-TZVP) in which the side-chain dihedral angle (N15–C14–C10–C9, Figure 3b, left) of ground-state conformer **48** was sequentially rotated and locally optimized from 71.4° to 180°. The scan showed that the system passes through an eclipsed conformation (**50**) with a barrier of 4.5 kcal mol^–1^ before reaching the anti conformation (**51**) which is 2.1 kcal mol^–1^ higher in energy than the ground-state gauche conformation. To further rule out the anti conformation as a possible global minimum, an additional optimization was performed in which the side-chain dihedral was again constrained to 180° with an additional dihedral constraint of −133° about C14–C10–C9–C8 (Figure 3b, right), thereby generating a structure with an overall side-chain conformation more closely resembling that found crystallographically in the ramelteon–MT1 complex^43^ and isolated ligand structures.^83^ The so-generated conformer **52** was also 2.1 kcal mol^–1^ higher in energy than the gauche conformer. Together, these results support the initially computed gauche conformer as a true global energy minimum.

Interestingly, while a gas-phase conformer search and optimization sequence furnished an equivalent structure to **48** for ramelteon, the gas-phase minimum for hydroxalog **2** was found to be 2.7 kcal mol^–1^ higher in energy than **49**. Examination of the gas-phase and implicit-water-solvated structures for isoxazoline **2** (see the SI) shows the primary difference to reside in the orientation of the amide, which in the gas phase has the N–H directed toward the isoxazoline oxygen at a distance of 3.0 Å, suggesting an intramolecular hydrogen bond that is disrupted upon water solvation.

Given that **1** adopts an approximately anti conformation of the side chain in its complex with MT1, we looked for differences in ligand conformational strain energy (LCSE) as a possible explanation for the potency difference between **1** and **2**. Accordingly, ground-state conformers **48** and **49** were subjected to induced-fit docking to MT1 ^43^ using Schrödinger with Prime refinement,^84–88^ furnishing top poses **53** and **54**, respectively (Figure 3c). Single-point energy calculations at the same level of theory revealed that **54**, with an LCSE of 9.7 kcal mol^–1^, is only 0.5 kcal mol^–1^ higher in energy than ramelteon in **53** with its LCSE of 9.2 kcal mol^–1^, demonstrating that LCSE is likely not the primary contributor to the difference in potency. In comparison, the same calculations conducted in the gas phase revealed an energy difference of 2.8 kcal mol^–1^ between **53** and **54** with LCSEs of 11.7 and 14.4 kcal mol^–1^, respectively. This difference is nearly identical to the previously described intramolecular hydrogen bond in the gas-phase conformation of **2**, which underlines the importance of solvation in these calculations. Due to the flexibility of GPCRs such as MT1,^89,90^ this method alone may not be robust enough to definitively show key binding interactions, but it is sufficient to examine and compare general ligand conformations, especially when used in conjunction with molecular dynamics (MD) simulations. Accordingly, the docked poses **53** and **54** were solvated in an orthorhombic box of SPC water before being subjected to a 100 ns MD simulation using Desmond^91^ in Schrödinger. As anticipated, ramelteon **53** remains in the binding pocket, undergoing minimal conformational adjustment during the course of the simulation, generating the representative pose **55** with an extended anti conformation of the side chain. On the other hand, hydroxalog **54** rapidly inverts at nitrogen and converges on structure **56** in which the side chain has a skew conformation (Figure 3c). This conformational difference between the side chains of **1** and **2** is suggestive of a gauche effect in **2** arising from the presence of the 1,2-diamino functionality, stabilizing the gauche conformation over and above the extended conformation as seen in docked structures **53** and **54**. Overall, while hydroxalog **2** and ramelteon **1** occupy the same binding pocket and retain a similar conformation about the tricyclic core, **2** has a different conformation of the side chain due to the presence of the two electronegative vicinal nitrogen atoms. Given the side chain’s importance in binding of **1**,^43^ the apparent gauche preference of the side chain in **2** likely drives the observed reduction in potency (Figure 3c).

Overall, methods for the synthesis of the rare 1,3-dihydro-2,1-benzisoxazole nucleus based on Pd- and Cu-mediated C–N bond-forming cyclizations of readily accessible precursors have been developed using conditions familiar to academic and industrial medicinal chemistry laboratories. Hydroxalog **2** exhibited nanomolecular activity and showed significantly improved metabolic stability, as represented by a 10-fold reduction in clearance in human hepatocytes and an increased half-life to over 3 h from only ∼18 min in ramelteon **1**. The decreased potency of **2** toward MT1/2 was determined to be the result of side-chain conformational changes stemming from a strong gauche effect in the 1,2-diamino system.

## Safety Statement

Although no safety hazards were encountered during the synthesis, the preparation of intermediate **39** involves the use of the highly pyrophoric *t*-BuLi, and thus, extreme caution and the rigorous exclusion of moisture should be exercised when conducting this synthetic step.

## Supplementary Material



## Notes

Full experimental details, copies of NMR spectra, additional *in vitro* ADMET data, and computational details (PDF)
