# Supplementary material for: Synthesis of 1,3-Dihydro-2,1-benzisoxazoles by Intramolecular C–N Coupling: Evaluation as an Indane Bioisostere
Source: ACS Med Chem Lett. 2026 Sep 27;17(10):2260–8. doi: 10.1021/acsmedchemlett.6c00429 (PMC13656276; doi:10.1021/acsmedchemlett.6c00429)
Supplement: Supplementary file 1 [file ml6c00429_si_001.pdf]

SUPPORTING INFORMATION

Synthesis of 1,3-Dihydro-2,1-Benzisoxazoles by Intramolecular C-N Coupling: Evaluation as an Indane Bioisostere

Authors: Thomas D. Beckler[1,2] and David Crich[1,2,3*]

1. Innovations in Drug Discovery Program, Department of Pharmaceutical and Biomedical Sciences, University of Georgia, 250 West Green Street, Athens, GA 30602, USA.

2. Department of Chemistry, University of Georgia, 302 East Campus Road, GA 30602, USA.

3. Complex Carbohydrate Research Center, University of Georgia, 315 Riverbend Road, Athens, GA, 30602, USA.

*Correspondence to: david.crich@uga.edu

	TABLE OF CONTENTS	PAGE	SPECTRA
1.	**General Experimental …**	S4	
2.	**Development of Cu and Pd Cross-Couplings …**	S5-32	
	– General Procedures A-D	S5-7	
	– Pd Optimization (**Figure S1**) Cu Optimization (**Figure S2**)	S8	
	– *tert*-Butyl ((2-bromobenzyl)oxy)carbamate (**17**)	S9	S69-70
	– *tert*-Butyl benzo[*c*]isoxazole-1(3*H*)-carboxylate (**18**)	S10	S71-72
	– *tert*-Butyl ((2-bromo-3-methylbenzyl)oxy)carbamate (**19**)	S11-12	S75-76
	– *tert*-Butyl 7-methylbenzo[*c*]isoxazole-1(3*H*)-carboxylate (**20**)	S13	S77-78
	– *tert*-Butyl ((4-bromo-2,3-dihydrobenzofuran-5-yl)methoxy)carbamate (**21**)	S14	S79-80
	– *tert*-Butyl 7,8-dihydrobenzofuro[4,5-*c*]isoxazole-1(3*H*)-carboxylate (**22**)	S15	S81-82
	– *tert*-Butyl ((2-bromo-3-nitrobenzyl)oxy)carbamate (**S1**)	S16	S83-84
	– *tert*-Butyl 7-nitrobenzo[c]isoxazole-1(3H)-carboxylate (**23**)	S17	S85-86
	– *tert*-Butyl ((3-bromopyridin-2-yl)methoxy)carbamate (**S2**)	S18	S87-88
	– *tert-B*utyl isoxazolo[4,3-*b*]pyridine-1(3*H*)-carboxylate(**24**)	S19	S89-90
	– 2-Bromo-3-(bromomethyl)pyrazine (**S3**)	S20-21	S93-94
	– *tert*-Butyl isoxazolo[3,4-*b*]pyrazine-1(3*H*)-carboxylate (**25**)	S22	S95-96
	– *tert*-Butyl (2-bromophenethoxy)carbamate (**S4**)	S23	S97-98
	– *tert*-Butyl 3,4-dihydro-1*H*-benzo[*c*][1,2]oxazine-1-carboxylate (**26**)	S24	S99-100
	– *tert*-Butyl (3-(2-bromophenyl)propoxy)carbamate (**S5**)	S25	S101-102
	– *tert*-Butyl 4,5-dihydrobenzo[*c*][1,2]oxazepine-1(3*H*)-carboxylate (**27**)	S26	S103-104
	– *tert*-Butyl 4-(benzo[*c*]isoxazol-1(3*H*)-ylmethyl)piperidine-1-carboxylate (**31**)	S27-28	S105-106
	– *tert*-Butyl (2-(7-methylbenzo[*c*]isoxazol-1(3*H*)-yl)ethyl)carbamate (**33**)	S29-30	S107-108
	– 1-(Cyclopropylmethyl)-1,3-dihydroisoxazolo[4,3-*b*]pyridine (**35**)	S31-32	S109-110
3.	**Synthesis of the Hydroxalog (2) …**	S32-45	
	– *tert*-Butyl (2,3 dihydrobenzofuran-4-yl)carbamate (**37**) …	S33-34	S111-112
	– *tert*-Butyl (5-bromo-2,3-dihydrobenzofuran-4-yl)carbamate (**38**)	S35	S113-114
	– *tert*-Butyl (5-formyl-2,3-dihydrobenzofuran-4-yl)carbamate (**39**)	S36-37	S115-116
	– 4-Amino-2,3-dihydrobenzofuran-5-carbaldehyde (**40**)	S38	S117-118

	– 4-Bromo-2,3-dihydrobenzofuran-5-carbaldehyde (**41**)	S39	S119-120
	– *tert*-Butyl ((4-bromo-2,3-dihydrobenzofuran-5-yl)methoxy)carbamate (**44**)	S40-41	S123-124
	– *tert*-Butyl (2-(7,8-dihydrobenzofuro[4,5-c]isoxazol-1(*3H*)-yl)ethyl)carbamate (**46**)	S42-43	S125-126
	– *N*-(2-(7,8-Dihydrobenzofuro[4,5-c]isoxazol-1(*3H*)-yl)ethyl)propionamide (**2**)	S44-45	S127-128
**4.**	**Biochemical Data**	S46-S54	
	– Acknowledgement, LogP, LogD, Aq. Sol.	S46	
	– MDCKII-MDR1 Membrane Permeability	S47	
	– Caco-2 Membrane Permeability	S48	
	– Metabolic Stability in Hepatocytes	S49	
	– MT1/MT2 Agonist Functional Data	S50-54	
	– UHPLC Trace of **2**	S55	
**4.**	**Computational Workflow and Discussion**	S56-60	
	– Workflow and Discussion	S56-60	
	– XYZ Coordinates of **48, 49, 51, 52**	S61-68	
**5.**	**Experimental Spectra**	S69-128	
**6.**	**References**	S129-131	

General Experimental

All reactions were conducted in oven dried glassware capped with a rubber septum under an argon atmosphere unless otherwise stated. All organic solutions were concentrated under reduced pressure on a rotary evaporator and water bath. Flash-column chromatography was performed using silica gel cartridges (Sorbent Tech Purity Flash Cartridges Granular Silica Gel 60Å, 40-75μm) on a Teledyne Isco CombiFlash Next Gen 300+. Thin-layer chromatography (TLC) was carried out with 250 μm glass back silica (XHL) plates with fluorescent indicator (254 nm). TLC plates were visualized by exposure to ultraviolet light (UV) and/or submersion in ceric ammonium molybdate solution (CAM) followed by heating on a hot plate (120 °C, 10-15 s). For compounds that could not be detected on TLC using the above methods, the TLC plate was exposed to I_2.

All final compounds are >95% pure by HPLC analysis.

Materials

Commercial solvents and reagents were used as received without further purification. Reagents and solvents were purchased from Sigma-Aldrich or Oakwood. Starting materials were purchased from Ambeed or AABlocks.

Instrumentation

^{1}H and ^{13}C Nuclear magnetic resonance (NMR) spectra of all compounds were obtained at 500 and 126 MHz respectively, in $CDCl_3$ (δ 7.26 and 77.16 ppm, respectively). Multiplicities are abbreviated as follows: s (singlet), multiplet (m), br (broad), d (doublet), t (triplet), q (quartet), p (pentet/quartet). High resolution mass spectra were obtained on an Orbitrap using electrospray ionization (ESI). Melting points were determined via capillary melting point apparatus. IR spectra were recorded using Ge/KBr.

Safety Statement

Although no safety hazards were encountered, the preparation of compound **39** involves the use of the highly pyrophoric *t*-BuLi and thus extreme caution and the rigorous exclusion of moisture should be exercised when conducting this synthetic step.

Development of Cu and Pd Cross-Couplings

General Procedures:

General Procedure A

To a solution of methyl arene dissolved in $PhCF_3$ [0.3M] was added sequentially AIBN (0.2 eq) and NBS (1 eq) followed by heating to 80 ℃ under nitrogen. The reaction was stirred until consumption of the methyl arene was observed via TLC before being diluted in Et_2O, filtered through cotton, and concentrated under reduced pressure. The crude residue was purified via flash column chromatography on silica to furnish the corresponding benzyl bromide.

General Procedure B

N-Boc hydroxylamine (1 eq) was dissolved in 70% EtOH [0.3M] before NaOH (1 eq, flake) was added, followed by a solution of the corresponding benzyl bromide (1 eq, 2M in THF or EtOH) and heating to 80 ℃. The reaction was stirred until consumption of the benzyl bromide was observed by TLC, after which time the mixture was cooled to room temperature, diluted in EtOAc, and quenched with NH_4Cl (10 mL $mmol^{-1}$). The aqueous layer was extracted with EtOAc (3 x 5 mL $mmol^{-1}$) and the combined organic layers were dried over Na_2SO_4, filtered, and concentrated in vacuo. The crude residue was further purified via flash column chromatography on silica.[1]

General Procedure C

To a dry flask containing substrate was added SPhos (0.2 eq), Pd SPhos G3 (0.1 eq), and NaOtBu (1.3 eq). The flask was vacuum purged, flushed with nitrogen, and the

contents suspended in anhydrous Toluene [0.1M] under nitrogen. The reaction mixture was heated to 100 ℃ and stirred until consumption of SM was observed by TLC, after which point, the crude mixture was cooled to room temperature, diluted in Et_2O, filtered through celite, and concentrated in vacuo. The crude reaction residue was then purified by flash column chromatography on silica.

General Procedure D

CuI (1 eq) and CsOAc (5 eq) were added to an oven dried flask which was vacuum purged and backfilled with nitrogen. The substrate was dissolved in dry DMSO [0.2M] and degassed with nitrogen before being added to the sealed vessel and the mixture heated to 90 ℃. After consumption of the starting material by TLC, the reaction mixture was cooled to room temperature, diluted in Et_2O, and quenched with NH_4Cl (20 mL $mmol^{-1}$). The aqueous solution was extracted with Et_2O (5 x 5 mL $mmol^{-1}$)and the combined organics washed with $Na_2S_2O_3$ (1 x 10 mL $mmol^{-1}$), dried over Na_2SO_4, filtered, concentrated in vacuo and the crude residue purified by flash column chromatography on silica.

Bisulfite Workup

If aldehyde byproducts could not be removed during column chromatography, the post-column product mixture was dissolved in MeOH [0.3M] and diluted in sat. $NaHSO_3$ (4:1 v:v). The mixture was shaken vigorously in a separtory funnel for 60 seconds, while venting periodically. The mixture was diluted in H_2O (1:1 v:v w.r.t. $NaHSO_3$) before being extracted twice with EtOAc (1:1 v:v w.r.t. $NaHSO_3$). The organic layers were combined,

dried over Na_2SO_4, filtered, and concentrated under reduced pressure to furnish the isolated product.[2]

Optimization of Pd/Cu Couplings:

Figure S1. Pd Optimization

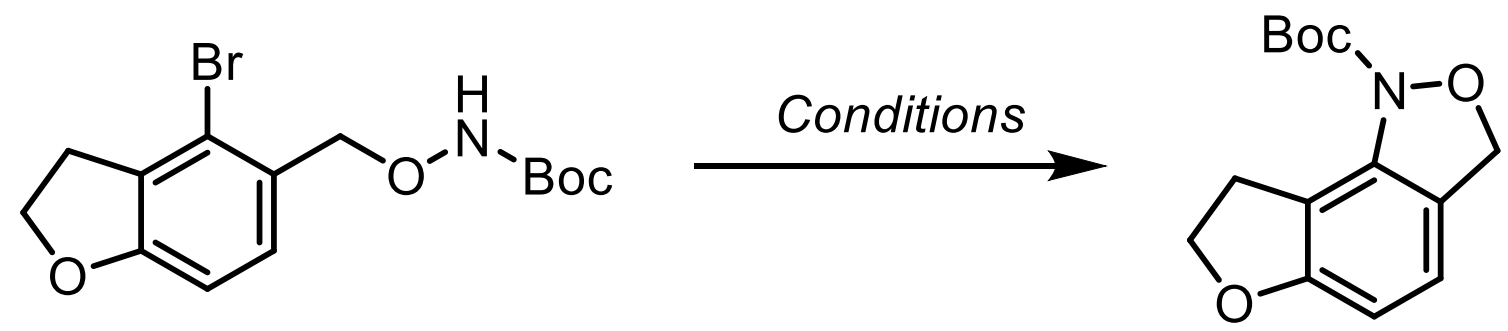

21 **22**

Pd[1]	Ligand	Base	Solvent/Temp	Yield
Pd SPhos G3 (0.1 eq)	SPhos (0.2 eq)	NaOtBu (1.3 eq)	PhMe 100 ℃	28%
Pd_2dba_3 (0.05 eq)	P(2-furyl)$_3$ (0.2 eq)	Cs_2CO_3 (2 eq)	PhMe 100 ℃	19%
Pd_2dba_3 (0.05 eq)	P(*o*-tolyl)$_3$ (0.2 eq)	Cs_2CO_3 (4 eq)	PhMe 100 ℃	19%
Pd_2dba_3 (0.025 eq)	XantPhos (0.05 eq)	Cs_2CO_3 (2 eq)	PhMe 100 ℃	Trace

Figure S2. Cu Optimization

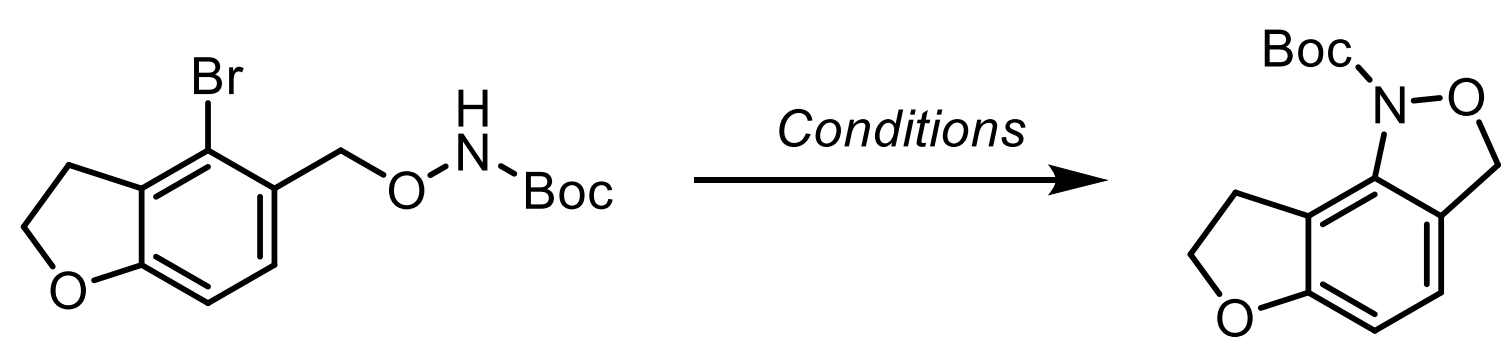

21 **22**

Cu	Base[1]	Solvent/Temp	Yield
CuI (2 eq)	CsOAc	DMSO 90 ℃	30%
CuI (1 eq)	CsOAc	DMSO 90 ℃	62%
CuI (0.5 eq)	CsOAc	DMSO 90 ℃	38%
CuI (0.1 eq) + DMEDA (0.2 eq)	Cs_2CO_3	THF:H_2O rt	Trace

[1] All reactions conducted with 5 eq of base.

***tert*-Butyl ((2-bromobenzyl)oxy)carbamate (17):**

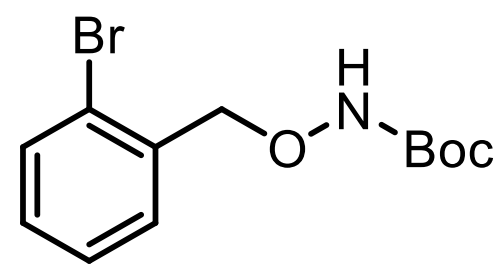

2-Bromobenzyl bromide (0.150 g, 1.13 mmol, 1 eq) was converted to the title compound **17** according to the general procedure B and purified via flash column chromatography on silica (eluent: 15:85 EtOAc:Hexanes) to yield the product (0.197 g, 0.654 mmol, 58%) as a colorless syrup.

TLC: R_f = 0.43 (20:80 EtOAc:Hexanes; UV, CAM)

^{1}H NMR (500 MHz, $CDCl_3$): δ 7.57 (d, *J* = 7.9 Hz, 1H), 7.48 (d, *J* = 7.6 Hz, 1H), 7.32 (t, *J* = 7.5 Hz, 1H), 7.19 (m, 2H), 4.99 (s, 2H), 1.47 (s, 9H).

^{13}C NMR (126 MHz, $CDCl_3$): δ 156.9, 135.4, 133.0, 131.2, 130.1, 127.6, 124.4, 82.0, 77.8, 28.3.

HRMS-ESI (m/z): $[M+Na]^+$ calculated for $[C_{12}H_{16}O_3NBrNa]^+$: 324.0206, found: 324.0195.

***tert*-Butyl benzo[*c*]isoxazole-1(3*H*)-carboxylate (18):**

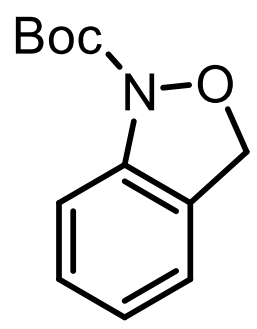

Compound **17** was converted to the title compound **18** according to the general procedures:

<u>General Procedure C (Pd)</u>: Prepared from **17** (0.372 g, 1.24 mmol, 1 eq) and purified via flash column chromatography on silica (eluent: 10:90 EtOAc:Hexanes) to yield the title compound **18** (0.155 g, 0.700 mmol, 57%) as a yellow oil.

<u>General Procedure D (Cu)</u>: Prepared from **17** (0.100 g, 0.332 mmol, 1 eq) and purified via flash column chromatography on silica (eluent: 20:80 Et_2O:Hexanes) to yield the title compound **18** (0.0560 g, 0.252 mmol, 76%) as a yellow oil.

TLC: R_f = 0.49 (20:80 EtOAc:Hexanes; UV, CAM)

^{1}H NMR (500 MHz, $CDCl_3$): δ 7.41 (d, *J* = 8.0 Hz, 1H), 7.25 (t, *J* = 7.9 Hz, 1H), 7.15 (d, *J* = 7.4 Hz, 1H), 7.06 (t, *J* = 7.5 Hz, 1H), 5.35 (s, 2H), 1.59 (s, 9H).

^{13}C NMR (126 MHz, $CDCl_3$): δ 151.7, 138.0, 128.5, 127.5, 124.1, 121.4, 113.3, 83.4, 72.2, 28.5.

HRMS-ESI (m/z): $[M+Na]^+$ calculated for $[C_{12}H_{15}O_3NNa]^+$: 244.0944, found: 244.0936.

***tert*-Butyl ((2-bromo-3-methylbenzyl)oxy)carbamate (19):**

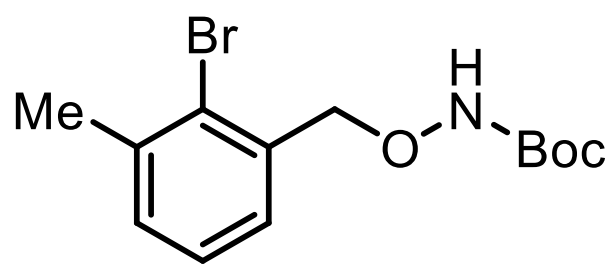

2-Bromo-1,3-dimethylbenzene was converted to 2-bromo-1-(bromomethyl)-3-methylbenzene according to general procedure A and purified via flash column chromatography on silica (eluent: 100 Hexanes) to yield the product (1.01 g, 3.86 mmol, 61%) as an amber oil.

TLC: R_f = 0.41 (100 Hexanes; UV)

^{1}H NMR (500 MHz, $CDCl_3$): δ 7.31 – 7.26 (m, 1H), 7.19 (d, *J* = 4.1 Hz, 2H), 4.65 (s, 2H), 2.44 (s, 3H).

^{13}C NMR (126 MHz, $CDCl_3$): δ 139.6, 137.5, 131.2, 128.9, 127.4, 127.2, 34.8, 23.9.

2-Bromo-1-(bromomethyl)-3-methylbenzene was converted to the title compound **19** according to general procedure B and purified via flash column chromatography on silica (eluent: 12:88 EtOAc:Hexanes) to yield the product (0.680 g, 2.16 mmol, 57%) as a colorless syrup.

TLC: R_f = 0.44 (20:80 EtOAc:Hexanes; UV, CAM)

^{1}H NMR (500 MHz, $CDCl_3$): δ 7.29 (m, 1H), 7.24 – 7.19 (m, 2H), 7.16 (s, 1H), 5.01 (s, 2H), 2.43 (s, 3H), 1.47 (d, *J* = 1.6 Hz, 9H).

^{13}C NMR (126 MHz, $CDCl_3$): δ 156.9, 138.9, 135.8, 130.9, 128.5, 127.1, 126.8, 82.0, 78.5, 28.3, 23.8.

HRMS-ESI (m/z): $[M+Na]^+$ calculated for $[C_{13}H_{18}BrNNaO_3N_2]^+$: 338.0362, found: 338.0355.

tert-Butyl 7-methylbenzo[c]isoxazole-1(3H)-carboxylate (20):

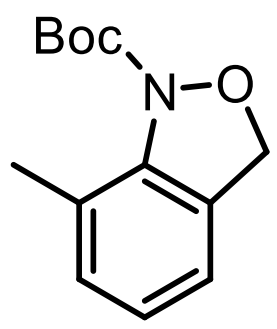

Compound **19** was converted to the title compound **20** according to the general procedures:

General Procedure C (Pd): Prepared from **19** (0.0980 g, 0.310 mmol, 1 eq) and purified via flash column chromatography on silica (eluent: 10:90 Acetone:Hexanes) to yield the title compound **20** (0.0590 g, 0.251 mmol, 81%) as a colorless syrup.

General Procedure D (Cu): Prepared from **19** (0.350 g, 1.11 mmol, 1 eq) and purified via flash column chromatography on silica (eluent: 20:80 Et_2O:Hexanes) to yield the title compound **20** (0.160 g, 0.670 mmol, 60%) as a colorless syrup.

TLC: R_f = 0.55 (20:80 EtOAc:Hexanes; UV, CAM)

^{1}H NMR (500 MHz, $CDCl_3$): δ 7.08 (d, *J* = 4.9 Hz, 2H), 7.03 (t, *J* = 4.4 Hz, 1H), 5.14 (s, 2H), 2.37 (s, 3H), 1.53 (s, 9H).

^{13}C NMR (126 MHz, $CDCl_3$): δ 156.6, 140.4, 132.4, 130.3, 127.8, 126.2, 118.4, 83.5, 73.1, 28.3, 19.1.

HRMS-ESI (m/z): $[M+Na]^+$ calculated for $[C_{13}H_{17}O_3NNa]^+$: 258.1101, found: 258.1100.

***tert*-Butyl ((4-bromo-2,3-dihydrobenzofuran-5-yl)methoxy)carbamate (21):**

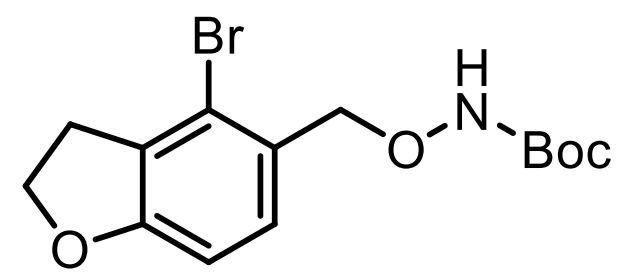

Compound **43** (see S40) was converted to the title compound **21** according to general procedure B and purified via flash column chromatography on silica column chromatography (eluent: 20:80 EtOAC:hexanes) to afford the product (0.270 g, 0.797 mmol, 53%) as a white crystalline solid.

TLC: R_f = 0.58 (30:70 EtOAc:Hexanes; UV, CAM)

^{1}H NMR (500 MHz, $CDCl_3$): δ 7.20 (d, *J* = 8.2 Hz, 1H), 7.08 (s, 1H), 6.70 (d, *J* = 9.2 Hz, 1H), 4.91 (s, 2H), 4.63 (t, *J* = 9.4 Hz, 2H), 3.23 (t, *J* = 8.8 Hz, 2H), 1.47 (s, 9H).

^{13}C NMR (126 MHz, $CDCl_3$): δ 160.6, 156.8, 132.0, 129.4, 126.8, 122.0, 108.1, 81.8, 77.4, 71.2, 31.9, 28.3.

HRMS-ESI (m/z): $[M+Na]^+$ calculated for $[C_{14}H_{18}O_4NBrNa]^+$: 366.0311, found: 366.0299.

***tert*-Butyl 7,8-dihydrobenzofuro[4,5-*c*]isoxazole-1(*3H*)-carboxylate (22):**

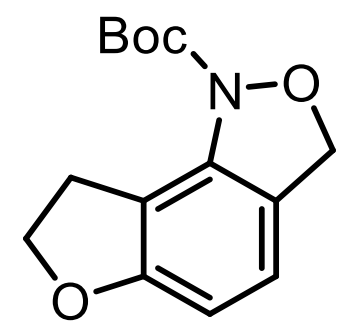

Compound **21** was converted to the title compound **22** according to the general procedures:

General Procedure C (Pd): Prepared from **21** (0.037 g, 0.11 mmol, 1 eq) and purified via flash column chromatography on silica (eluent: 20:80 EtOAc:Hexanes) to yield the title compound **22** (0.0065 g, 0.025 mmol, 23%) as a colorless syrup.

General Procedure D (Cu): Prepared from **21** (0.251 g, 0.733 mmol, 1 eq) and purified via flash column chromatography on silica (eluent: 10:90 Et_2O:Hexanes) to yield the title compound **22** (0.119 g, 0.452 mmol, 62%) as a colorless syrup.

TLC: R_f = 0.66 (30:70 EtOAc:Hexanes; UV, CAM)

^{1}H NMR (500 MHz, $CDCl_3$): δ 6.89 (d, *J* = 8.0 Hz, 1H), 6.55 (d, *J* = 8.0 Hz, 1H), 5.17 (s, 2H), 4.56 (t, *J* = 8.7 Hz, 2H), 3.40 (t, *J* = 8.7 Hz, 2H), 1.56 (s, 9H).

^{13}C NMR (126 MHz, $CDCl_3$): δ 161.7, 154.3, 136.4, 122.1, 120.2, 114.6, 105.7, 83.4, 72.7, 71.7, 30.0, 28.3.

HRMS-ESI (m/z): $[M+Na]^+$ calculated for $[C_{14}H_{17}O_4NNa]^+$: 286.1049, found: 286.1047.

***tert*-Butyl ((2-bromo-3-nitrobenzyl)oxy)carbamate (S1):**

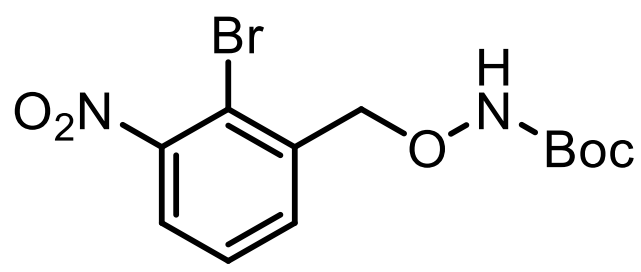

2-Bromo-1-(bromomethyl)-3-nitrobenzene was converted to the title compound **S1** according to general procedure B and purified via flash column chromatography on silica (eluent: 30:70 EtOAc:Hexanes) to yield the product (0.446 g, 1.29 mmol, 76%) as an off-white solid.

TLC: R_f = 0.54 (40:60 EtOAc:Hexanes; UV, CAM)

^{1}H NMR (500 MHz, $CDCl_3$): δ 7.71 (d, *J* = 7.7 Hz, 1H), 7.66 (d, *J* = 8.0 Hz, 1H), 7.45 (t, *J* = 8.6 Hz, 1H), 7.24 (d, *J* = 7.8 Hz, 1H), 5.05 (s, 2H), 1.46 (s, 9H).

^{13}C NMR (126 MHz, $CDCl_3$): δ 156.8, 151.3, 138.9, 133.3, 128.1, 124.6, 115.0, 82.5, 28.2.

HRMS-ESI (m/z): $[M+Na]^+$ calculated for $[C_{12}H_{15}O_5N_2BrNa]^+$: 369.0056, found: 369.0054.

***tert*-Butyl 7-nitrobenzo[*c*]isoxazole-1(3*H*)-carboxylate (23):**

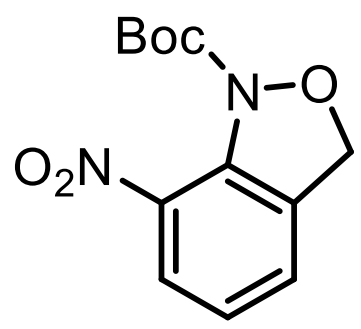

Compound **S2** was converted to the title compound **23** according to the general procedures:

General Procedure C (Pd): Prepared from **S2** (0.0980 g, 0.310 mmol, 1 eq) and purified via flash column chromatography on silica (eluent: 65:35 Et_2O:Hexanes) to yield the title compound **23** (0.0150 g, 0.0564 mmol, 20%) as a yellow oil.

General Procedure D (Cu): Prepared from **S2** (0.0980 g, 0.310 mmol, 1 eq) and purified via flash column chromatography on silica (eluent: 40:60 EtOAc:Hexanes) to yield the title compound **23** (0.0130 g, 0.0500 mmol, 35%) as a yellow oil.

TLC: R_f = 0.30 (40:60 EtOAc:Hexanes; UV, CAM)

^{1}H NMR (500 MHz, $CDCl_3$): δ 7.90 (d, *J* = 8.2 Hz, 1H), 7.47 (d, *J* = 7.4 Hz, 1H), 7.31 (t, *J* = 7.8 Hz, 1H), 5.32 (s, 2H), 1.49 (s, 9H).

^{13}C NMR (126 MHz, $CDCl_3$): δ 153.2, 134.8, 134.0, 126.4, 126.2, 124.2, 85.6, 72.5, 28.0.

HRMS-ESI (m/z): $[M+Na]^+$ calculated for $[C_{12}H_{14}O_5N_2Na]^+$: 289.0795, found: 289.0785.

***tert*-Butyl ((3-bromopyridin-2-yl)methoxy)carbamate (S2):**

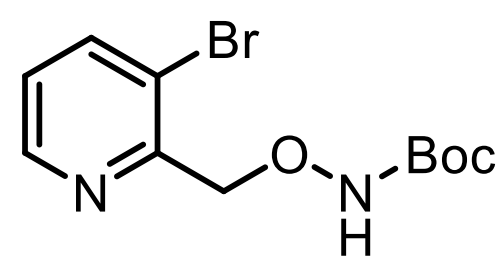

3-Bromo-2-(bromomethyl)pyridine (2.00 g, 7.97 mmol, 1 eq) was converted to the title compound **S2** according to the general procedure B and purified via flash column chromatography on silica (eluent: 10:90 EtOAc:Hexanes) to yield the product (1.03 g, 3.37 mmol, 42%) as a yellow syrup.

TLC: R_f = 0.37 (50:50 EtOAc:Hexanes; UV, CAM)

^{1}H NMR (500 MHz, $CDCl_3$): δ 8.56 (d, *J* = 4.7 Hz, 1H), 7.98 (s, 1H), 7.86 (d, *J* = 8.0 Hz, 1H), 7.14 (dd, *J* = 8.5, 4.5 Hz, 1H), 5.14 (s, 2H), 1.47 (s, 9H).

^{13}C NMR (126 MHz, $CDCl_3$): δ 156.5, 154.8, 148.0, 140.6, 124.3, 120.4, 81.9, 28.4.

HRMS-ESI (m/z): $[M+H]^+$ calculated for $[C_{11}H_{16}O_3N_2Br]^+$: 303.0339, found: 303.0331.

***tert*-Butyl isoxazolo[4,3-*b*]pyridine-1(3*H*)-carboxylate(24):**

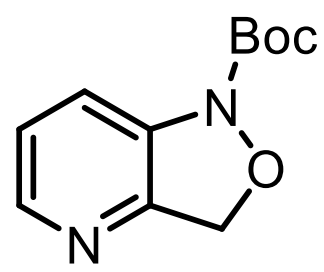

Compound **S2** was converted to the title compound **24** according to the general procedures:

General Procedure C (Pd): Yielded no desired product.

General Procedure D (Cu): Prepared from **S2** (0.500 g, 1.63 mmol, 1 eq) and purified via flash column chromatography on silica (eluent: 30:70 EtOAc:Hexanes) to yield the title compound **24** (0.227 g, 1.02 mmol, 63%) as a colorless syrup.

TLC: R_f = 0.46 (50:50 EtOAc:Hexanes; UV, CAM)

^{1}H NMR (500 MHz, $CDCl_3$): δ 8.23 (d, *J* = 5.0 Hz, 1H), 7.61 (d, *J* = 8.1 Hz, 1H), 7.16 (dd, *J* = 8.3, 4.8 Hz, 1H), 5.36 (s, 2H), 1.59 (s, 9H).

^{13}C NMR (126 MHz, $CDCl_3$): δ 151.4, 149.2, 145.0, 132.2, 122.8, 119.7, 84.1, 72.0, 28.4.

HRMS-ESI (m/z): $[M+H]^+$ calculated for $[C_{11}H_{15}O_3N_2]^+$: 223.1077, found: 223.1072.

***tert*-Butyl ((3-bromopyrazin-2-yl)methoxy)carbamate (S3):**

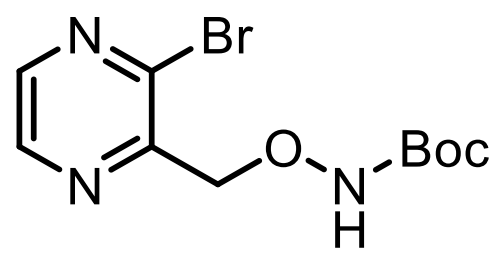

5-Bromo-4-methylpyrazine was converted to 2-bromo-3-(bromomethyl)pyrazine according to general procedure A and purified via flash column chromatography on silica (eluent: 20:80 EtOAc:Hexanes) to yield the product (0.252 g, 0.998 mmol, 35%) as a yellow oil.

TLC: R_f = 0.43 (20:80 EtOAc:Hexanes; UV, CAM)

^{1}H NMR (500 MHz, $CDCl_3$): δ 8.51 (d, *J* = 2.4 Hz, 1H), 8.30 (d, *J* = 2.5 Hz, 1H), 4.69 (s, 2H).

^{13}C NMR (126 MHz, $CDCl_3$): δ 153.2, 144.0, 142.9, 141.4, 31.5.

2-Bromo-3-(bromomethyl)pyrazine was converted to the title compound **S3** according to the general procedure B and purified via flash column chromatography on silica (eluent: 70:30 Et_2O:Hexanes 1% Et_3N) to yield the product (0.145 g, 0.476 mmol, 51%) as a yellow syrup.

TLC: R_f = 0.44 (70:30 Et_2O:Hexanes; UV, CAM)

^{1}H NMR (500 MHz, $CDCl_3$): δ 8.54 (s, 1H), 8.32 (s, 1H), 7.74 (s, 1H), 5.15 (s, 2H), 1.47 (s, 9H).

^{13}C NMR (126 MHz, $CDCl_3$): δ 153.2, 144.0, 142.7, 141.4, 31.5.

HRMS-ESI (m/z): $[M+Na]^+$ calculated for $[C_{10}H_{14}O_3N_3BrNa]^+$: 326.0111, found: 326.0102.

tert-Butyl isoxazolo[3,4-_b_]pyrazine-1(3_H_)-carboxylate (25):

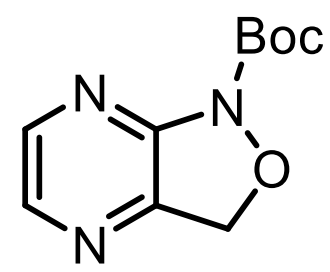

Compound **S3** was converted to the title compound **25** according to the general procedures:

General Procedure C (Pd): Yielded no desired product.

General Procedure D (Cu): Prepared from **S3** (0.212 g, 0.695 mmol, 1 eq) and purified via flash column chromatography on silica (eluent: 40:60 EtOAc:Hexanes) to yield the title compound **25** (0.0360 g, 0.162 mmol, 23%) as a yellow syrup.

TLC: R_f = 0.29 (60:40 Et_2O:Hexanes; UV, CAM)

^{1}H NMR (500 MHz, $CDCl_3$): δ 8.19 (s, 1H), 8.14 (s, 1H), 5.38 (s, 2H), 1.60 (s, 9H).

^{13}C NMR (126 MHz, $CDCl_3$): δ 149.7, 147.4, 144.9, 141.9, 138.9, 84.9, 70.6, 28.3.

HRMS-ESI (m/z): $[M+Na]^+$ calculated for $[C_{10}H_{13}O_3N_3Na]^+$: 246.0849, found: 246.0841.

***tert*-Butyl (2-bromophenethoxy)carbamate (S4):**

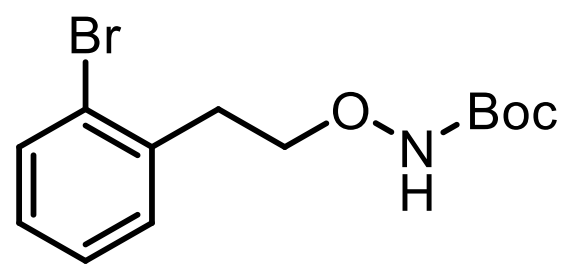

1-Bromo-2-(2-bromoethyl)benzene was converted to the title compound **S4** according to the general procedure B and purified via flash column chromatography on silica (eluent: 15:85 EtOAc:Hexanes) to yield the product (0.211 g, 0.668 mmol, 35%) as a colorless syrup.

TLC: R_f = 0.38 (15:85 EtOAc:Hexanes; UV, CAM)

^{1}H NMR (500 MHz, CDCl$_3$): δ 7.53 (d, *J* = 8.0 Hz, 1H), 7.32 (d, *J* = 7.7 Hz, 1H), 7.24 (d, *J* = 7.6 Hz, 1H), 7.15 (s, 1H), 7.08 (t, *J* = 7.7 Hz, 1H), 4.10 (t, *J* = 6.4 Hz, 2H), 3.11 (t, *J* = 7.1 Hz, 2H), 1.48 (s, 9H).

^{13}C NMR (126 MHz, CDCl$_3$): δ 157.1, 137.6, 133.0, 131.2, 128.3, 127.7, 124.8, 81.9, 75.5, 35.0, 28.3.

HRMS-ESI (m/z): $[M+K]^+$ calculated for $[C_{13}H_{18}O_3NBrK]^+$: 354.0102, found: 354.0101.

tert-Butyl 3,4-dihydro-1_H_-benzo[_c_][1,2]oxazine-1-carboxylate (26):

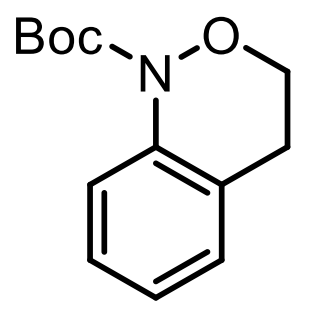

Compound **S4** was converted to the title compound **26** according to the general procedures:

General Procedure C (Pd): Prepared from **S4** (0.0750 g, 0.237 mmol, 1 eq) and purified via flash column chromatography on silica (eluent: 10:90 Acetone:Hexanes) to yield the title compound **26** (0.0900 g, 0.382 mmol, 65%) as a white solid.

General Procedure D (Cu): Prepared from **S4** (0.212 g, 0.695 mmol, 1 eq) and purified via flash column chromatography on silica (eluent: 40:60 EtOAc:Hexanes) to yield the title compound **26** (0.0170 g, 0.0714 mmol, 30%) as a white solid.

TLC: R_f = 0.46 (20:80 EtOAc:Hexanes; UV, CAM)

^{1}H NMR (500 MHz, $CDCl_3$): δ 7.46 (d, _J_ = 8.2 Hz, 1H), 7.16 (dd, _J_ = 19.0, 7.8 Hz, 2H), 7.07 (t, _J_ = 7.4 Hz, 1H), 4.30 (t, _J_ = 6.4 Hz, 2H), 3.02 (t, _J_ = 6.3 Hz, 2H), 1.55 (s, 9H).

^{13}C NMR (126 MHz, $CDCl_3$): δ 153.3, 138.3, 128.9, 126.0, 124.6, 123.9, 122.5, 82.8, 69.6, 28.4, 26.7.

HRMS-ESI (m/z): $[M+Na]^+$ calculated for $[C_{13}H_{17}O_3NNa]^+$: 258.1101, found: 258.1099.

***tert*-Butyl (3-(2-bromophenyl)propoxy)carbamate (S5):**

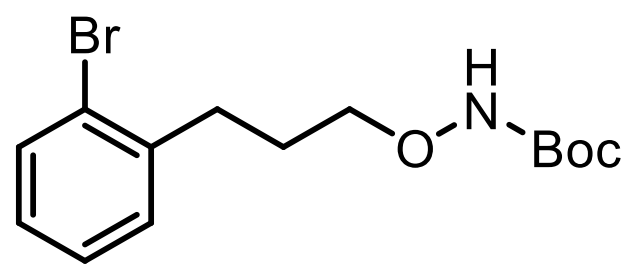

1-Bromo-2-(3-bromopropyl)benzene was converted to the title compound **S5** according to the general procedure B and purified via flash column chromatography on silica (eluent: 15:85 EtOAc:Hexanes) to yield the product (0.360 g, 1.09 mmol, 61%) as a colorless syrup.

TLC: R_f = 0.37 (15:85 Acetone:Hexanes; UV, CAM)

^{1}H NMR (500 MHz, $CDCl_3$): δ 7.51 (d, *J* = 7.9 Hz, 1H), 7.27 – 7.18 (m, 2H), 7.11 (s, 1H), 7.04 (t, *J* = 7.4 Hz, 1H), 3.89 (t, *J* = 6.3 Hz, 2H), 2.84 (t, *J* = 7.8 Hz, 2H), 1.99 – 1.89 (m, 2H), 1.48 (s, 9H).

^{13}C NMR (126 MHz, $CDCl_3$): δ 157.1, 141.1, 133.0, 130.7, 127.9, 127.6, 124.6, 81.8, 75.9, 32.7, 28.4, 28.3.

HRMS-ESI (m/z): $[M+Na]^+$ calculated for $[C_{14}H_{20}BrNNaO_3]^+$: 352.0519, found: 352.0514.

***tert*-Butyl 4,5-dihydrobenzo[*c*][1,2]oxazepine-1(3*H*)-carboxylate (27):**

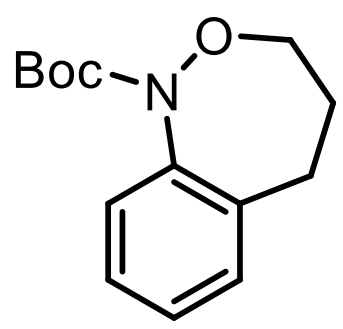

Compound **S5** was converted to the title compound **27** according to the general procedures:

General Procedure C (Pd): Prepared from **S5** (0.100 g, 0.303 mmol, 1 eq) and purified via flash column chromatography on silica (eluent: 5:95 Acetone:Hexanes) to yield the title compound **27** (0.0190 g, 0.0762 mmol, 25%) as a colorless syrup.

General Procedure D (Cu): Prepared from **S5** (0.10 g, 0.30 mmol, 1 eq) and purified via flash column chromatography on silica (eluent: 5:95 Acetone:Hexanes) to yield the title compound **27** (0.0061 g, 0.025 mmol, 8%) as a colorless syrup.

TLC: R_f = 0.43 (15:85 Acetone:Hexanes; UV, CAM)

^{1}H NMR (500 MHz, $CDCl_3$): δ 7.26 (d, *J* = 10.2 Hz, 1H), 7.20 (d, *J* = 6.9 Hz, 1H), 7.15 (q, *J* = 7.4 Hz, 2H), 4.26 (t, *J* = 5.4 Hz, 2H), 3.02 – 2.91 (m, 2H), 1.89 (p, *J* = 5.6 Hz, 2H), 1.50 (s, 9H).

^{13}C NMR (126 MHz, $CDCl_3$): δ 152.9, 141.0, 138.8, 130.0, 127.6, 126.6, 126.5, 81.9, 77.0, 34.0, 28.5, 27.7.

HRMS-ESI (m/z): $[M+Na]^+$ calculated for $[C_{14}H_{19}O_3NNa]^+$: 272.1257, found: 272.1248.

***tert*-Butyl 4-(benzo[*c*]isoxazol-1(3*H*)-ylmethyl)piperidine-1-carboxylate (31):**

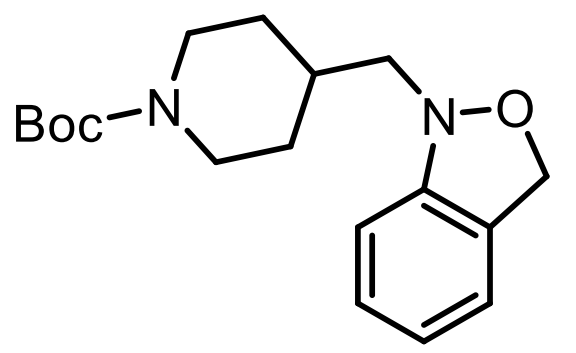

To a stirred solution of compound **18** (0.0510 g, 0.234 mmol, 1 eq) in dry DCE (0.470 mL, 0.500M) at 0 ℃, was added thioanisole (0.0330 mL, 0.280 mmol, 1.2 eq) followed by TFA (0.470 mL, 50% v:v) dropwise. The reaction was stirred for 1 h before being quenched by 10 mL sat. $NaHCO_3$ and extracted 4 x 2 mL Et_2O. The combined organic layers were dried over Na_2SO_4, filtered, and concentrated under reduced pressure to furnish the secondary hydroxylamine.

The crude secondary hydroxylamine was combined with *tert*-butyl 4-formylpiperidine-1-carboxylate (0.150 g, 0.701 mmol, 3 eq), 3Å MS (0.460 g, 2g/mmol), and dissolved in dry MeOH (1.20 mL, 0.300M). The reaction mixture was stirred for 15 minutes before AcOH (0.0400 mL, 0.701 mmol, 3 eq) and $NaBH_3CN$ (0.0440 g, 0.701 mmol, 3 eq) were added sequentially. After 1 h, the reaction was quenched with 10 mL sat. $NaHCO_3$ and extracted 4 x 2 mL Et_2O. The combined organic layers were dried over Na_2SO_4, filtered, and concentrated under reduced pressure. The crude residue was purified via flash column chromatography on silica (eluent: 30:70 Et_2O:Hexanes) to yield the product (0.0480 g, 0.150 mmol, 64%) as a colorless syrup.

TLC: R_f = 0.25 (30:70 EtOAc:Hexanes; UV, CAM)

^{1}H NMR (500 MHz, $CDCl_3$): δ 7.19 (t, *J* = 7.6 Hz, 1H), 7.14 (d, *J* = 7.4 Hz, 1H), 6.99 (t, *J* = 7.4 Hz, 1H), 6.69 (d, *J* = 7.9 Hz, 1H), 5.18 (s, 2H), 4.14 (s, 2H), 3.07 (d, *J* = 6.8 Hz,

2H), 2.75 (br, 2H), 1.99 (m, 1H), 1.89 (d, J = 14.4 Hz, 2H), 1.46 (s, 9H), 1.27 – 1.13 (m, 2H).

^{13}C NMR (126 MHz, $CDCl_3$): δ 155.0, 150.3, 129.4, 128.1, 123.2, 121.6, 110.5, 79.4, 70.5, 65.0, 43.6, 35.0, 30.6, 28.6.

HRMS-ESI (m/z): $[M+Na]^+$ calculated for $[C_{18}H_{26}O_3N_2Na]^+$:341.1836, found: 341.1828.

***tert*-Butyl (2-(7-methylbenzo[*c*]isoxazol-1(3*H*)-yl)ethyl)carbamate (33):**

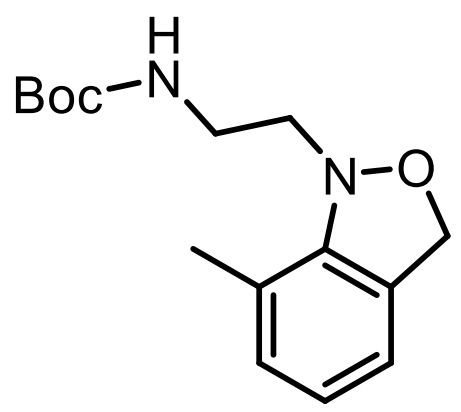

To a stirred solution of compound **20** (0.014 g, 0.060 mmol, 1 eq) in dry DCE (0.12 mL, 0.500M) at 0 ℃, was added thioanisole (0.086 mL, 0.073 mmol, 1.2 eq) followed by TFA (0.12 mL, 50% v:v) dropwise. The reaction was stirred for 30 mins before being quenched by10 mL sat. $NaHCO_3$ and extracted 3 x 2 mL Et_2O. The combined organic layers were dried over Na_2SO_4, filtered, and concentrated under reduced pressure to furnish the secondary hydroxylamine.

The crude secondary hydroxylamine was combined with tert-butyl (2-oxoethyl)carbamate (0.029 g, 0.18 mmol, 3 eq), 3Å MS (0.12 g, 2 g/mmol), and dissolved in dry MeOH (0.20 mL, 0.300M). The reaction mixture was stirred for 15 minutes before AcOH (0.010 mL, 0.18 mmol, 3 eq) and $NaBH_3CN$ (0.011 g, 0.18 mmol, 3 eq) were added sequentially. After 1 h, the reaction was quenched with 10 mL sat. $NaHCO_3$ and extracted 3 x 3 mL Et_2O. The combined organic layers were dried over Na_2SO_4, filtered, and concentrated under reduced pressure. The crude residue was purified via flash column chromatography on silica (eluent: 40:60 Et_2O:Hexanes) to yield the product (0.0097 g, 0.035 mmol, 58%) as a colorless syrup.

TLC: R_f = 0.50 (30:70 EtOAc:Hexanes; UV, CAM)

^{1}H NMR (500 MHz, $CDCl_3$): δ 7.01 (m, 3H), 5.20 (s, 3H), 3.46 (q, *J* = 5.8 Hz, 2H), 3.17 (t, *J* = 5.5 Hz, 2H), 2.23 (s, 3H), 1.44 (s, 9H).

^{13}C NMR (126 MHz, CDCl$_3$): δ 148.2, 130.8, 130.1, 125.0, 123.0, 119.0, 70.5, 56.4, 39.5, 28.5, 17.3.

HRMS-ESI (m/z): [M+Na]$^+$ calculated for [$C_{16}H_{22}O_4N_2Na$]$^+$:329.1472, found: 329.1468.

1-(Cyclopropylmethyl)-1,3-dihydroisoxazolo[4,3-*b*]pyridine (35):

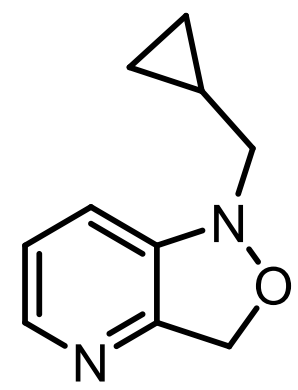

To a stirred solution of compound **24** (0.0500 g, 0.225 mmol, 1 eq) in dry DCE (0.450 mL, 0.500M) at 0 ℃, was added thioanisole (0.0320 mL, 0.270 mmol, 1.2 eq) followed by TFA (0.450 mL, 50% v:v) dropwise. The reaction was stirred for 1 h before being quenched by10 mL sat. $NaHCO_3$ and extracted 5 x 3 mL Et_2O and 4 x 3 mL EtOAc. The combined organic layers were dried over Na_2SO_4, filtered, and concentrated under reduced pressure to furnish the secondary hydroxylamine.

The crude secondary hydroxylamine was combined with cyclopropanecarboxaldehyde (0.0470 g, 0.675 mmol, 3 eq), 3Å MS (0.450 g, 2g/mmol), and dissolved in dry MeOH (0.750 mL, 0.300M). The reaction mixture was stirred for 15 minutes before AcOH (0.0390 mL, 0.675 mmol, 3 eq) and $NaBH_3CN$ (0.0420 g, 0.675 mmol, 3 eq) were added sequentially. After 1 h, the reaction was quenched with 10 mL sat. $NaHCO_3$ and extracted 4 x 3 mL Et_2O. The combined organic layers were dried over Na_2SO_4, filtered, and concentrated under reduced pressure. The crude residue was purified via flash column chromatography on silica (eluent: 20:80 Acetone:Hexanes) to yield the product (0.0130 g, 0.0715 mmol, 32%) as a colorless syrup.

TLC: R_f = 0.68 (50:50 Acetone:Hexanes; UV, CAM)

^{1}H NMR (500 MHz, $CDCl_3$): δ 8.17 (d, *J* = 4.9 Hz, 1H), 7.12 – 7.05 (m, 1H), 7.01 (d, *J* = 8.0 Hz, 1H), 5.21 (s, 2H), 3.16 (d, *J* = 6.8 Hz, 2H), 1.21 – 1.06 (m, 1H), 0.64 – 0.55 (m, 2H), 0.28 (d, *J* = 4.8 Hz, 2H).

^{13}C NMR (126 MHz, $CDCl_3$): δ 152.0, 144.3, 143.1, 122.3, 117.7, 70.6, 63.4, 8.5, 4.0.

HRMS-ESI (m/z): $[M+H]^+$ calculated for $[C_{10}H_{13}ON_2]^+$: 177.1022, found: 177.1018.

Synthesis of the Hydroxalog (2):

tert-Butyl (2,3-dihydrobenzofuran-4-yl)carbamate (37):

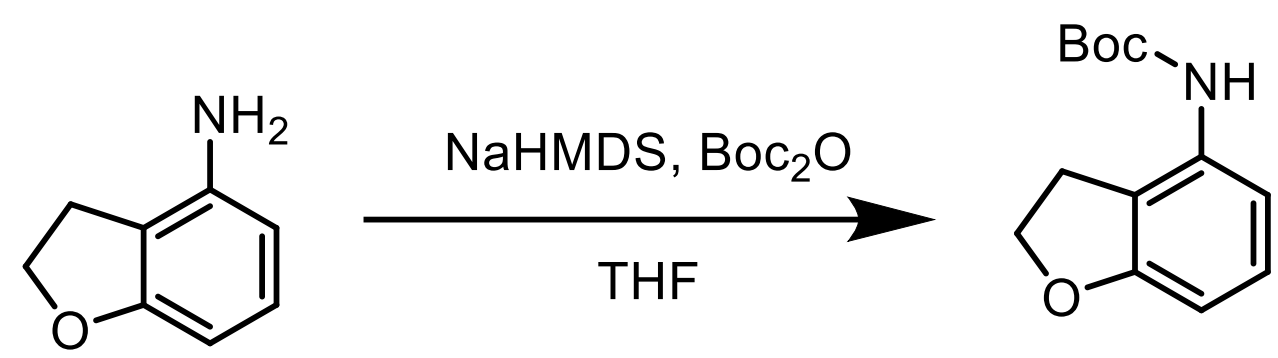

37

2,3-dihydrobenzofuran-4-amine (5.00 g, 37.0 mmol, 1 eq) was dissolved in dry THF (37.0 mL, 1.00M) under Ni, and NaHMDS 1M in THF (81.0 mL, 81.4 mmol, 2.2 eq) was added dropwise and allowed to stir for 15 minutes. A solution of Boc_2O 2M in THF (18.5 mL, 37.0 mmol, 1 eq) was then added dropwise and the reaction was stirred for an hour before being diluted with EtOAc and quenched with 150 mL NH_4Cl. The aqueous phase was extracted 4 x 50 mL EtOAc and the combined organic layers washed with 50 mL brine, dried over Na_2SO_4, filtered, and concentrated under reduced pressure. The crude reaction mixture was purified via flash column chromatography on silica (eluent: 15:85 EtOAc:hexanes) and filtered through activated charcoal to afford the title compound **37** (7.20 g, 30.7 mmol, 83%) as a white crystalline solid.

TLC: R_f = 0.59 (30:70 EtOAc:Hexanes; UV, CAM)

m.p = 122-124℃

^{1}H NMR (500 MHz, $CDCl_3$): δ .25 (d, *J* = 8.8 Hz, 1H), 7.07 (t, *J* = 8.1 Hz, 1H), 6.52 (d, *J* = 7.9 Hz, 1H), 6.18 (s, 1H), 4.57 (t, *J* = 8.7 Hz, 2H), 3.10 (t, *J* = 8.7 Hz, 2H), 1.51 (s, 9H).

^{13}C NMR (126 MHz, $CDCl_3$): δ 160.8, 152.7, 135.2, 129.0, 116.3, 112.0, 105.0, 80.9, 71.1, 28.4, 27.9.

HRMS-ESI (m/z): $[M+Na]^+$ calculated for $[C_{13}H_{17}O_3NNa]^+$: 258.1101, found: 258.1096.

***tert*-Butyl (5-bromo-2,3-dihydrobenzofuran-4-yl)carbamate (38):**

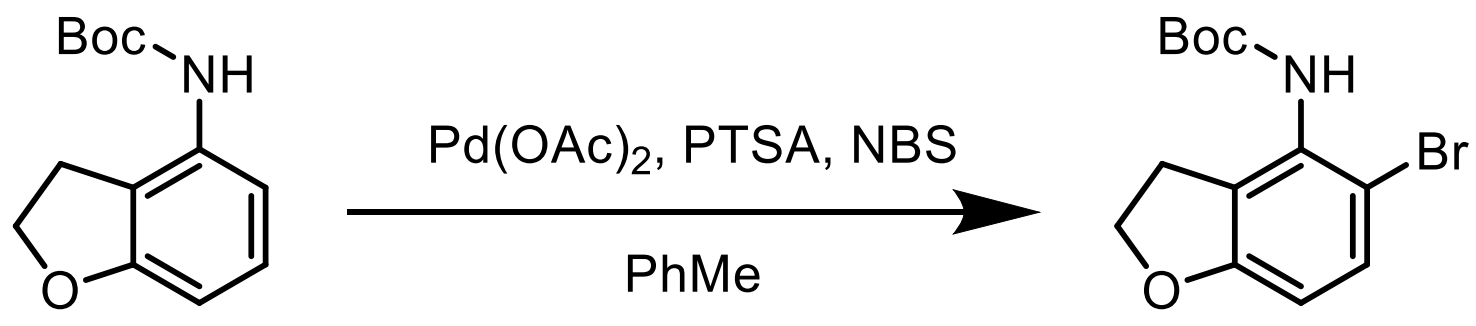

To a solution of **37** (7.80 g, 33.0 mmol, 1 eq) in dry toluene (165 mL, 0.200M) was added sequentially PTSA (3.10 g, 16.5 mmol, 0.5 eq), $Pd(OAc)_2$ (0.370 g, 1.65 mmol, 0.05 eq), and NBS (7.00 g, 39.6 mmol, 1.2 eq).[3] The reaction was stirred for 1 h open to air before it was diluted in diethyl ether and quenched with 100 mL $NaHCO_3$. The aqueous phase was extracted 4 x 75 mL ether and the combined organic layers washed with 100 mL brine, dried over Na_2SO_4, filtered, and concentrated under reduced pressure. The crude reaction mixture was purified via flash column chromatography on silica (eluent: 20:80 EtOAc:hexanes) to afford the title compound **38** (9.60 g, 30.4 mmol, 93%) as an off-white solid.

TLC: R_f = 0.51 (20:80 EtOAc:Hexanes; UV, CAM)

m.p = 139.3℃

^{1}H NMR (500 MHz, $CDCl_3$): δ 7.26 (d, *J* = 9.4 Hz, 1H), 6.52 (d, *J* = 8.4 Hz, 1H), 6.40 (s, 1H), 4.57 (t, *J* = 8.7 Hz, 2H), 3.26 (t, *J* = 8.8 Hz, 2H), 1.50 (s, 9H).

^{13}C NMR (126 MHz, $CDCl_3$): δ 160.8, 152.4, 132.4, 131.5, 125.0, 108.5, 108.1, 81.1, 72.1, 30.2, 28.4.

HRMS-ESI (m/z): $[M+Na]^+$ calculated for $[C_{13}H_{16}O_3NBrNa]^+$: 336.0206, found: 336.0207.

***tert*-Butyl (5-formyl-2,3-dihydrobenzofuran-4-yl)carbamate (39):**

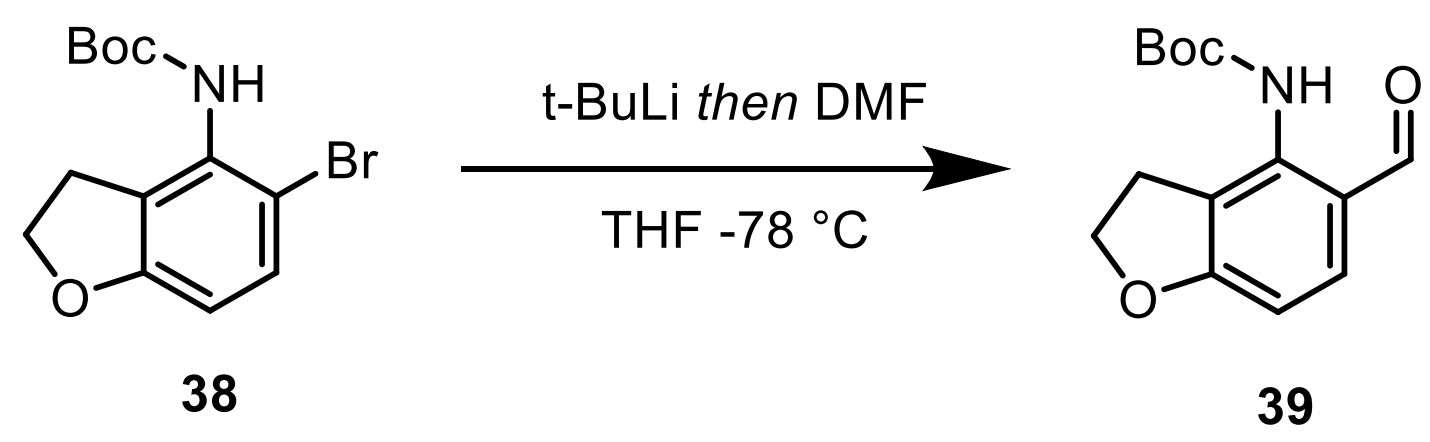

A rigorously dried solution of **38** (9.50 g, 30.4 mmol, 1 eq) in anhydrous THF (152 mL, 0.200M) under Ni was cooled to -78 ℃ before *t*-BuLi 1.7M in pentane (62.0 mL, 106 mmol, 3.5 eq) was added in portions dropwise. After addition, the solution was allowed to slowly warm to -55 ℃ over 30 mins, after which time dry DMF (12.0 mL, 152 mmol, 5 eq) was added dropwise. The reaction was allowed to stir for 1.5 h before it was quenched with 40 mL NH_4Cl at -55 ℃ and then allowed to slowly warm to RT. The mixture was further diluted with EtOAc, water, and 60 mL NH_4Cl and the aqueous phase was extracted 3 x 75 mL EtOAc. The combined organic layers were dried over Na_2SO_4, filtered, and concentrated under reduced pressure. The crude reaction mixture was purified via sodium bisulfite workup (see general procedure D) followed by flash column chromatography on silica (eluent: 20:80 acetone:hexanes) to afford the title compound **39** (4.10 g, 15.4 mmol, 51%) as a white crystalline solid.

TLC: R_f = 0.50 (20:80 EtOAc:Hexanes; UV, CAM)

m.p = 87.3-88.9 ℃

^{1}H NMR (500 MHz, $CDCl_3$): δ 9.88 (s, 1H), 9.72 (s, 1H), 7.43 (d, *J* = 8.3 Hz, 1H), 6.66 (d, *J* = 8.2 Hz, 1H), 4.66 (t, *J* = 8.8 Hz, 2H), 3.35 (t, *J* = 8.8 Hz, 2H), 1.51 (s, 9H).

^{13}C NMR (126 MHz, $CDCl_3$): δ 192.9, 167.5, 152.4, 138.8, 138.3, 120.6, 119.5, 105.5, 80.9 73.0, 30.0, 28.3.

HRMS-ESI (m/z): $[M+Na]^+$ calculated for $[C_{14}H_{17}O_4NNa]^+$: 286.1049, found: 286.1046.

4-Amino-2,3-dihydrobenzofuran-5-carbaldehyde (40):

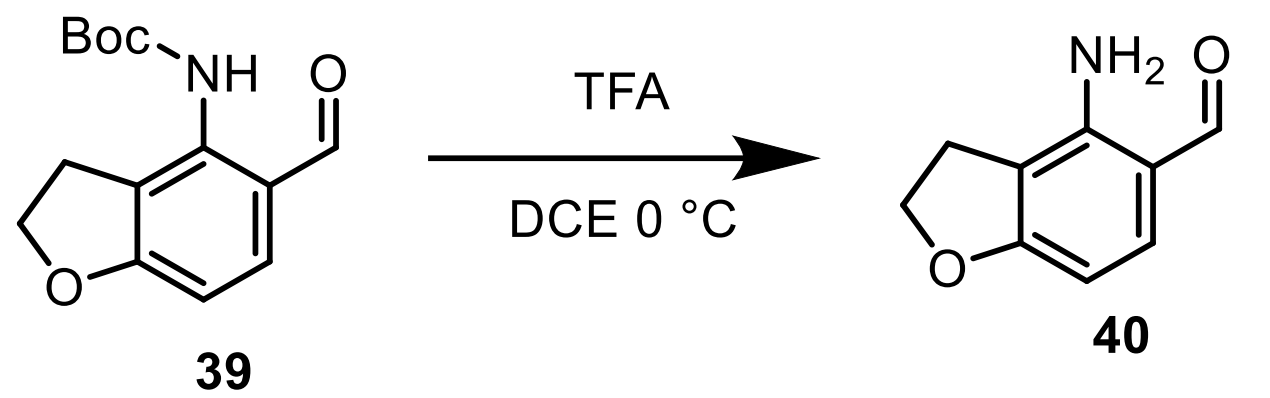

To a solution of **39** (2.60 g, 10.0 mmol, 1 eq) in dry DCE (20.0 mL, 0.500M) at 0 ℃ was added TFA (20.0 mL, 50% v:v) dropwise. The reaction was stirred for 3.5h before it was basified through careful addition of 5M NaOH (~90.0 mL) at 0 ℃. The mixture was extracted with EtOAc 5 x 30 mL and the organic layers were combined, dried over Na_2SO_4, filtered, and concentrated under reduced pressure. The crude product was further purified via silica gel flash column chromatography (eluent: 30:70 EtOAc:hexanes) to afford the title compound **40** (1.10 g, 6.85 mmol, 68%) as a yellow crystalline solid.

TLC: R_f = 0.32 (30:70 EtOAc:Hexanes; UV, CAM)

m.p = 90.6-93.5 ℃

^{1}H NMR (500 MHz, CDCl$_3$): δ 9.69 (s, 1H), 7.31 (d, *J* = 8.4 Hz, 1H), 6.29 (d, *J* = 8.4 Hz, 1H), 6.04 (s, 2H), 4.70 (t, *J* = 8.7 Hz, 2H), 3.00 (t, *J* = 8.8 Hz, 2H).

^{13}C NMR (126 MHz, CDCl$_3$): δ 192.2, 166.2, 147.5, 138.7, 114.9, 109.8, 100.6, 72.5, 26.1.

HRMS-ESI (m/z): $[M+H]^+$ calculated for $[C_9H_{10}O_2N]^+$: 164.0706, found: 164.0701.

4-Bromo-2,3-dihydrobenzofuran-5-carbaldehyde (41):

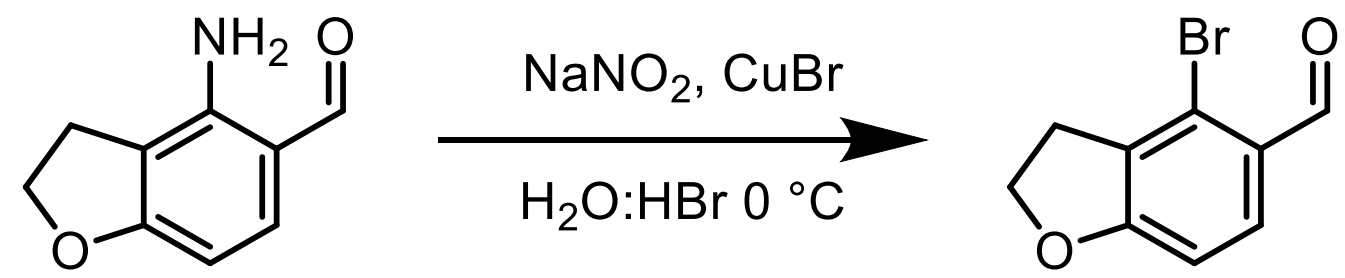

To a cool round-bottom containing **40** (0.150 g, 0.892 mmol, 1 eq) was added a chilled solution of 48% HBr:H_2O (2.20 mL, 0.4M, 1:1.3) and the mixture stirred on ice. The solution was stirred for 3 minutes before a solution of $NaNO_2$ 2M in H_2O (0.450 mL, 0.892 mmol, 1 eq) was added dropwise and stirred for 20 minutes. After 20 minutes, CuBr (0.140 g, 0.982 mmol, 1.1eq) was added portion-wise and the reaction was stirred vigorously and allowed to warm to room temperature overnight. The crude mixture was diluted with EtOAc and water before being quenched with 25 mL $NaHCO_3$. The aqueous layer was extracted with EtOAc 3 x 10 mL and the organic layers were combined, dried over Na_2SO_4, filtered, and concentrated under reduced pressure. The crude product was purified via silica gel flash column chromatography (eluent: 10:90 EtOAc:hexanes) to afford the title compound **41** (0.160 g, 0.721 mmol, 81%) as a white solid.

TLC: R_f = 0.51 (20:80 EtOAc:Hexanes; UV, CAM)

^{1}H NMR (500 MHz, CDCl$_3$): δ 10.17 (s, 1H), 7.79 (d, *J* = 8.3 Hz, 1H), 6.79 (d, *J* = 8.4 Hz, 1H), 4.73 (t, *J* = 8.9 Hz, 2H), 3.29 (t, *J* = 8.9 Hz, 2H).

^{13}C NMR (126 MHz, CDCl$_3$): δ 190.2, 165.3, 132.1, 130.0, 127.1, 124.7, 109.1, 72.1, 30.9.

HRMS-ESI (m/z): $[M+H]^+$ calculated for $[C_9H_8O_2Br]^+$: 226.9702, found: 226.9692.

tert-Butyl ((4-bromo-2,3-dihydrobenzofuran-5-yl)methoxy)carbamate (44):

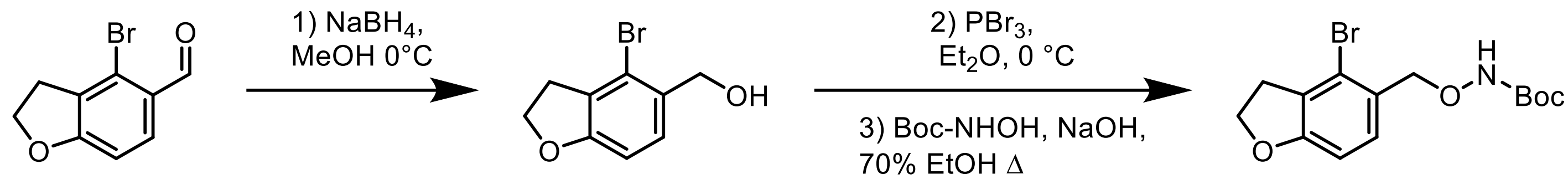

To a stirred solution of **41** (0.910 g, 4.02 mmol, 1 eq) in dry MeOH:THF (27.0 mL, 0.150M, 3:1) at 0 ℃ under Ni was added $NaBH_4$ (0.150 g, 4.02 mmol, 1 eq). After 1 h, the reaction was diluted with EtOAc and 40 mL H_2O, and the aqueous layer extracted 3 x 10 mL EtOAc. The organic layers were combined, dried over Na_2SO_4, filtered, and concentrated under reduced pressure to afford the title compound **42** (0.890 g, 3.90 mmol, 97%) as a white solid which was used in the subsequent reaction without further purification.

TLC: R_f = 0.52 (40:60 EtOAc:Hexanes; UV, CAM)

^{1}H NMR (500 MHz, $CDCl_3$): δ 7.16 (d, *J* = 8.3 Hz, 1H), 6.69 (d, *J* = 8.1 Hz, 1H), 4.65 (s, 2H), 4.61 (t, *J* = 8.8 Hz, 2H), 3.22 (t, *J* = 8.7 Hz, 2H), 1.94 (s, 1H).

^{13}C NMR (126 MHz, $CDCl_3$): δ 160.1, 131.7, 129.6, 129.4, 120.2, 108.3, 71.2, 65.0, 31.8.

To a stirred solution of **42** (0.340 g, 1.50 mmol, 1 eq) in Et_2O (7.50 mL, 0.200M) at 0 ℃ under Ni was added PBr_3 (0.810 g, 2.99 mmol, 2 eq) dropwise. After addition, the reaction mixture was removed from ice and allowed to stir at room temperature for 2 h before it was quenched via dropwise addition of 5 mL $NaHCO_3$. The crude mixture was further diluted with 20 mL $NaHCO_3$ and the resultant aqueous layer extracted 3 x 10 mL EtOAc. The combined organic layers were dried over Na_2SO_4, filtered, and concentrated under

reduced pressure to yield the benzyl bromide which was used as crude in the subsequent reaction.

N-Boc hydroxylamine (0.190 g, 1.40 mmol, 1 eq) was dissolved in 70% EtOH (4.70 mL, 0.300M) before NaOH (0.0560 g, 1.40 mmol, 1 eq) was added, followed by the crude benzyl bromide in 1.50 mL THF added dropwise. After 1 h, the reaction was diluted with EtOAc and quenched with 20 mL NH_4Cl. The aqueous layer was extracted 3 x 10 mL EtOAc and the combined organic layers dried over Na_2SO_4, filtered, and concentrated under reduced pressure. The crude product was purified via silica gel column chromatography (eluent: 20:80 EtOAC:hexanes) to afford the title compound **44** (0.270 g, 0.797 mmol, 53%) as a white solid.

TLC: R_f = 0.58 (30:70 EtOAc:Hexanes; UV, CAM)

^{1}H NMR (500 MHz, $CDCl_3$): δ 7.20 (d, *J* = 8.2 Hz, 1H), 7.08 (s, 1H), 6.70 (d, *J* = 9.2 Hz, 1H), 4.91 (s, 2H), 4.63 (t, *J* = 9.4 Hz, 2H), 3.23 (t, *J* = 8.8 Hz, 2H), 1.47 (s, 9H).

^{13}C NMR (126 MHz, $CDCl_3$): δ 160.6, 156.8, 132.0, 129.4, 126.8, 122.0, 108.1, 81.8, 77.4, 71.2, 31.9, 28.3.

HRMS-ESI (m/z): $[M+Na]^+$ calculated for $[C_{14}H_{18}O_4NBrNa]^+$: 366.0311, found: 366.0299.

tert-Butyl (2-(7,8-dihydrobenzofuro[4,5-c]isoxazol-1(_3H_)-yl)ethyl)carbamate (46):

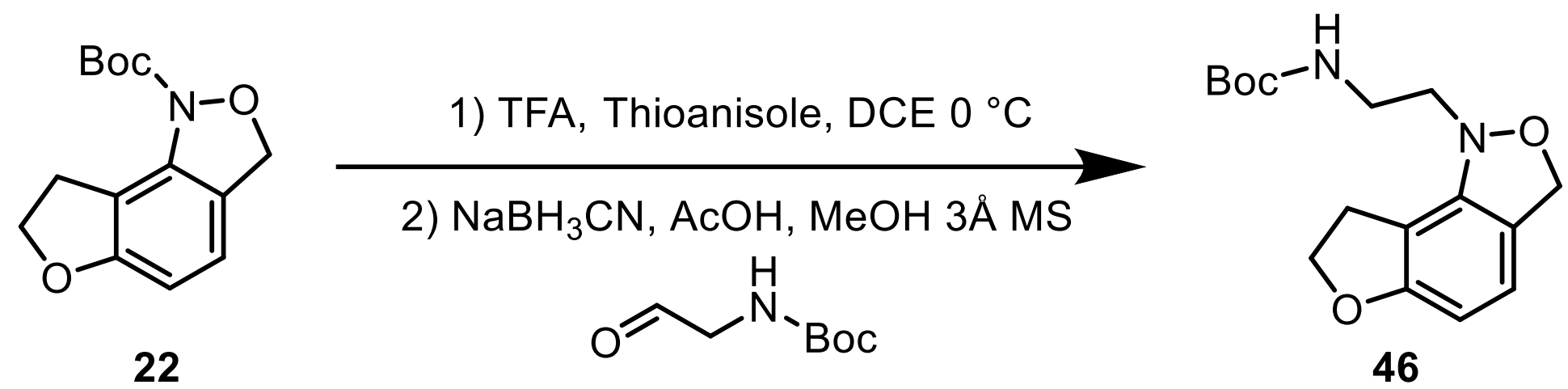

To a stirred solution of **22** (0.0800 g, 0.304 mmol, 1 eq) (see S15) in dry DCE (0.610 mL, 0.500M) was added thioanisole (0.0430 mL, 0.365 mmol, 1.2 eq). The reaction was stirred on ice and, once 0 ℃, TFA (0.610 mL, 50% v:v) was added dropwise. After 30 minutes, the cold reaction mixture was quenched with 20 mL $NaHCO_3$. The aqueous layer was extracted 4 x 3 mL Et_2O and the combined organic layers were dried over Na_2SO_4, filtered, and concentrated under reduced pressure. The crude product was used in the subsequent reaction without further purification.

To a flask containing the crude secondary hydroxylamine was added tert-butyl (2-oxoethyl)carbamate (0.150 g, 0.912 mmol, 3 eq) followed by 3Å MS (0.600 g, 2 g per mmol) and the mixture dissolved in dry MeOH (1.00 mL, 0.300M) under Ni. The mixture was stirred for 15 minutes before AcOH (0. 0520 mL, 0.912 mmol, 3 eq) followed by $NaBH_3CN$ (0.0570 g, 0.912 mmol, 3 eq) was added. After 7 h, the reaction was quenched with 20 mL $NaHCO_3$ and the aqueous layer extracted 4 x 5 mL EtOAc. The organic layers were combined, dried over Na_2SO_3, filtered, and concentrated under reduced pressure. The crude product was purified via silica gel column chromatography (eluent: 25:75 EtOAC:hexanes) and the corresponding aldehyde byproduct removed via bisulfite workup (see general procedure D) to afford the title compound **46** (0.0390 g, 0.127 mmol, 42%) as a white solid.

TLC: R_f = 0.48 (40:60 EtOAc:Hexanes; UV, CAM)

^{1}H NMR (500 MHz, CDCl$_3$): δ 6.88 (d, *J* = 8.0 Hz, 1H), 6.48 (d, *J* = 9.2 Hz, 1H), 5.13 (s, 3H), 4.58 (t, *J* = 8.6 Hz, 2H), 3.44 (d, *J* = 5.7 Hz, 2H), 3.32 (s, 2H), 3.11 (t, *J* = 8.6 Hz, 2H), 1.44 (s, 9H).

^{13}C NMR (126 MHz, CDCl$_3$): δ 161.6, 156.1, 146.0, 121.8, 120.7, 109.6, 104.6, 79.3, 71.6, 70.8, 56.0, 38.9, 28.5, 27.4.

HRMS-ESI (m/z): $[M+Na]^+$ calculated for $[C_{16}H_{22}O_4N_2Na]^+$: 329.1472, found: 329.1468.

***N*-(2-(7,8-Dihydrobenzofuro[4,5-*c*]isoxazol-1(*3H*)-yl)ethyl)propionamide (2):**

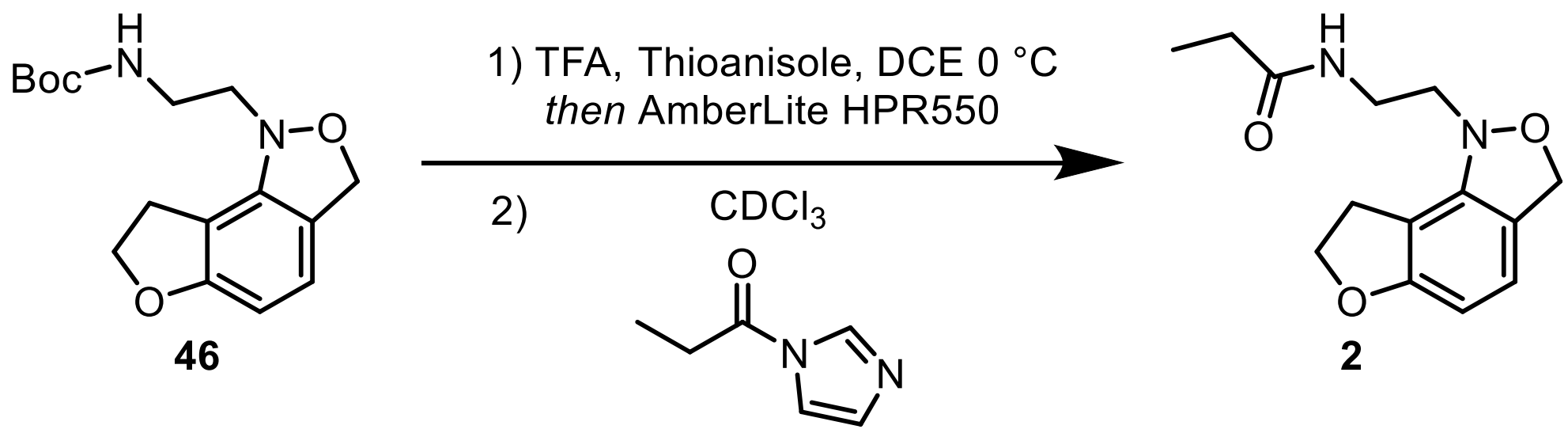

Compound **46** (0.011 g, 0.036 mmol, 1 eq) was dissolved in anhydrous DCE (0.073 mL, 0.50M) followed by the addition of thioanisole (0.0054 mL, 0.044 mmol, 1.2 eq). The reaction was stirred on ice until 0 ℃ before TFA (0.073 mL, 50% v:v) was added dropwise and the mixture stirred for 1 h. The mixture was subsequently concentrated under reduced pressure and the resultant TFA salt dissolved in 0.20 mL dry MeOH. A small scoop of AmberLite HPR550 OH Anionic Exchange Resin was added and the reaction stirred until the pH reached 7. Once neutral, the resin was removed by cotton filtration, and the resulting solution was concentrated under reduced pressure to yield the primary amine. The amine was dissolved in $CDCl_3$ (0.38 mL, 0.10M) before a solution of previously prepared propionyl imidazolide (0.0045 g, 0.036 mmol, 1 eq) in 0.20 mL $CDCl_3$ was added dropwise. The reaction was monitored with ^{1}H-NMR until full consumption of the p amine was observed (~1.5 h), after which time the crude reaction mixture was concentrated directly onto celite and subjected to flash column chromatography on neutral alumina (eluent: 80:20 EtOAC:hexanes) to afford the title compound **2** (0.0061 g, 0.023 mmol, 64%) as a slightly yellow solid.

Preparation of propionyl imidazolide:

Propionic acid (0.10 g, 1.4 mmol, 1 eq) was dissolved in DCE (4.5 mL, 0.30M) and stirred on ice. Once 0 ℃, carbonyldiimidazole (0.33 g, 2.0 mmol, 1.5 eq) was added portion wise and the reaction stirred overnight. The crude mixture was washed 3 x 1 mL H_2O, 1 x 1 mL brine, and the organic layer dried over Na_2SO_4, filtered, and concentrated under reduced pressure to afford the propionyl imidazolide (0.17 g, 1.4 mmol) as a white solid which was used directly without further purification.

TLC: R_f = 0.37 (80:20 EtOAc:Hexanes; UV, CAM)

^{1}H NMR (500 MHz, $CDCl_3$): δ 6.89 (d, *J* = 7.9 Hz, 1H), 6.49 (d, *J* = 8.0 Hz, 1H), 6.13 (s, 1H), 5.14 (s, 2H), 4.58 (t, *J* = 8.7 Hz, 2H), 3.62 – 3.54 (m, 2H), 3.34 (t, *J* = 4.9 Hz, 2H), 3.10 (t, *J* = 8.6 Hz, 2H), 2.22 (q, *J* = 7.6 Hz, 2H), 1.16 (t, *J* = 6.9 Hz, 3H).

^{13}C NMR (126 MHz, $CDCl_3$): δ 174.0, 161.7, 145.9, 121.6, 120.8, 109.7, 104.8, 71.6, 70.8, 55.6, 37.8, 29.8, 27.4, 9.9.

HRMS-ESI (m/z): $[M+H]^+$ calculated for $[C_{14}H_{19}O_3N_2]^+$: 263.1390, found: 263,1377.

Biochemical Data

In vitro ADME was conducted through subcontract with Pharmaron. Agonist functional data was generously provided by the National Institute of Mental Health's Psychoactive Drug Screening Program, Contract # 75N95023C00021 (NIMH PDSP). The NIMH PDSP is Directed by Bryan L. Roth MD, PhD at the University of North Carolina at Chapel Hill and Project Officer Jamie Driscoll at NIMH, Bethesda MD, USA.

LogP

Determined following the LC-UV method with progesterone as control.[4]

$LogD_{7.4}$

Determined following the shake-flask method with nicardipine as control.[4]

Aqueous Solubility

Determined as kinetic solubility in phosphate puffer at pH 7.4 with progesterone and diclofenac as controls.[4]

MDCKII-MDR1 Permeability

MDCKII-MDR1 cells were obtained from the Netherlands Cancer Instituted (Amsterdam). Hepes, Penicillin, Streptomycin, and DMSO were purchased through Solarbio while Hank's balanced salt solution (HBSS), GlutaMAXTM-I, Trypsin/EDTA and Minimum Essential Medium Eagle (MEM) were purchased from Gibco. Fetal bovine serum was purchased from AusGeneX. HTS Transwell-96 Well (Cat. No. 3391) Permeable Supports were purchased from Corning and Millicell Epithelial Volt-Ohm measuring system was purchased from MilliporeSigma. Cellometer Vision was purchased from Nexcelom Bioscience and Infinite 200 PRO microplate reader is purchased from Tecan. MTS2/4 orbital shaker was purchased from IKA.

Table S1. MDCKII-MDR1 permeability results of test compounds and controls.

Compound	$P_{app(A-B)}$(10-6, cm/s)	$P_{app(B-A)}$(10-6, cm/s)	Efflux Ratio	Recovery (%) A-B	Recovery (%) B-A
Metoprolol	29.61	24.35	0.82	104.50	97.02
Digoxin	0.54	10.90	20.21	99.32	109.90
2	40.96	35.15	0.86	111.48	100.84
1	35.72	27.95	0.78	96.22	92.88

[a.] P_{app} = apparent permeability.

Table S2. Assessment of MDCKII-MDR1 cell monolayer integrity.

Compound	TEER A-B (Ω × cm^2)	TEER B-A (Ω × cm^2)	LY Leakage A-B (%)	LY Leakage B-A (%)
Metoprolol	76.51	84.44	0.31	0.31
Digoxin	80.87	84.44	0.29	0.35
2	81.37	81.08	0.31	0.28
1	73.14	82.23	0.29	0.31

[a.] TEER = Transepithelial electrical resistance. LY = Lucifer Yellow.

Caco-2 Permeability

Caco-2 cells were obtained from the American type culture collection (ATCC) (#HTB-37). Trypsin/EDTA were purchased from Solarbio and Non-essential amino acids (NEAA) were purchased from Gibco. Dulbecco's Modified Eagle's Medium (DMEM) is purchased from HyClone Corporation. All other materials obtained as stated previously.

Table S3. Caco-2 permeability results of test compounds and controls.

Compound	$P_{app(A\text{-}B)}$(10-6, cm/s)	$P_{app(B\text{-}A)}$(10-6, cm/s)	Efflux Ratio	Recovery (%) AP-BL	Recovery (%) BL-AP
Metoprolol	28.56	26.03	0.91	110.36	98.52
Digoxin	0.49	18.83	38.71	119.41	92.55
2	34.52	24.74	0.72	103.65	93.05
1	29.04	22.11	0.76	90.29	87.94

[a.] P_{app} = apparent permeability. AP = apical. BL = basolateral.

Table S4. Assessment of Caco-2 cell monolayer integrity.

Compound	TEER A-B ($\Omega \times cm^2$)	TEER B-A ($\Omega \times cm^2$)	LY Leakage A-B (%)	LY Leakage B-A (%)
Metoprolol	502.36	531.75	0.11	0.12
Digoxin	502.22	505.36	0.13	0.26
2	452.67	538.68	0.16	0.11
1	503.79	558.20	0.12	0.15

[a.] TEER = Transepithelial electrical resistance. LY = Lucifer Yellow.

Metabolic Stability in Hepatocytes

Mixed gender human hepatocytes were obtained from BioVT (#X008005) and male Sprague-Dawley rat hepatocytes were obtained from TPCS (#CRH2-100SD-SQ). Williams'E Medium, DPBS, GlutaMAX (100x), HEPES, and Human-insulin were purchased from Gibco. FBS and Dexamethasone were purchased from MilliporeSigma while Isotonic percoll was obtained from Cytvia.

Table S5. Metabolic stability in rat and human hepatocytes.

Compound	Species	*In vitro t1/2 (min)*	*In vitro* CL_{int} (μL/min/106 cells)	Scale-up CL_{int} (μL/min/106 cells)	Predicted Hepatic CL_H (mL/min/kg)	Hepatic Extraction Ratio (ER)
Verapamil	Human	28.37	48.86	124.32	17.97	0.86
	Rat	6.78	204.41	956.64	63.49	0.93
2	Human	189.75	7.31	18.59	9.86	0.47
	Rat	60.45	23.03	107.77	41.65	0.61
1	Human	17.97	77.21	196.45	18.97	0.90
	Rat	13.88	99.85	467.28	59.36	0.87

[a] CL = Clearance.

Table S6. Remaining percentage of test compounds in human and rat hepatocytes.

Compound Species		Remaining Percentage (%)					
		0.5 min	15 min	30 min	60 min	90 min	120 min
Verapamil	Human	100.00	66.26	44.54	20.93	11.26	6.42
	Rat	100.00	22.72	6.16	0.65	0.15	0.04
2	Human	100.00	98.45	93.15	82.02	70.94	67.30
	Rat	100.00	78.04	67.11	47.11	36.19	23.80
1	Human	100.00	55.62	32.03	9.70	2.53	0.98
	Rat	100.00	47.46	22.93	4.22	0.72	0.10

MT1/MT2 Agonist Functional Data

“HTLA cells are transfected with GPCR tango constructs overnight (see calcium mobilization section for detailed transfection protocol) and are plated in DMEM supplemented with 1% dialyzed FBS in Poly-L-Lys (PLL)-coated 384-well white clear bottom cell culture plates at a density of 15,000 cells in a volume of 40 μl per well. Cells are incubated for at least 6 hours (or overnight) to allow them to recover before receiving drug stimulation. Drug stimulation solutions are prepared in sterile-filtered Tango assay buffer at 5x concentration and added to cells (10 μl per well) overnight. To measure antagonist activity, drug solutions are made at 6x of the final concentration and are preincubated with cells for 30 min before addition of 10 μl of a final EC80 concentration of reference agonist. The EC80 concentration is determined in separate preliminary dose-response assays. On the day of measurement, medium and drug solutions are removed and 20 μl per well of BrightGlo reagent (diluted 20-fold with Tango assay buffer) are added. Plates are incubated for 20 min at room temperature in the dark before being counted on a luminescence counter.”[5]

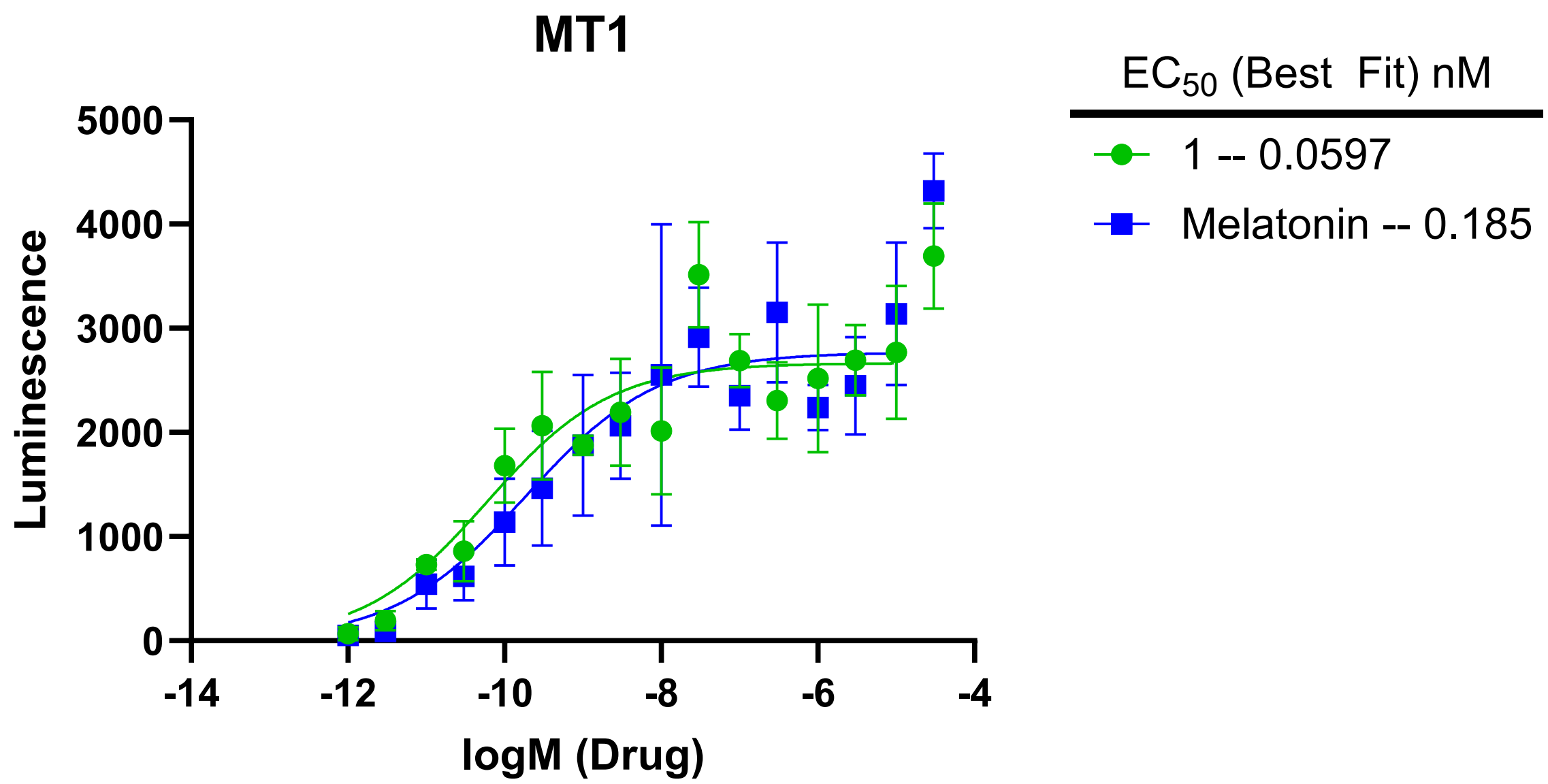

log(agonist) vs. response – Variable slope (four parameters)	1	Melatonin	Global
**Best-fit values**			
**Bottom**	0.0007905	0.0007905	0.0007905
**Top**	2666	2770	
**LogEC50**	-10.22	-9.732	
**Hill Slope**	0.5480	0.5155	
**EC50**	5.968e-011	1.853e-010	
**Span**	2666	2770	
**95% CI (profile likelihood)**			
**Bottom**	??? to 336.8	??? to 336.8	??? to 336.8
**Top**	239 to 2972	2475 to 3097	
**LogEC50**	-10.55 to -9.632	-10.05 to -9.156	
**Hill Slope**	0.3620 to 1.128	0.3611 to 0.9714	
**EC50**	2.815e-011 to 2.336e-010	8.811e-011 to 6.984e-010	
**Goodness of Fit**			
**Degrees of Freedom**			113
**R squared**	0.7762	0.7593	0.7674
**Sum of Squares**	14334715	18869044	33203759
**Sy.x**			542.1
**Constraints**			
**Bottom**	Bottom > 0 and shared	Bottom > 0 and shared	
**Number of points**			
**# of X values**	60	60	
**# Y values analyzed**	60	60	

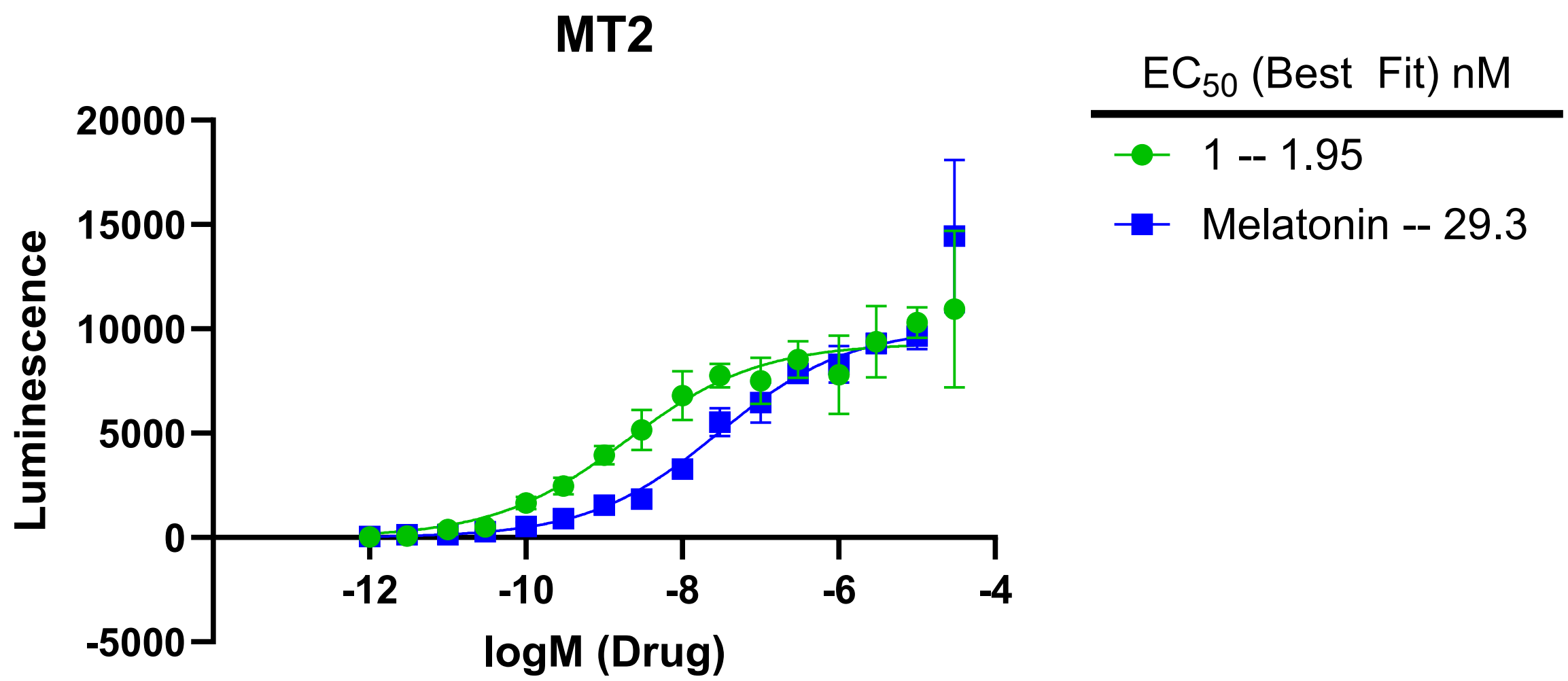

log(agonist) vs. response – Variable slope (four parameters)	1	Melatonin	Global
**Best-fit values**			
**Bottom**	0.0002454	0.0002454	0.0002454
**Top**	9317	10042	
**LogEC50**	-8.709	-7.533	
**Hill Slope**	0.5135	0.5220	
**EC50**	1.953e-009	2.932e-008	
**Span**	9317	10042	
**95% CI (profile likelihood)**			
**Bottom**	??? to 278.7	??? to 278.7	??? to 278.7
**Top**	8759 to 9920	9168 to 11027	
**LogEC50**	-8.893 to -8.468	-7.748 to -7.251	
**Hill Slope**	0.4312 to 0.6563	0.4365 to 0.6916	
**EC50**	1.279e-009 to 3.405e-009	1.787e-008 to 5.607e-008	
**Goodness of Fit**			
**Degrees of Freedom**			113
**R squared**	0.9374	0.9827	0.9605
**Sum of Squares**	50732035	13533602	64265636
**Sy.x**			754.1
**Constraints**			
**Bottom**	Bottom > 0 and shared	Bottom > 0 and shared	
**Number of points**			
**# of X values**	60	60	
**# Y values analyzed**	60	60	

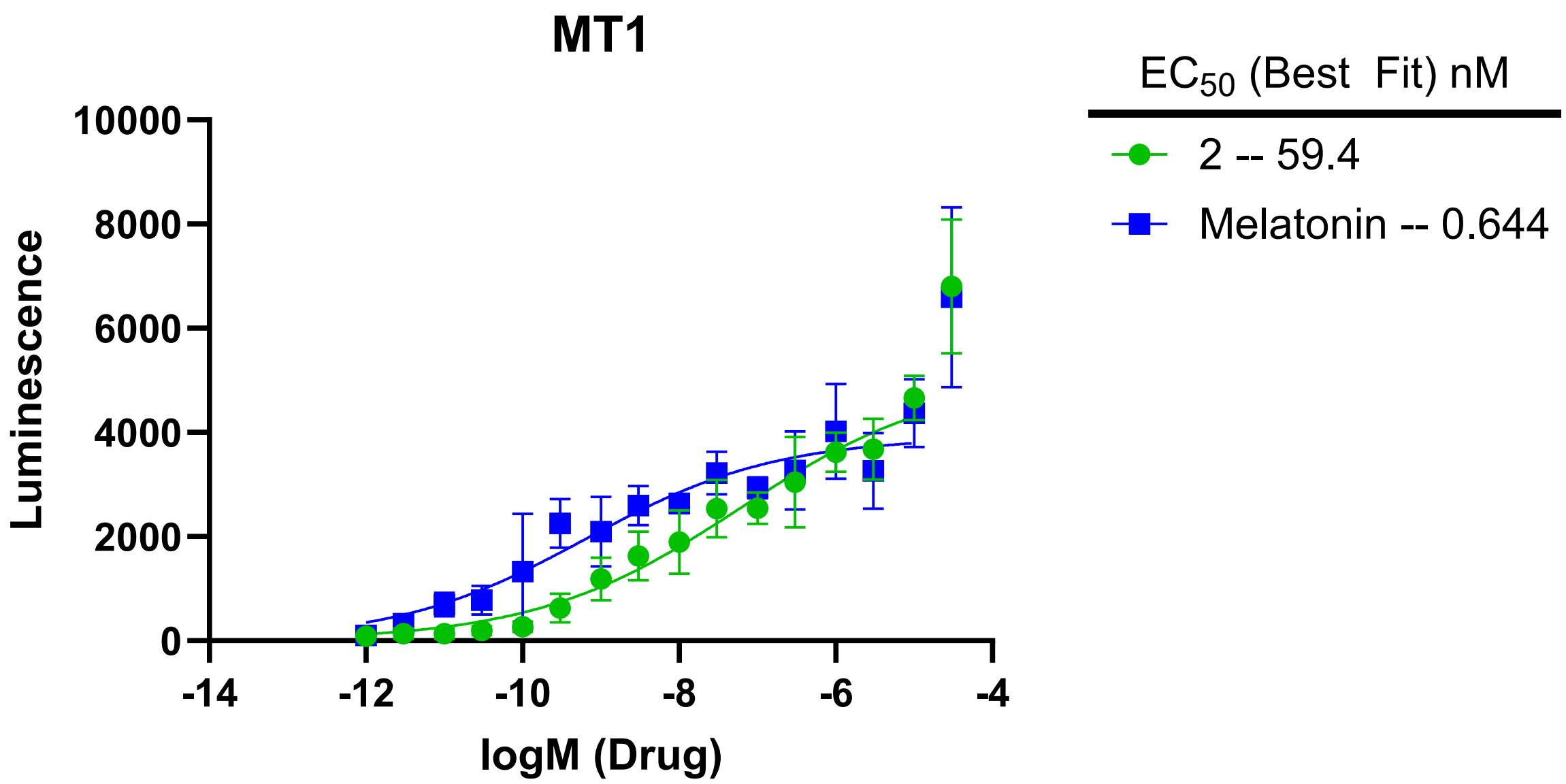

log(agonist) vs. response – Variable slope (four parameters)	2	Melatonin	Global
Best-fit values			
Bottom	~ 4.930e-032	~ 4.930e-032	~ 4.930e-032
Top	5078	3913	
LogEC50	-7.226	-9.191	
Hill Slope	0.3335	0.3585	
EC50	5.939e-008	6.437e-010	
Span	~ 5078	~ 3913	
95% CI (profile likelihood)			
Bottom			
Top	4137 to 7619	3509 to 4597	
LogEC50	-7.884 to -5.775	-9.643 to -8.497	
Hill Slope	0.2386 to 0.4559	0.2551 to 0.4982	
EC50	1.306e-008 to 1.678e-006	2.277e-010 to 3.187e-009	
Goodness of Fit			
Degrees of Freedom			113
R squared	0.9242	0.8251	0.8830
Sum of Squares	10568748	20100700	30669448
Sy.x			521.0
Constraints			
Bottom	Bottom > 0 and shared	Bottom > 0 and shared	
Number of points			
# of X values	60	60	
# Y values analyzed	60	60	

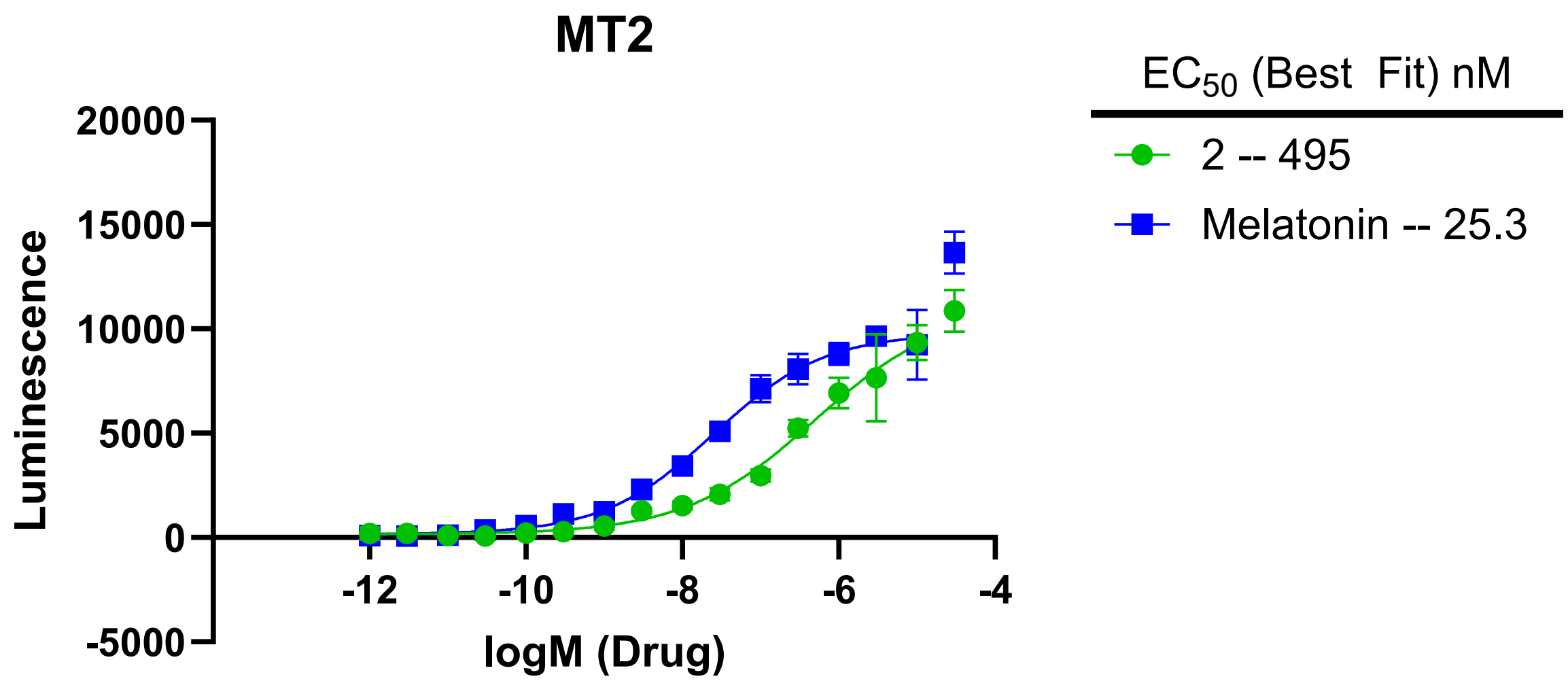

log(agonist) vs. response – Variable slope (four parameters)	2	Melatonin	Global
**Best-fit values**			
**Bottom**	151.7	151.7	151.7
**Top**	11102	9796	
**LogEC50**	-6.305	-7.597	
**Hill Slope**	0.5237	0.6140	
**EC50**	4.952e-007	2.531e-008	
**Span**	10950	9645	
**95% CI (profile likelihood)**			
**Bottom**	-infinity to 348.5	-infinity to 348.5	-infinity to 348.5
**Top**	9635 to 13916	9304 to 10390	
**LogEC50**	-6.592 to -5.810	-7.731 to -7.448	
**Hill Slope**	0.4096 to 0.6649	0.5170 to 0.7314	
**EC50**	2.557e-007 to 1.549e-006	1.856e-008 to 3.562e-008	
**Goodness of Fit**			
**Degrees of Freedom**			113
**R squared**	0.9620	0.9801	0.9735
**Sum of Squares**	21911893	16123431	38035323
**Sy.x**			580.2
**Constraints**			
**Bottom**	Bottom > 0 and shared	Bottom > 0 and shared	
**Number of points**			
**# of X values**	60	60	
**# Y values analyzed**	60	60	

Figure S3. UHPLC Trace of 2.

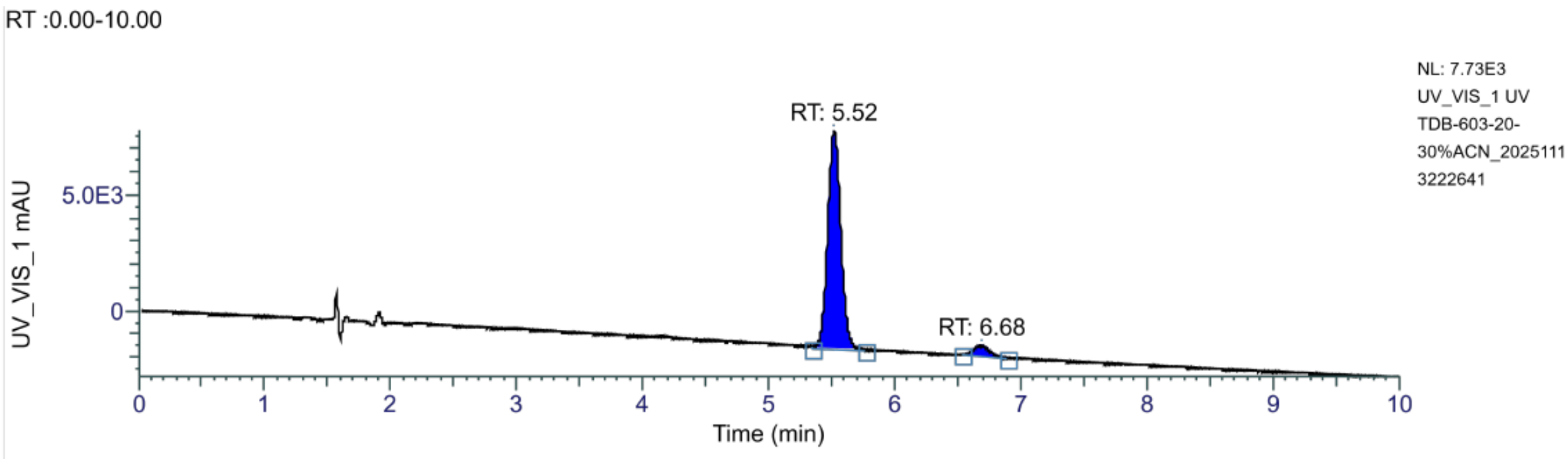

Index	RT (Min)	RT (Sec)	Start RT	End RT	Peak Area	Peak Height	Baseline W...	TraceId	% Area	% Height
1	5.52	331.2	5.34	5.77	6.792E+4	9.377E+3	0.43	Trace1	94.39	95.3
2	6.68	401	6.54	6.9	4.039E+3	4.629E+2	0.36	Trace1	5.61	4.7

UHPLC trace of hydroxalog **2** using automated Avalon peak area algorithm. Mobile Phase A: CH_3CN. Mobile phase B: 0.1 % (v/v) formic acid in ultrapure H2O. Flow rate: 1.000 [mL·min-1], injection volume: 0.30 μL. Mobile Phase A: 30% Mobile Phase B: 70%.

Computational Workflow and Discussion:

The initial conformational analysis began by performing CREST-based conformer searches with optimization at the AimNet2 (ωB97M-D3) level of theory using Rowan.[6-11] This revealed the unusual gauche conformation, as described in the text, and prompted further investigation using more rigorous computational methods. All calculations and modeling from this point were conducted using the Sapelo2 HPC at the University of Georgia.

The GOAT conformer search program in Orca 6.1.1 with the semi-empirical tight binding method (xTB) with analytical linearized Poisson Boltzmann (ALPB)[12] implicit water solvation was used to generate initial global minima.[12-17] Both **1** and **2** converged to generate global minima structures which underwent geometry optimization at the PBE0-D3BJ/def2-TZVP level of theory with RIJCOSX approximation and CPCM implicit water solvation, as verified by vibrational frequency calculations, generating structures **48** and **49** respectively.[18-23]

Figure S4. Expanded Computed Ground State Conformations of 1 and 2.

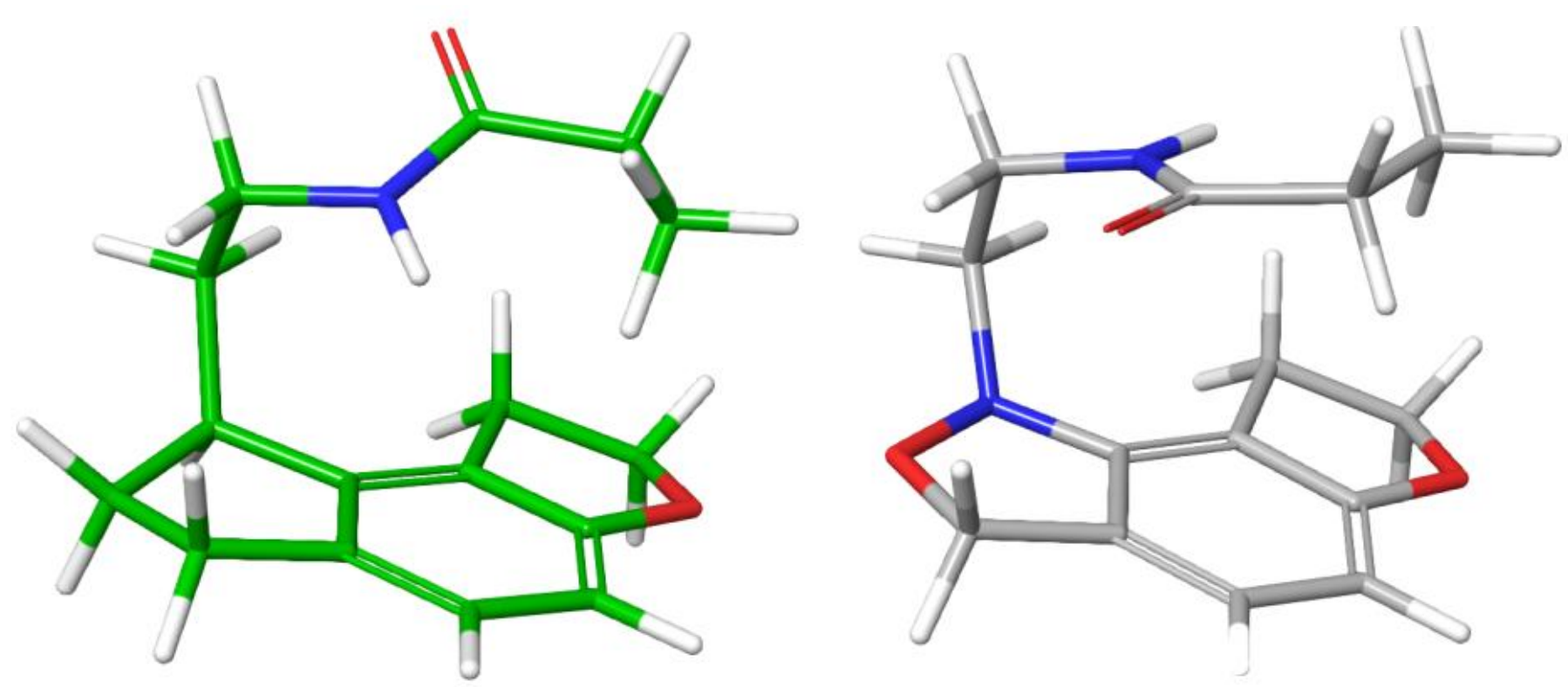

48 **49**

The PBE0-D3BJ/def2-TZVP level of theory has been used in several similar studies assessing ligand conformational energy,[24,25] including one specifically examining the conformations of melatonin that showed good performance using PBE0 with D3BJ dispersion.[26] Additionally, a robust method was desired to ensure accurate handling of the pyramidal nitrogen geometry of **2**, which is central to our study. The standard practice

primary basis set Def2-TZVP was paired with def2/J and RIJCOSX to reduce computation time with minimal loss in accuracy, while TightSCF was selected to ensure the most precise convergence. This level of theory was employed for all subsequent calculations.

Figure S5. Geometry Optimization Inputs

!PBE0 D3BJ def2-TZVP def2/J RIJCOSX TightSCF Opt Freq CPCM(water)

In order to rule out the possibility of a degenerate anti-conformation or the calculation being generally 'stuck' in a local energy minimum, the ground state **48** was converted to the corresponding anti-conformation via a 20-step 'dihedral scan'. In this fashion, the sidechain was rotated from its starting dihedral angle of 71.4° to 180° in 20 steps where each step involved a local geometry optimization at the same level of theory. The scan revealed that the sidechain passes through a high energy ($\Delta E = 4.5$ kcal mol^{-1}) eclipsing conformation before it reaches the anti-conformer which is 2.1 kcal mol^{-1} higher in energy than the starting gauche conformation, further supporting the gauche conformation as the likely ground state.

Structure **48** was then used as a model to evaluate various ligand strand conformational energy (LSCE) workflows. First, the ligand was extracted directly from the protein-ligand complex (PDB 6ME2)[27] and a single-point energy calculation was performed at the same level of theory in the gas phase. This resulted in an unrealistic initial energy difference of over 33 kcal mol^{-1} . Adding hydrogens to the ligand structure using OpenBabel followed by a local optimization of only the hydrogens atoms at the same level of theory resulted in a structure with a slightly improved but still unrealistic energy difference of 26.8 kcal mol^{-1}. Closer examination of the structure of **55** in complex with MT1 (PDB #6ME2) revealed the side chain to be in the cis amide configuration and to have an eclipsed conformation of the ethylidene group, as depicted in Figure S7. Based on the dihedral scan, the eclipsed conformation has a $\Delta E = 4.5$ kcal mol^{-1} while cis amides are typically

Figure S6. Expanded Dihedral Scan and Input

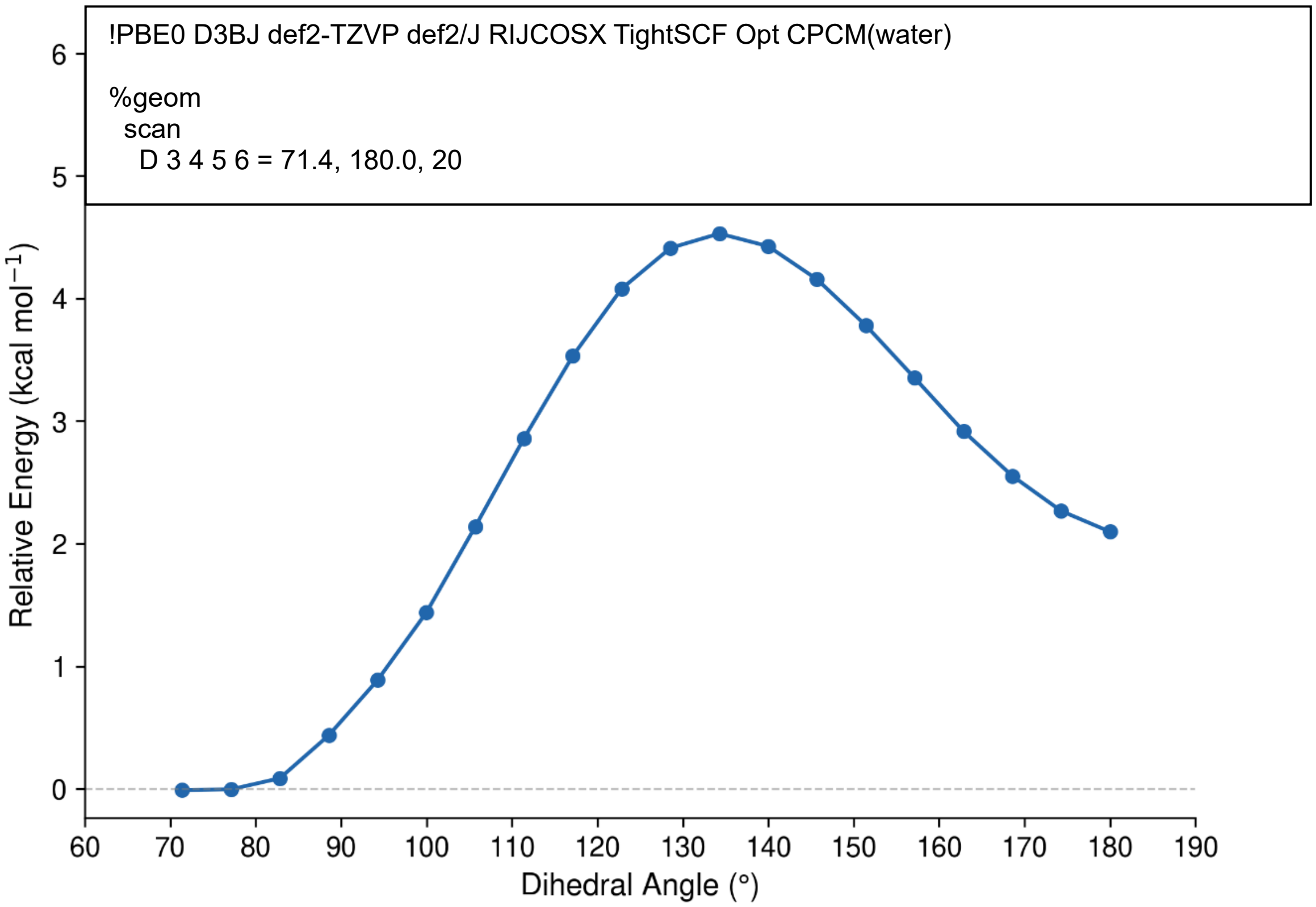

2-3 kcal mol^{-1} higher[28] in energy than their trans-isomers, overall suggesting the bound ligand conformation to be erroneous.

Docking

While crystal structures of both MT1 and MT2 are available, that of MT1 was selected going forward as **1** and **2** showed the greatest difference in affinity for MT1. Also, the active sites of MT1 and MT2 are conserved.

Figure S7. Expanded Structures of Dihedral Scan, Bound Ligand, and Crystal

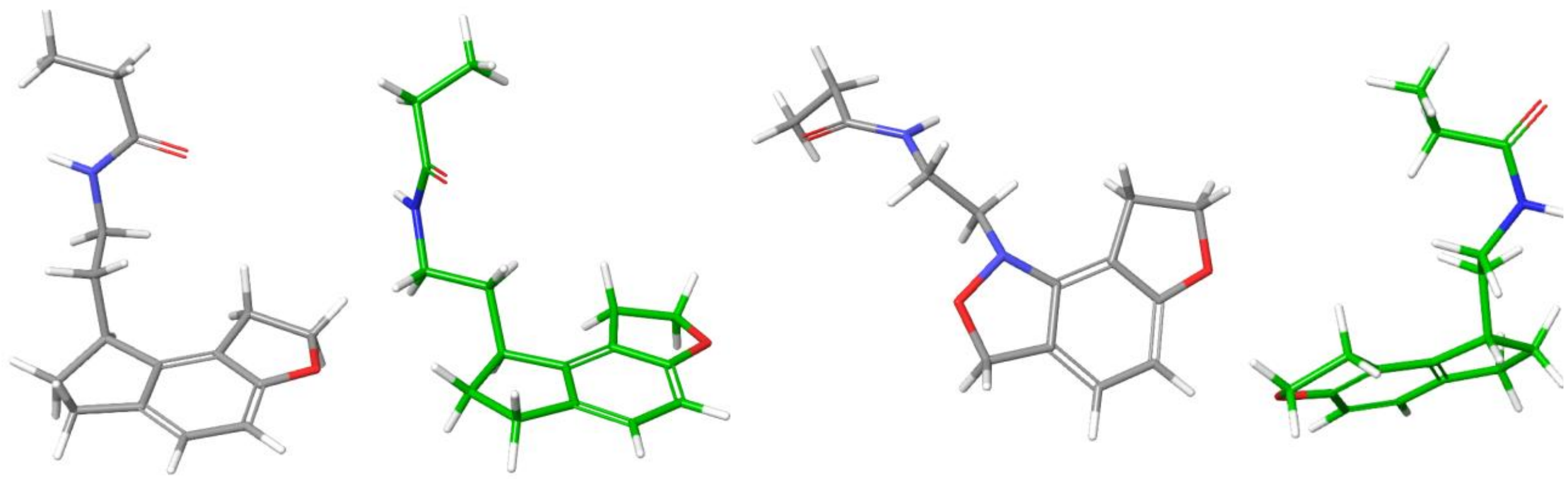

52 53 54 55

Furthermore, due to the high flexibility of GPCRs such as MT1 induced fit docking, which allows for active site and protein structure relaxation after docking, was selected. In preparation for docking, 6ME2 was first optimized using the default protein preparation sequence in Maestro. Following that, **48** and **49** were subjected to LigPrep using OPLS4 at pH 7.4 with all chiralities retained. It should be noted that LigPrep was ultimately inconsequential as the structures are already in an optimized form and they should not be ionized at physiological pH. Next, using the Glide Induced Fit Docking program, the docking site was defined by the JEV ligand (Ramelteon) of 6ME2 with an inner box of 10Å. Finally, prime refinement was done using OPLS_2005 and redocking constrained to a 5Å inner box with standard precision, ultimately furnishing the docked structures **53** and **54**.[29-33]

Calculating the single point energies of **53** and **5**4 at the same level of theory gave ∆E values of 9.17 kcal mol^{-1} for Ramelteon **53** and a value of 9.65 kcal mol^{-1} for hydroxalog **54**. While the ∆Es of **53** and **54** could be considered individually high, it is the relative difference between them that is most relevant. Further assessment of the energy values was done by performing additional scanning of ground state **48**. In this instance, the side chain of ‘dihedral scan’ conformer **51** was further rotated by additionally constraining the dihedral angle of C14-C10-C9-C8 to -133° so that the sidechain adopted a conformation more closely resembling that of the crystal, generating the conformer **52** which was 2.1 kcal mol^{-1} higher in energy. Next, using the neural network based StrainRelief[34] tool for

determining LCSE generated a value of 18.0 kcal mol^{-1} for Ramelteon **1** and thus, in this context, the previously computed values of 9.17 and 9.65 kcal mol^{-1} seem reasonable.

Table S7. Summary of Energy Differences from Ground State Structures

Method	Description	Energy Difference (kcal mol^{-1})
DFT (Gas)	Ligand Extracted from PDB (**55**)	33.3 (from **48**)
DFT (Gas)	PDB Ligand and Hydrogen Atom Optimization	26.8 (from **48)**
DFT (Water)	Dihedral Scan (**48→51**)	2.1 (from **48**)
DFT (Water)	Double Dihedral Constraints (**52**)	2.1 (from **48**)
StrainRelief (NNP)	Ligand Extracted from PDB	18.0 (using **55**)
DFT (Water)	Induced Fit Docking Pose of **48** (**53**)	9.17 (from **48**)
DFT (Water)	Induced Fit Docking Pose of **49** (**54**)	9.65 (from **49**)

Following docking, **53** and **54** were subject to a 100 ns MD simulation in the membrane embedded structure of 6ME2 with an orthorhombic box of SPC water with a 10Å buffer at a constant temperature and pressure with OPLS4 forcefield using Desmond in Schrodinger. Preforming trajectory cluster analysis furnished the structures **55** and **56** from the top-ranking clusters, illustrating the gauche preference of the hydroxalog.

Figure S8. Representative Structures of the Top Clusters from MD of 53 and 54

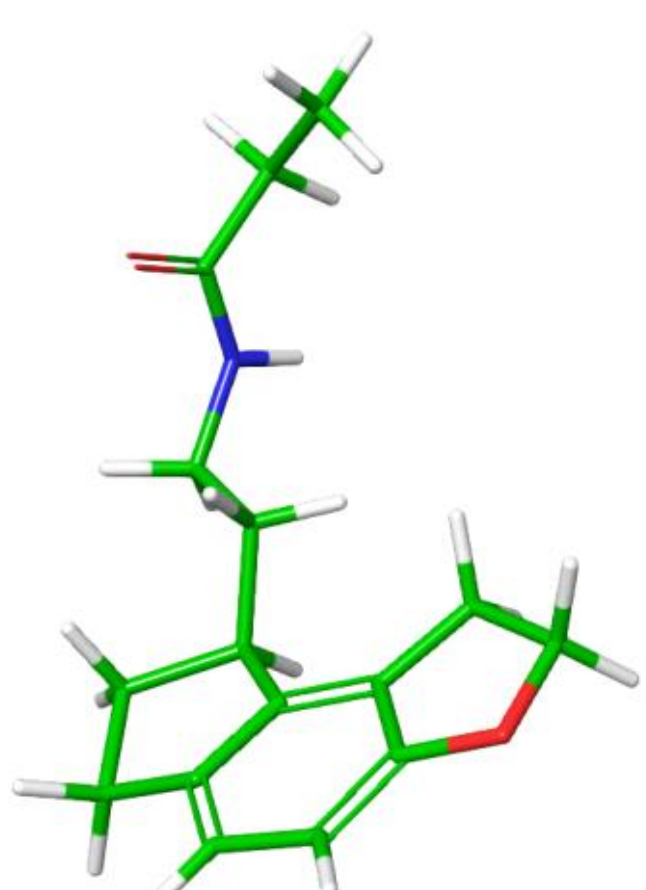

55

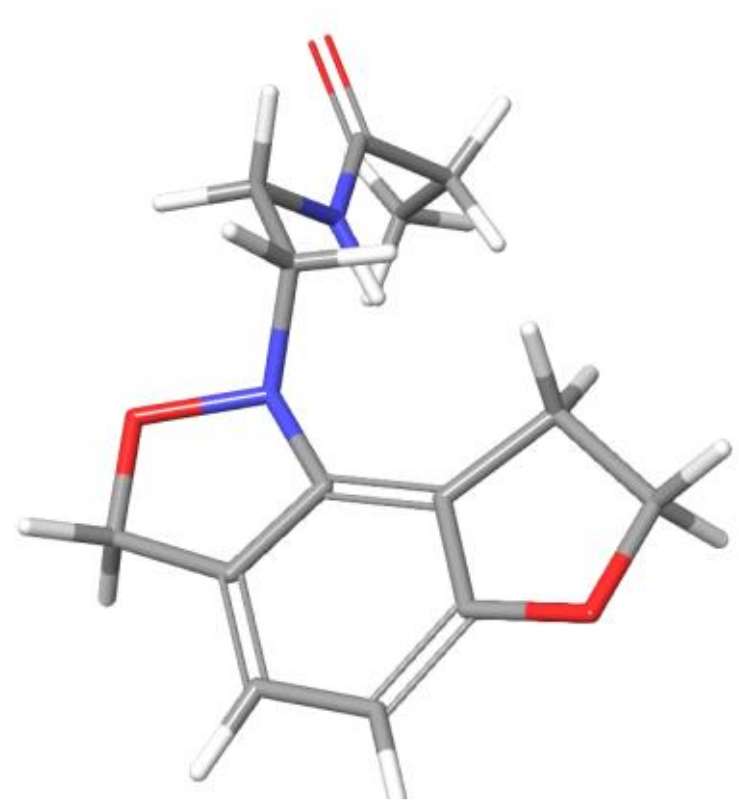

56

*XYZ Coordinates – Ramelteon Ground State Conformation (**48**):*

C	0.158911	2.03529	0.616768
C	0.400655	1.041746	1.738858
C	-0.28114	-0.30384	1.640816
N	-1.34764	-0.38839	0.834525
C	-2.17839	-1.57142	0.760498
C	-3.14768	-1.72587	1.926723
C	-4.32997	-0.75126	1.964575
C	-5.24787	-0.81906	0.723167
C	-4.92264	0.408864	-0.14488
C	-4.25989	1.35666	0.816228
C	-3.92759	0.698654	2.003748
C	-3.27513	1.39173	3.003838
C	-2.72217	0.979987	4.335989
C	-2.36302	2.339322	4.944872
O	-2.2978	3.282949	3.851418
C	-2.96194	2.730446	2.802006
C	-3.27871	3.405392	1.635385
C	-3.93597	2.693247	0.632081
O	0.127279	-1.26354	2.296601
H	-0.88606	2.350757	0.565607
H	0.442031	1.61988	-0.35312
H	0.754819	2.934638	0.782071
H	0.076058	1.468404	2.695209
H	1.466901	0.831454	1.848256
H	-1.67867	0.451642	0.390793
H	-1.51827	-2.44021	0.733412
H	-2.7137	-1.54089	-0.19044
H	-2.58085	-1.65002	2.858672
H	-3.55352	-2.74206	1.889686
H	-4.90092	-1.00058	2.865304
H	-6.28952	-0.76604	1.047328

H	-5.12734	-1.75857	0.179793
H	-5.8157	0.835162	-0.60842
H	-4.23851	0.153992	-0.96241
H	-1.83914	0.345265	4.210619
H	-3.43634	0.433144	4.953808
H	-1.39955	2.354066	5.451898
H	-3.13952	2.682535	5.634439
H	-3.02042	4.45002	1.508982
H	-4.19442	3.196466	-0.29395

*XYZ Coordinates – Hydroxalog Ground State Conformation (**49**):*

C	2.676763	-1.04249	3.883765
C	1.948406	-1.78976	2.782305
C	0.673103	-2.51745	3.140922
N	0.228582	-2.37579	4.398996
C	-1.0235	-2.90518	4.87778
C	-1.95286	-1.83035	5.422295
N	-2.51853	-0.90166	4.456026
O	-3.29159	-1.62965	3.490428
C	-2.81103	-1.35691	2.163994
C	-1.82213	-0.25626	2.354776
C	-1.665	-0.07188	3.725594
C	-0.78407	0.877568	4.2045
C	-0.34513	1.286096	5.576398
C	0.457094	2.552652	5.260823
O	0.787578	2.497567	3.854924
C	-0.06137	1.613429	3.271458
C	-0.18675	1.447053	1.902199
C	-1.09178	0.485899	1.44839
O	0.085156	-3.19182	2.296817
H	3.578032	-0.57527	3.483463
H	2.985397	-1.70962	4.692791
H	2.062676	-0.24536	4.30982
H	2.603203	-2.53576	2.323457
H	1.668157	-1.10137	1.978873
H	0.771061	-1.81231	5.033347
H	-0.84293	-3.63009	5.678061
H	-1.4953	-3.4337	4.050379
H	-1.43775	-1.22118	6.169697
H	-2.78901	-2.3158	5.929792
H	-3.67457	-1.06984	1.556915
H	-2.35298	-2.25753	1.738157

H	0.281722	0.514399	6.036502
H	-1.1741	1.487975	6.257004
H	1.389604	2.631967	5.816906
H	-0.14237	3.451996	5.426614
H	0.395537	2.044782	1.212442
H	-1.21403	0.32456	0.383058

*XYZ Coordinates – Dihedral Scan Anti Conformer (**51**)*

C	2.47836	-0.00956	1.460158
C	1.834167	-1.14913	0.692258
C	0.332454	-1.12628	0.515914
N	-0.32944	-0.0835	1.035621
C	-1.7671	0.048984	0.978376
C	-2.44841	-0.53921	2.205279
C	-3.97546	-0.42346	2.19256
C	-4.65646	-1.10955	0.987262
C	-5.00537	0.001439	-0.01775
C	-5.031	1.24506	0.827123
C	-4.45123	0.999472	2.075473
C	-4.35514	2.025764	2.993599
C	-3.76109	2.11588	4.368414
C	-4.29831	3.472007	4.835932
O	-4.69052	4.19644	3.64808
C	-4.84654	3.281685	2.653952
C	-5.41974	3.552416	1.423726
C	-5.50423	2.507491	0.502185
O	-0.23509	-2.03472	-0.09232
H	2.110215	0.045667	2.48801
H	2.301445	0.955438	0.978317
H	3.55859	-0.15674	1.508924
H	2.062101	-2.10791	1.167667
H	2.250318	-1.21388	-0.31778
H	0.187081	0.604665	1.557393
H	-2.10606	-0.45625	0.073638
H	-2.0068	1.110128	0.880862
H	-2.05656	-0.04077	3.097918
H	-2.1768	-1.59647	2.285643
H	-4.32968	-0.86584	3.12901
H	-5.57765	-1.58818	1.327372

H	-4.02817	-1.88962	0.552069
H	-5.9556	-0.18356	-0.52461
H	-4.24411	0.088503	-0.80125
H	-2.66745	2.107002	4.321759
H	-4.0707	1.305589	5.030497
H	-3.56598	4.078208	5.366726
H	-5.18804	3.350353	5.460669
H	-5.79666	4.54134	1.190938
H	-5.9527	2.69106	-0.46885

*XYZ Coordinates – Double Dihedral Anti Conformer (**52**)*

C	2.468361	-1.01929	0.616578
C	2.020753	-0.12689	1.75935
C	0.55361	-0.12459	2.124842
N	-0.25836	-0.9161	1.410269
C	-1.68526	-0.97554	1.623672
C	-2.44939	0.064524	0.81621
C	-3.97128	-0.01959	1.061636
C	-4.81248	-0.06807	-0.22808
C	-5.15901	1.392715	-0.54965
C	-5.16621	2.038355	0.809359
C	-4.50054	1.220967	1.730242
C	-4.37066	1.636707	3.038922
C	-3.68532	1.047912	4.236488
C	-4.21704	1.947094	5.356733
O	-4.71631	3.148519	4.726606
C	-4.91557	2.861366	3.412486
C	-5.57528	3.689738	2.522124
C	-5.69182	3.260327	1.198202
O	0.145469	0.57736	3.050851
H	1.98418	-0.74715	-0.32503
H	2.261005	-2.07311	0.819343
H	3.545045	-0.92166	0.467763
H	2.27587	0.916797	1.55213
H	2.554805	-0.38498	2.678889
H	0.126895	-1.43239	0.63719
H	-1.87279	-0.83617	2.68964
H	-2.02642	-1.97804	1.354937
H	-2.23548	-0.09226	-0.24526
H	-2.07052	1.05735	1.072175

H	-4.16673	-0.90644	1.673771
H	-5.73805	-0.61496	-0.02792
H	-4.29379	-0.57774	-1.042
H	-6.10896	1.493002	-1.07942
H	-4.38972	1.850035	-1.18347
H	-2.59773	1.121312	4.136845
H	-3.93164	-0.00165	4.405849
H	-3.45996	2.243591	6.081074
H	-5.05268	1.474515	5.881174
H	-5.99277	4.635516	2.846808
H	-6.20756	3.888906	0.47943

^{1}H NMR (500 MHz, CDCl3) spectrum of *tert*-butyl ((2-bromobenzyl)oxy)carbamate (**17**):

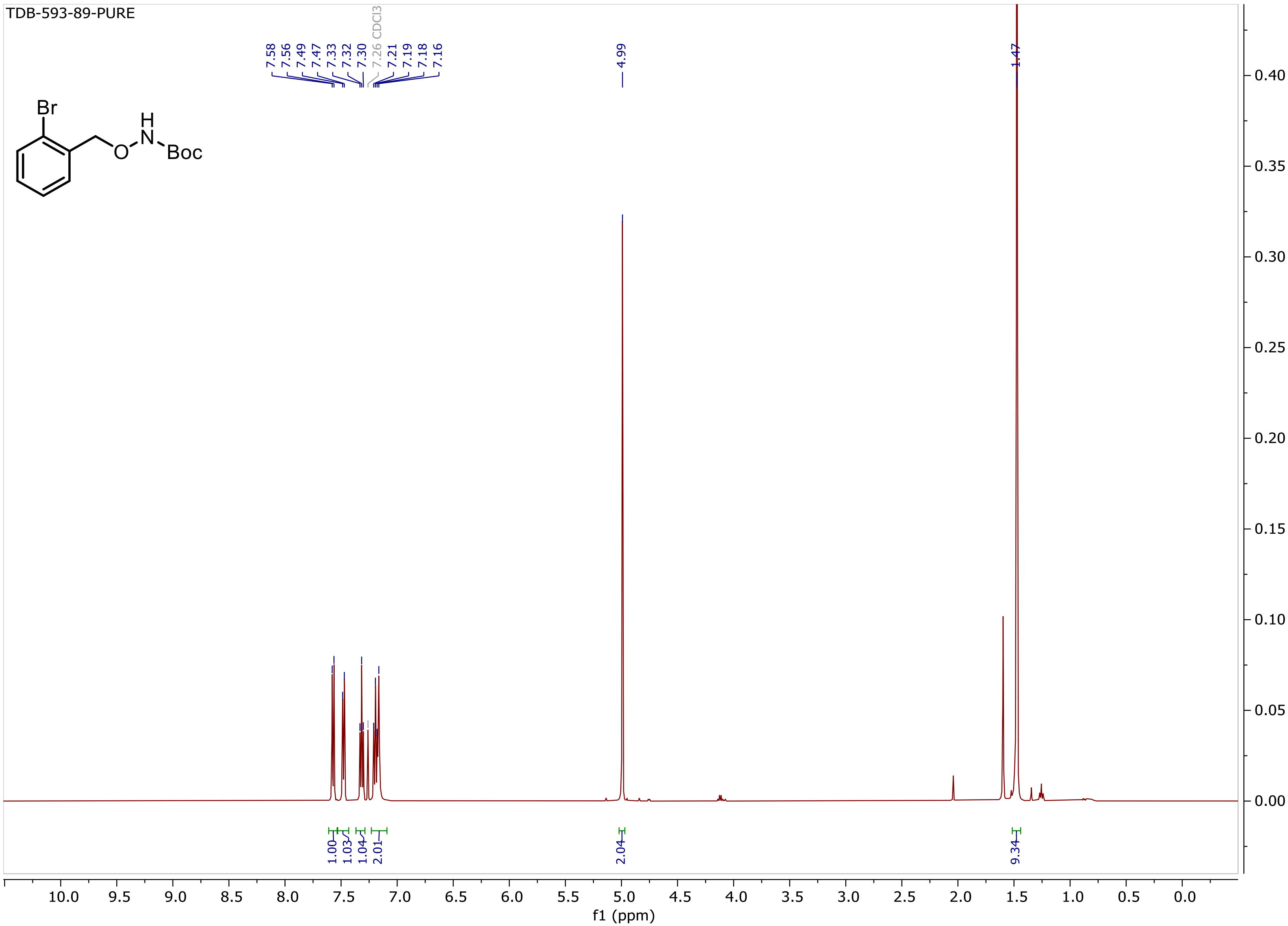

^{13}C NMR (126 MHz, $CDCl_3$) spectrum of *tert*-butyl ((2-bromobenzyl)oxy)carbamate (**17**):

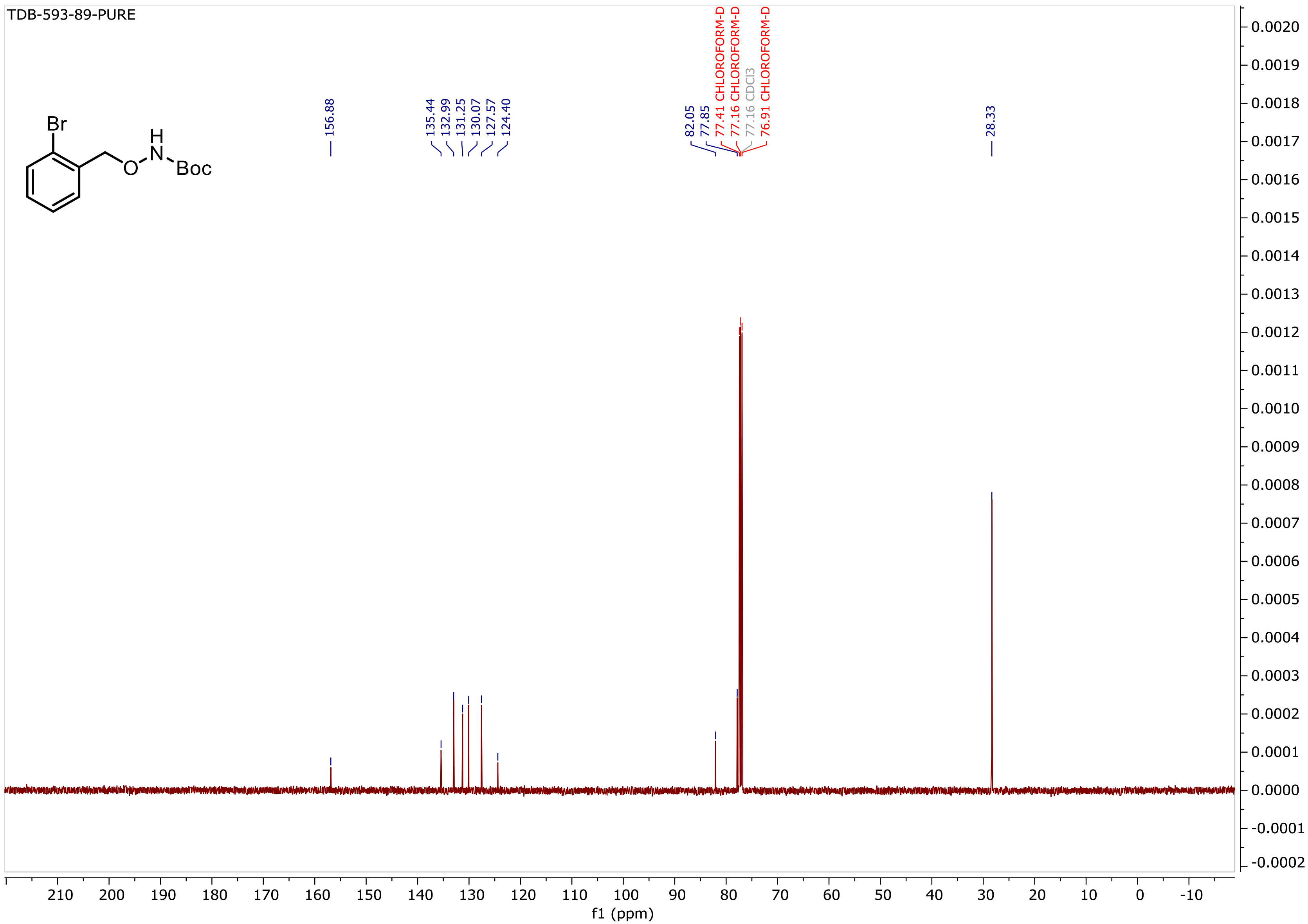

^{1}H NMR (500 MHz, CDCl₃) spectrum of *tert*-butyl benzo[*c*]isoxazole-1(3*H*)-carboxylate (**18**):

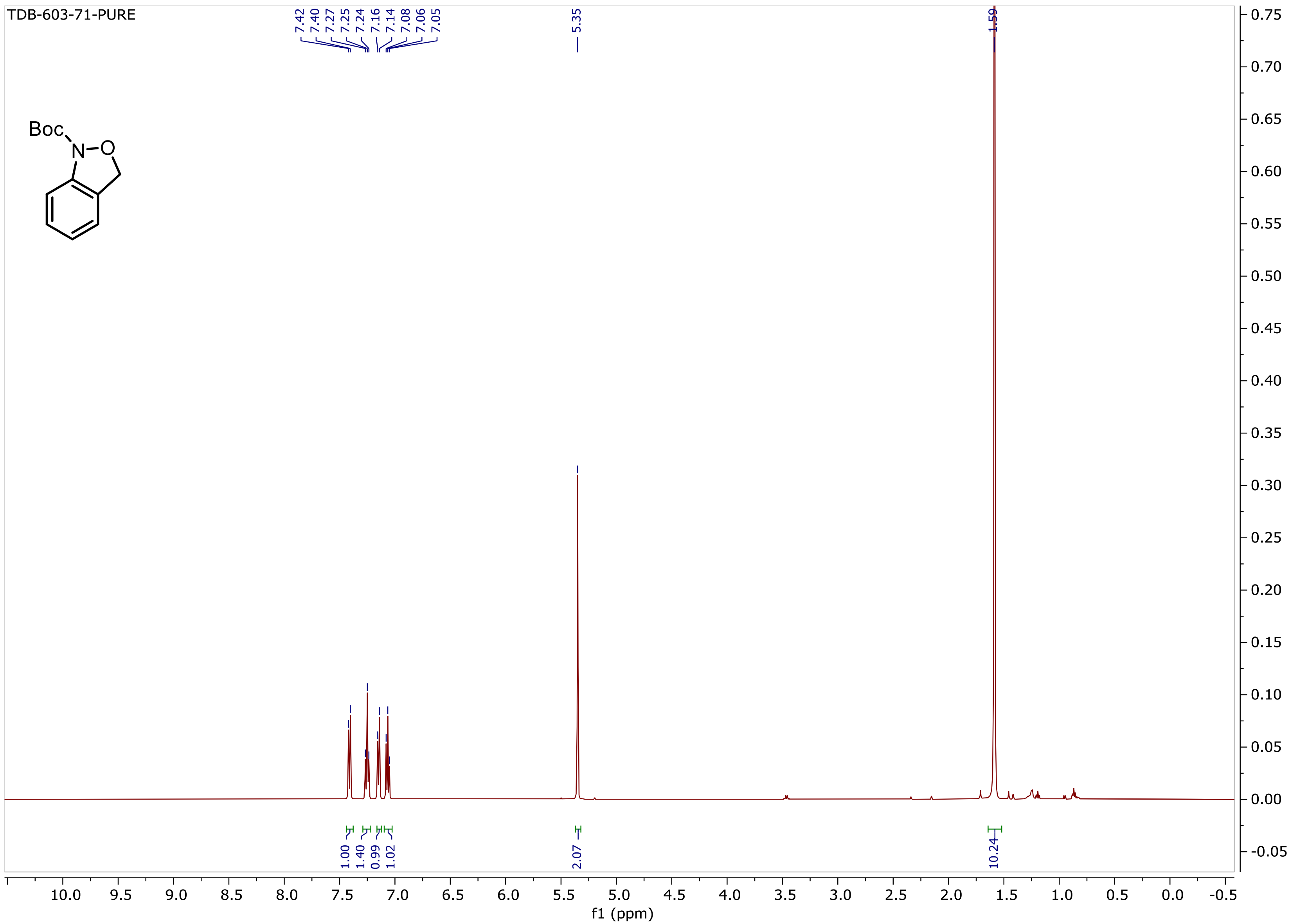

^{13}C NMR (126 MHz, CDCl$_3$) spectrum of *tert*-butyl benzo[*c*]isoxazole-1(3*H*)-carboxylate (**18**):

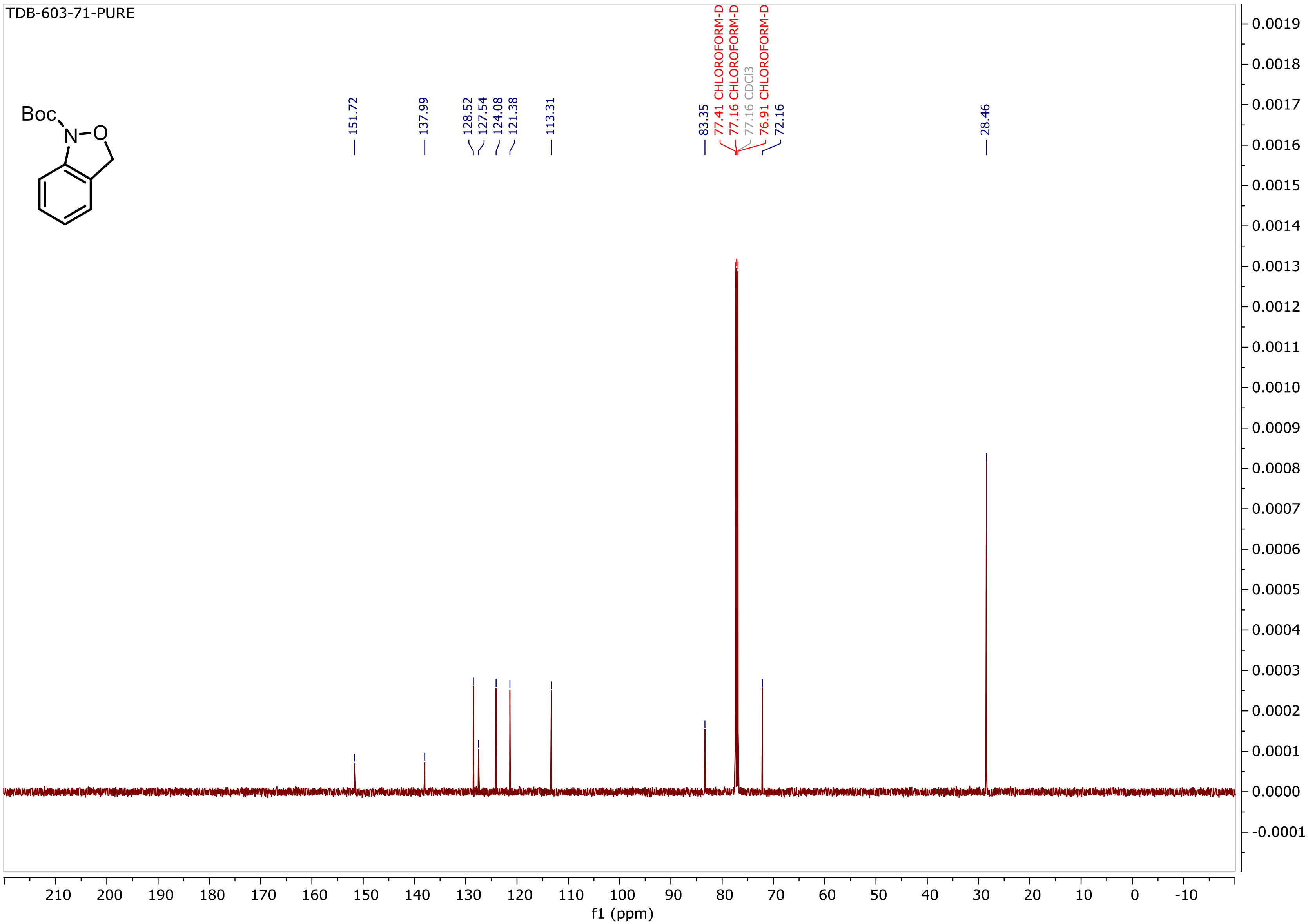

^{1}H NMR (500 MHz, CDCl₃) spectrum of 2-bromo-1-(bromomethyl)-3-methylbenzene:

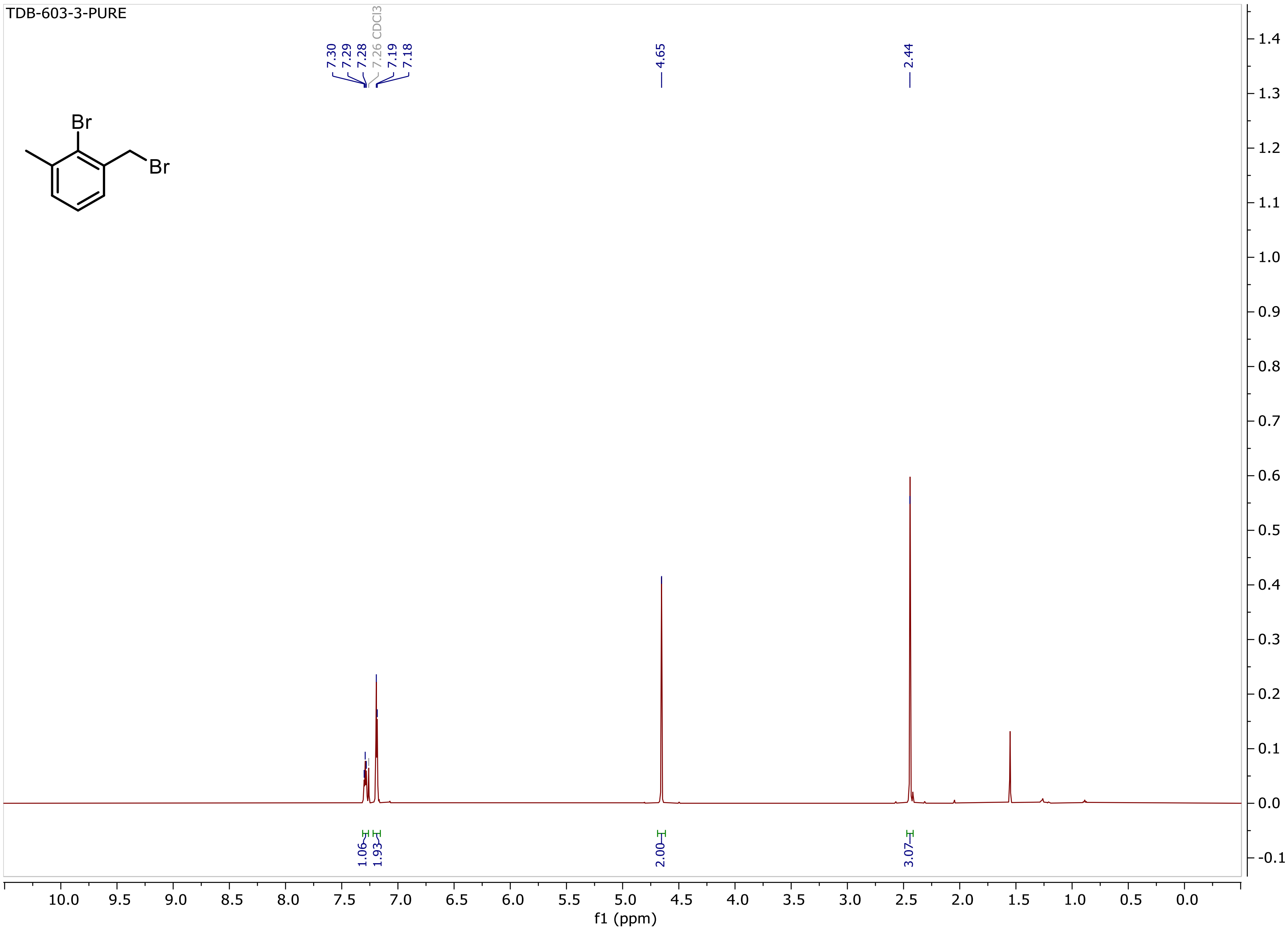

^{13}C NMR (126 MHz, $CDCl_3$) spectrum of 2-bromo-1-(bromomethyl)-3-methylbenzene:

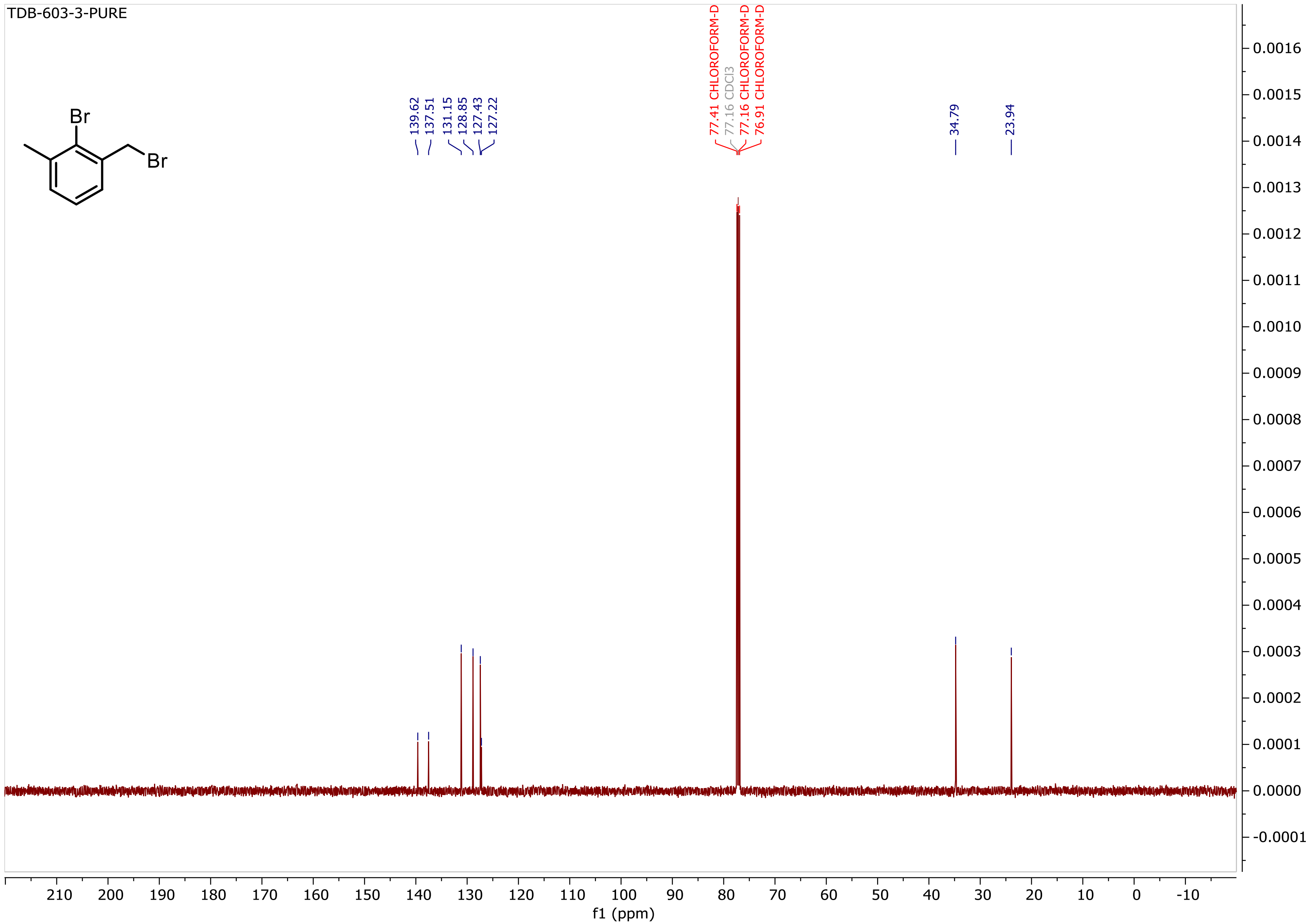

^{1}H NMR (500 MHz, $CDCl_3$) spectrum of *tert*-butyl ((2-bromo-3-methylbenzyl)oxy)carbamate (**19**):

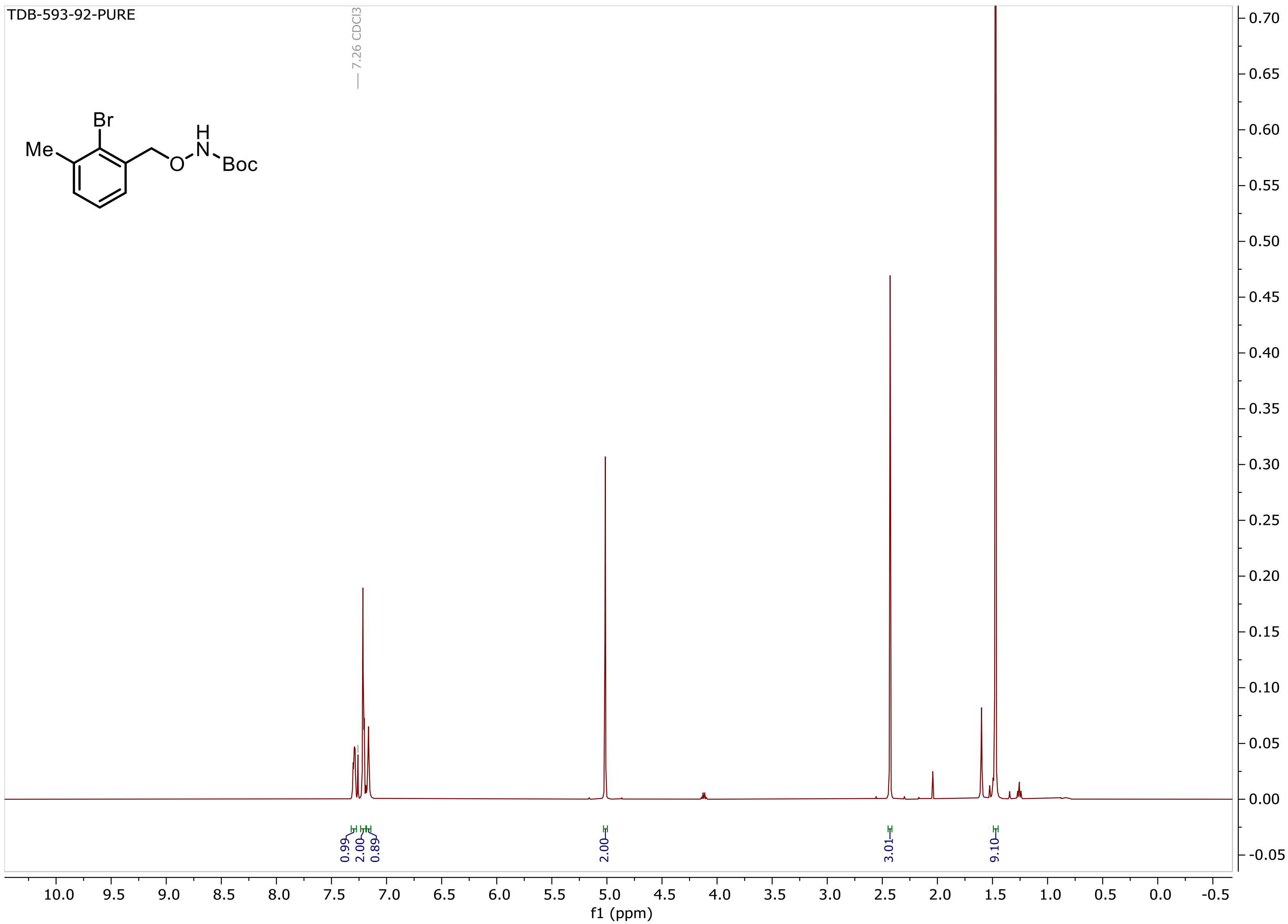

^{13}C NMR (126 MHz, $CDCl_3$) spectrum of *tert*-butyl ((2-bromo-3-methylbenzyl)oxy)carbamate (**19**):

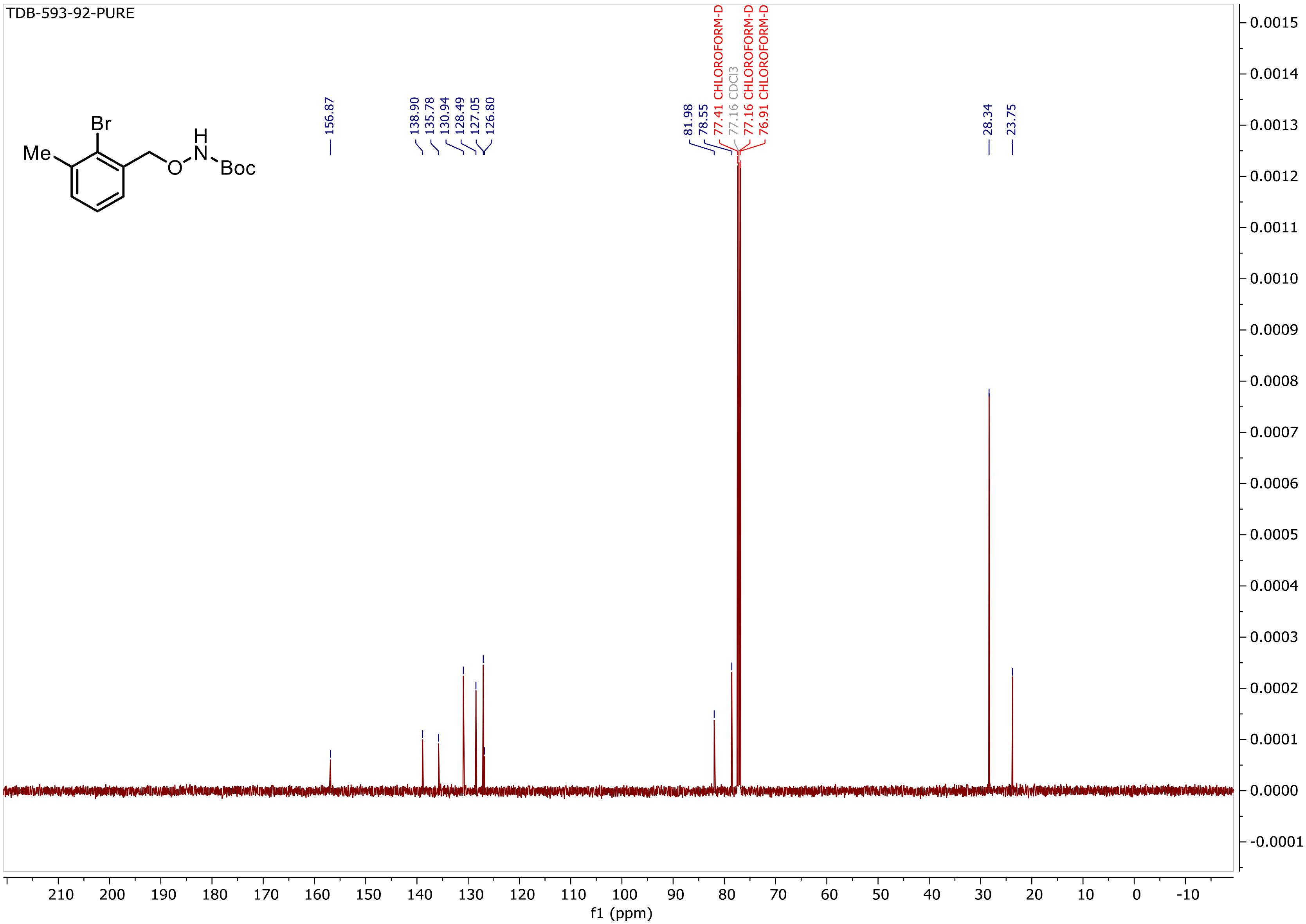

^{1}H NMR (500 MHz, CDCl$_3$) spectrum of *tert*-butyl 7-methylbenzo[*c*]isoxazole-1(3*H*)-carboxylate (**20**):

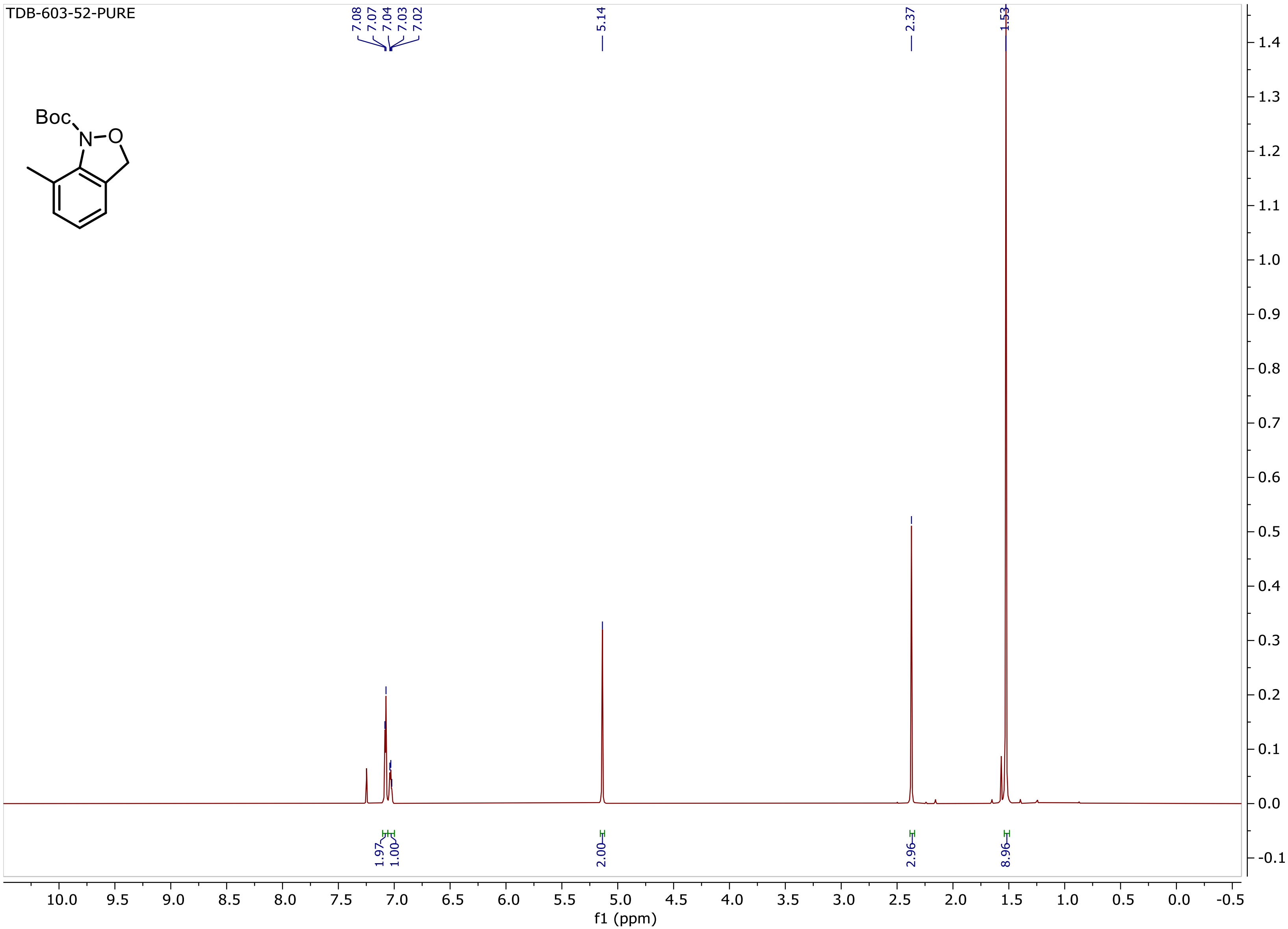

^{13}C NMR (126 MHz, CDCl$_3$) spectrum of *tert*-butyl 7-methylbenzo[*c*]isoxazole-1(3*H*)-carboxylate (**20**):

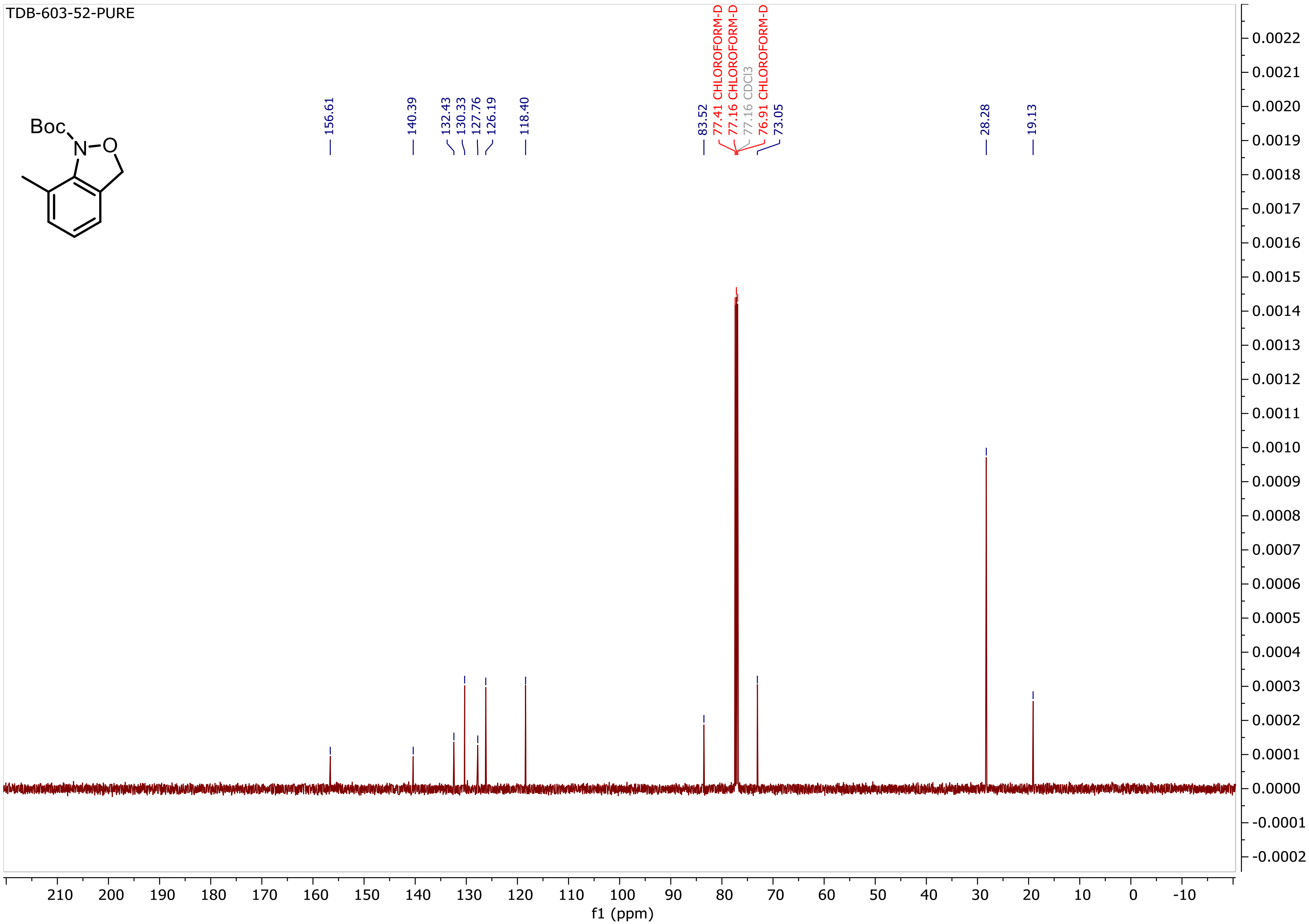

^{1}H NMR (500 MHz, $CDCl_3$) spectrum of *tert*-butyl ((4-bromo-2,3-dihydrobenzofuran-5-yl)methoxy)carbamate (**21**):

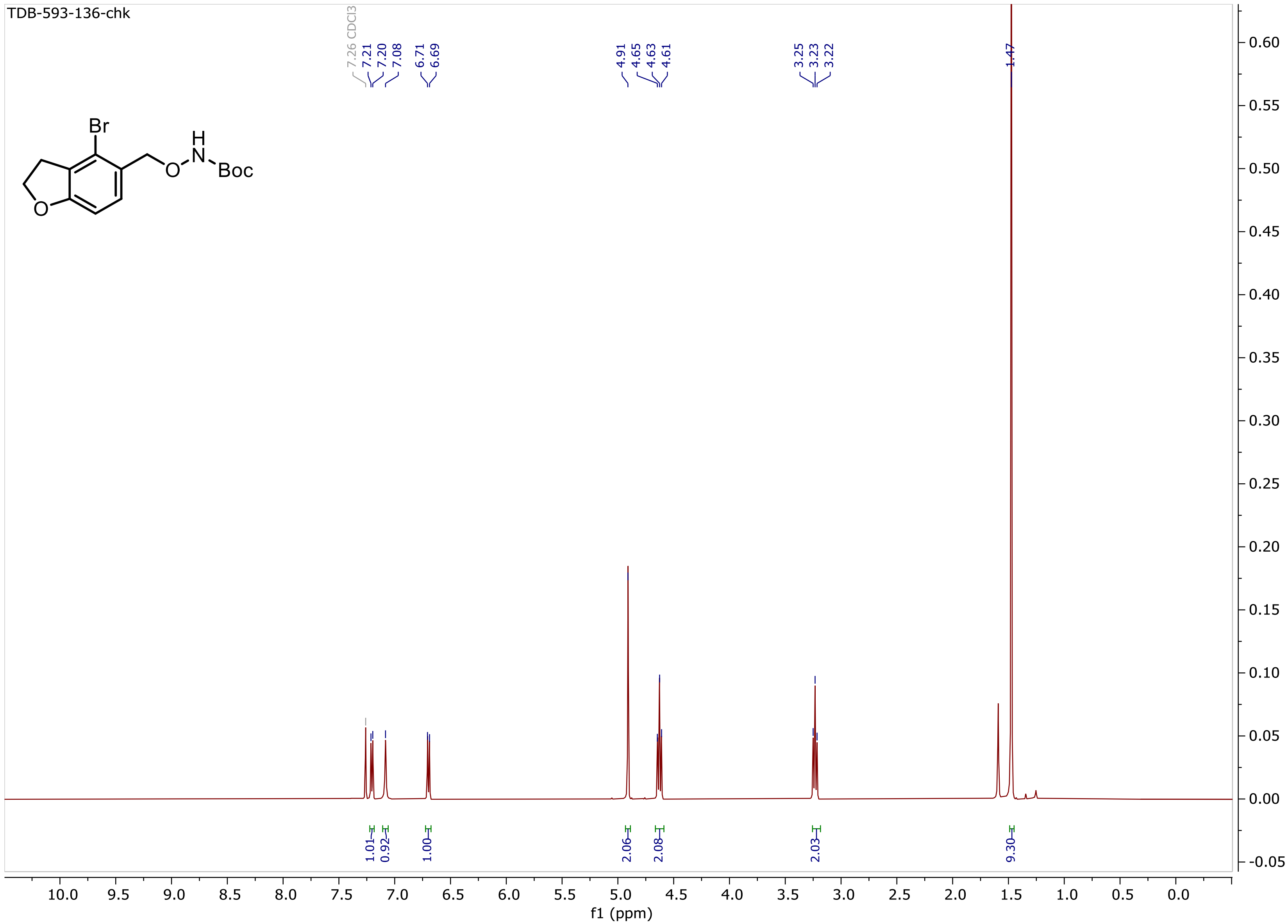

^{13}C NMR (126 MHz, CDCl$_3$) spectrum of *tert*-butyl ((4-bromo-2,3-dihydrobenzofuran-5-yl)methoxy)carbamate (**21**):

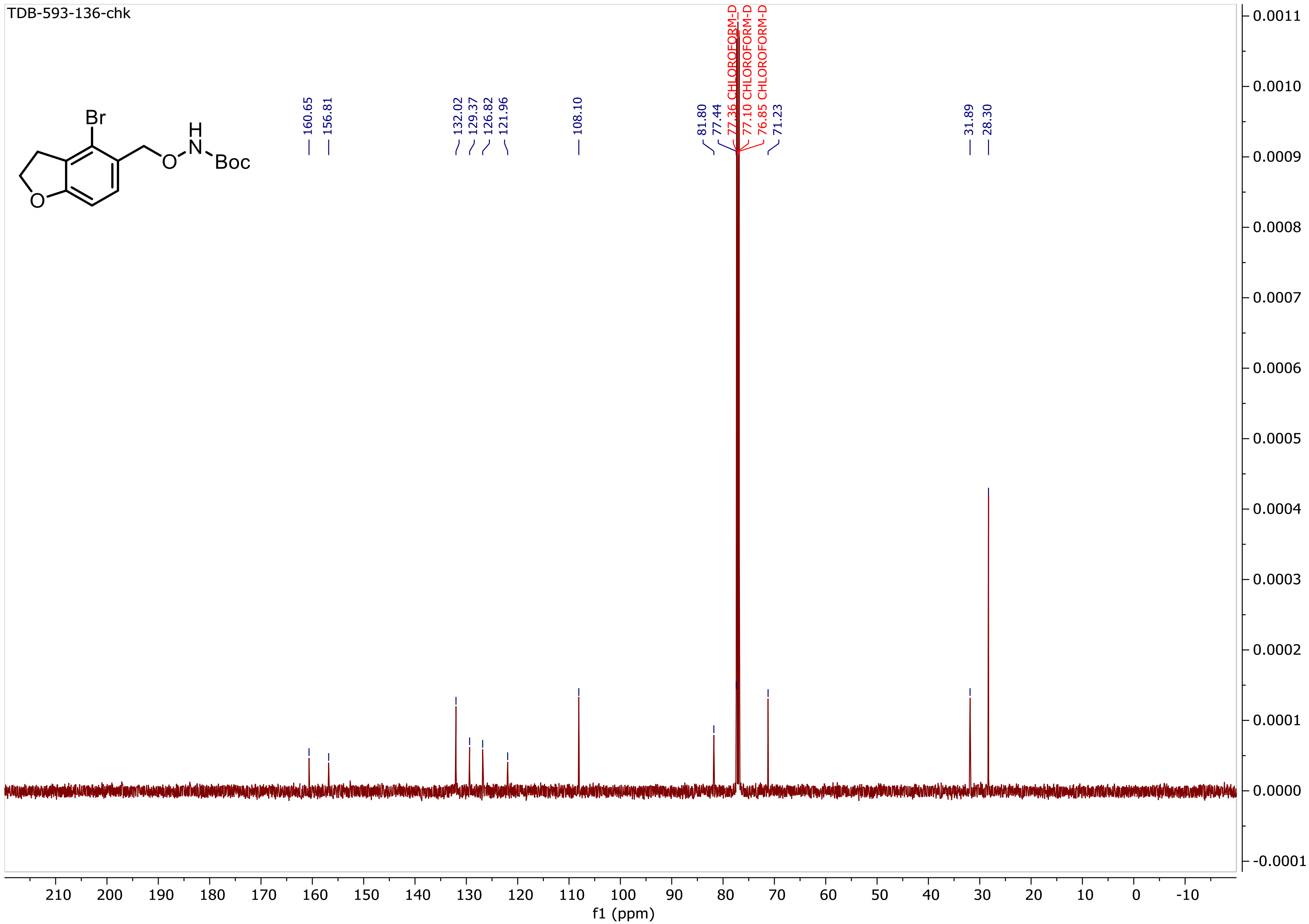

^{1}H NMR (500 MHz, $CDCl_3$) spectrum *tert*-butyl 7,8-dihydrobenzofuro[4,5-*c*]isoxazole-1(3*H*)-carboxylate (**22**):

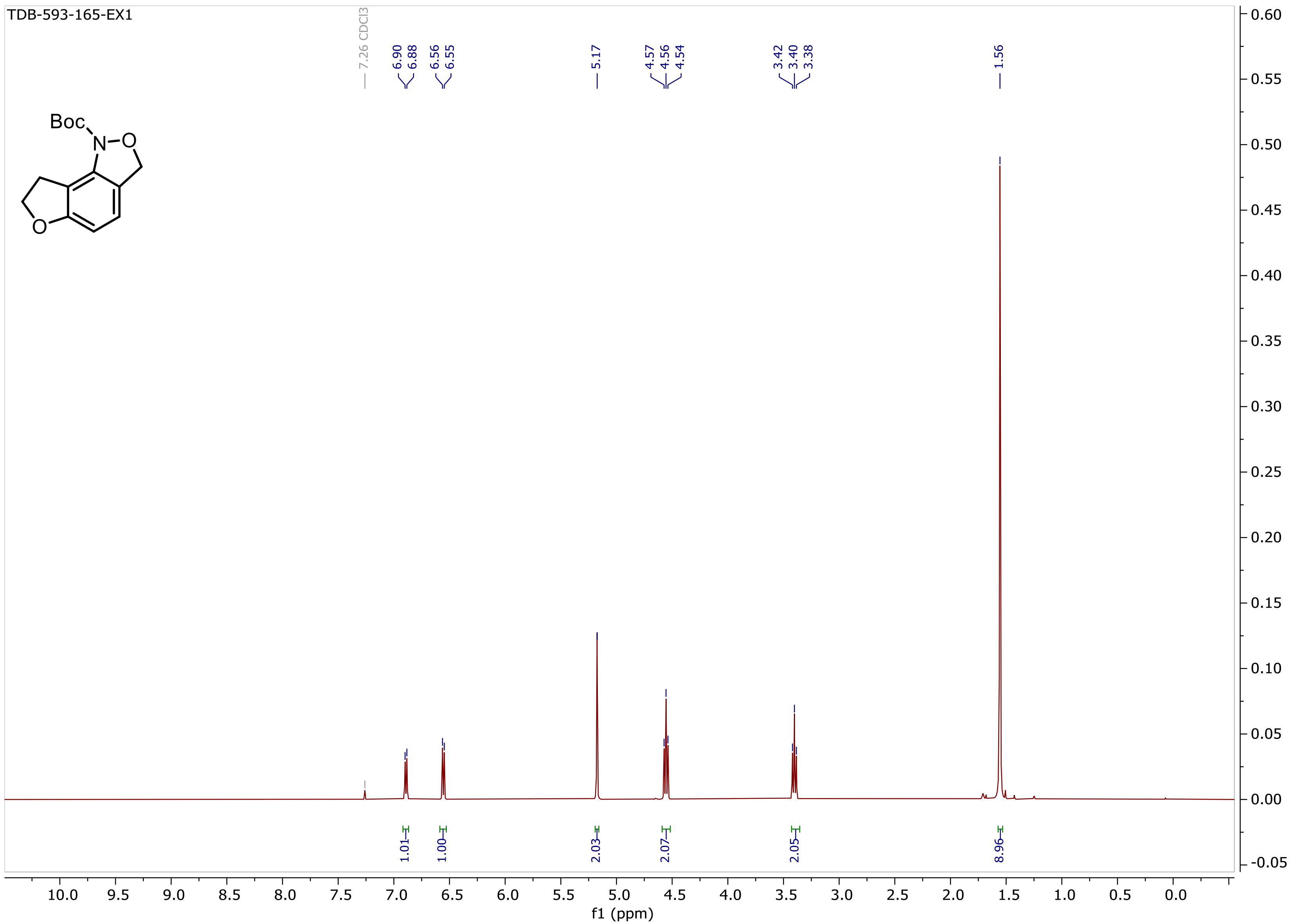

^{13}C NMR (126 MHz, CDCl$_3$) spectrum *tert*-butyl 7,8-dihydrobenzofuro[4,5-*c*]isoxazole-1(*3H*)-carboxylate (**22**):

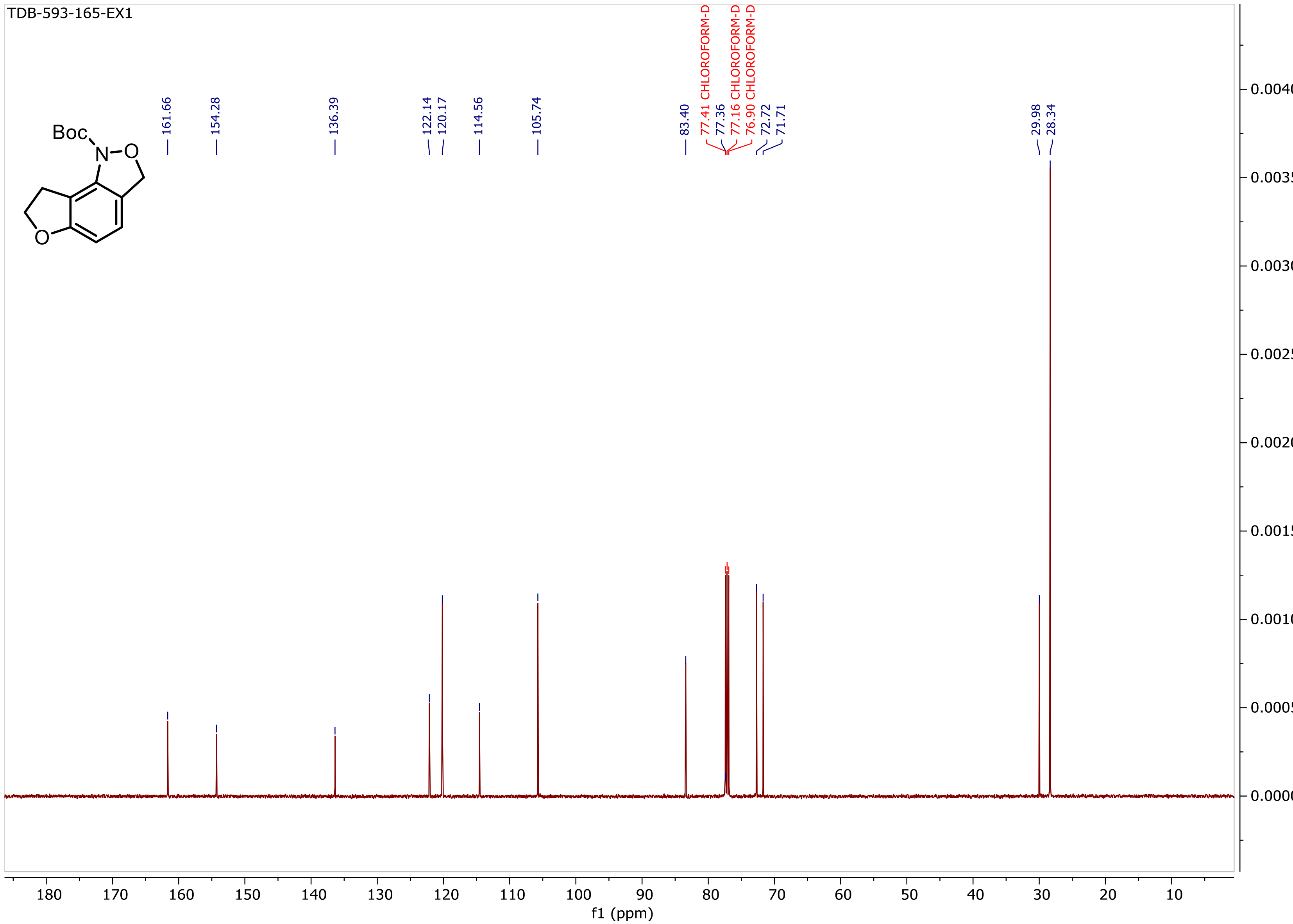

^{1}H NMR (500 MHz, CDCl3) spectrum of *tert*-butyl ((2-bromo-3-nitrobenzyl)oxy)carbamate (**S1**):

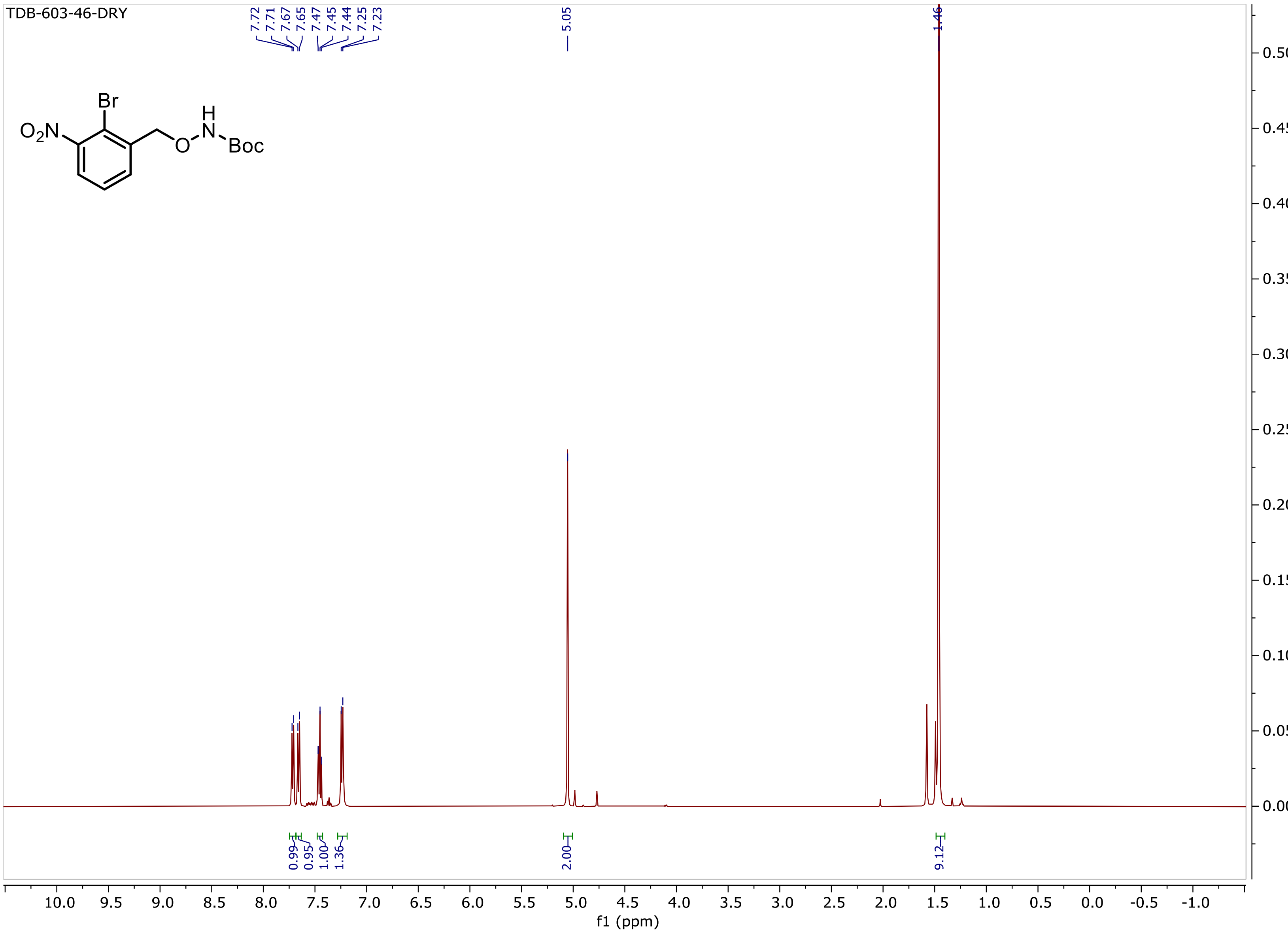

^{13}C NMR (126 MHz, $CDCl_3$) spectrum of *tert*-butyl ((2-bromo-3-nitrobenzyl)oxy)carbamate (**S1**):

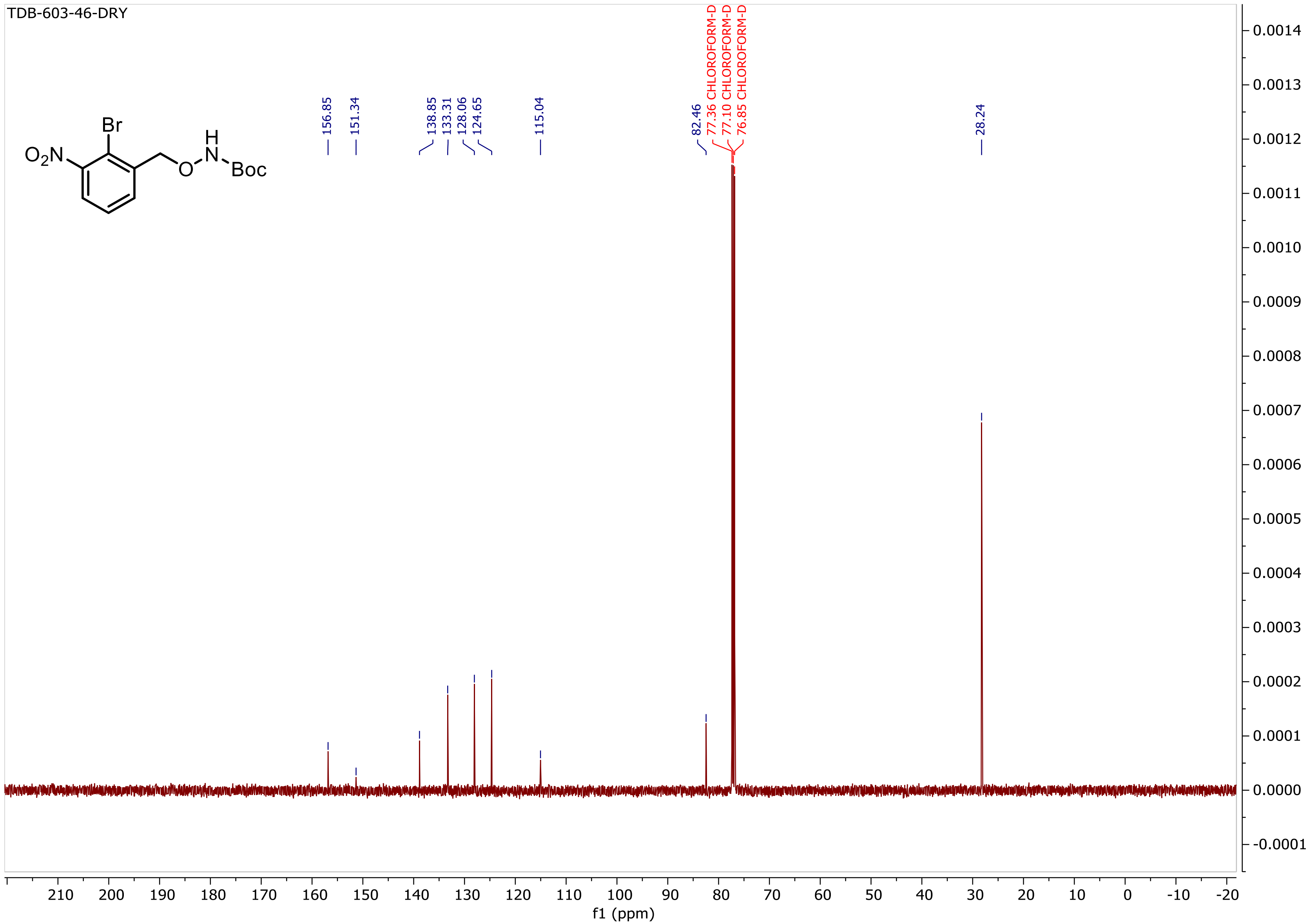

^{1}H NMR (500 MHz, CDCl$_3$) spectrum of *tert*-butyl 7-nitrobenzo[*c*]isoxazole-1(3*H*)-carboxylate (**23**):

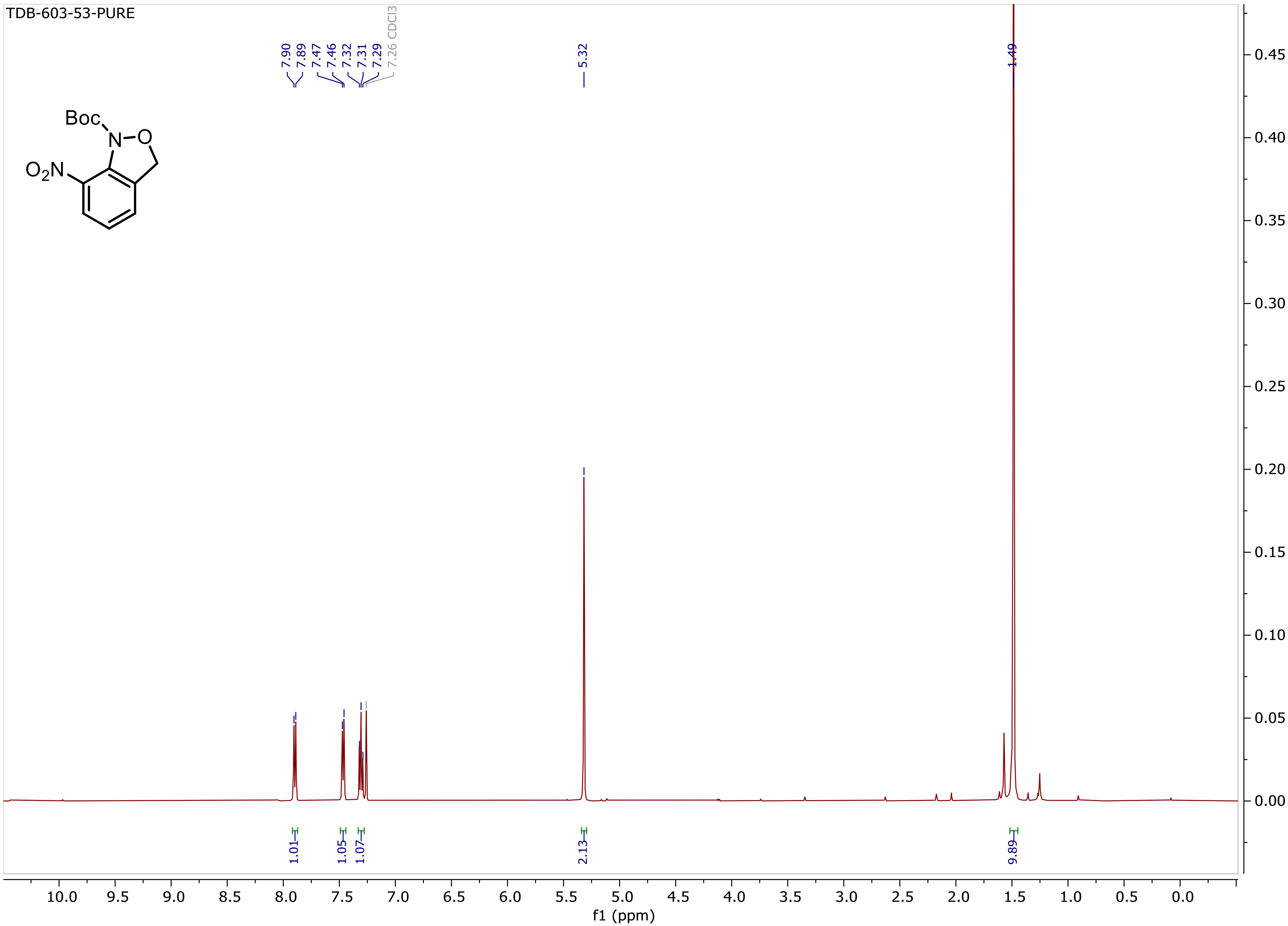

^{13}C NMR (126 MHz, CDCl$_3$) spectrum of *tert*-butyl 7-nitrobenzo[*c*]isoxazole-1(3*H*)-carboxylate (**23**):

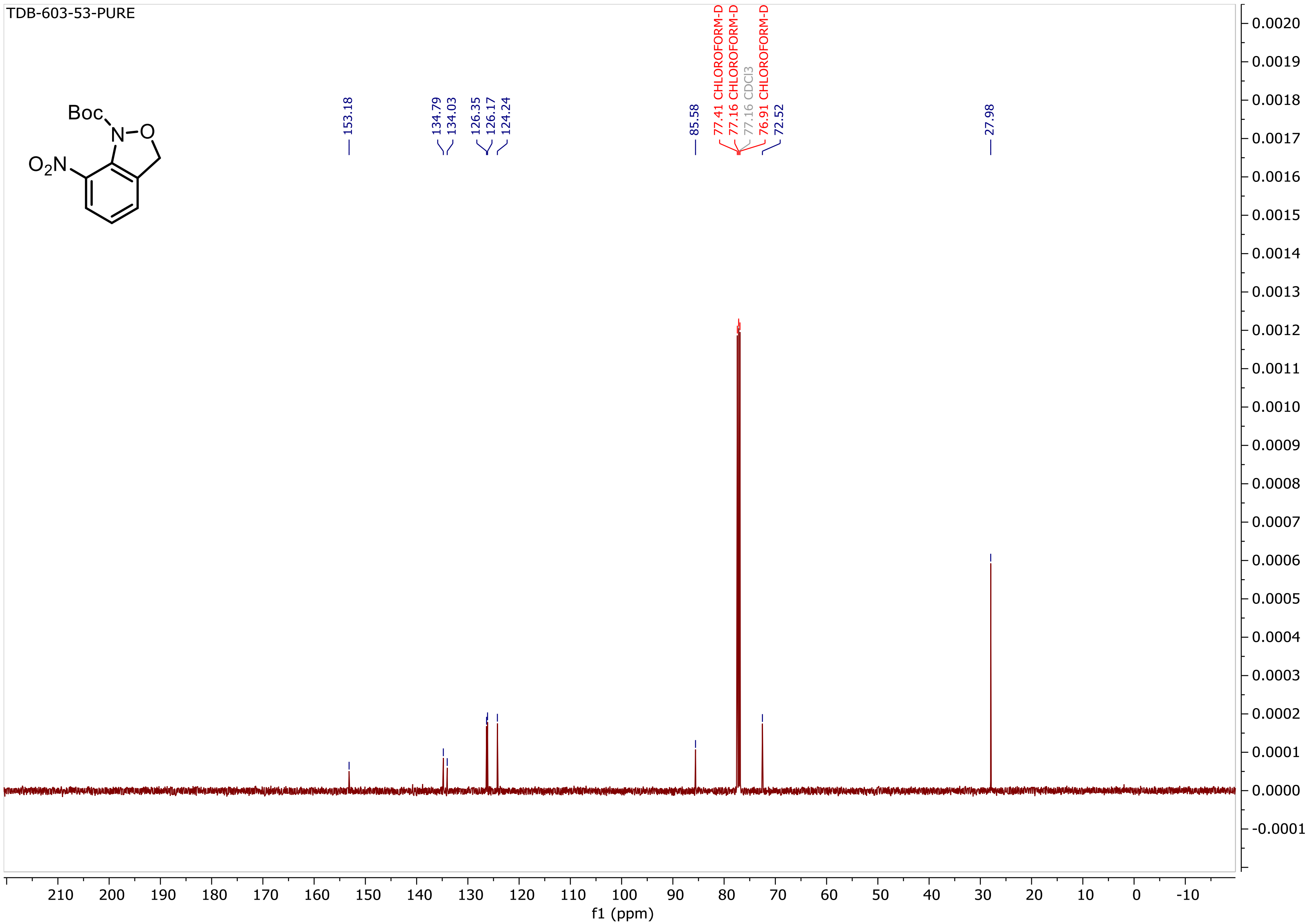

^{1}H NMR (500 MHz, $CDCl_3$) spectrum of *tert*-butyl ((3-bromopyridin-2-yl)methoxy)carbamate (**S2**):

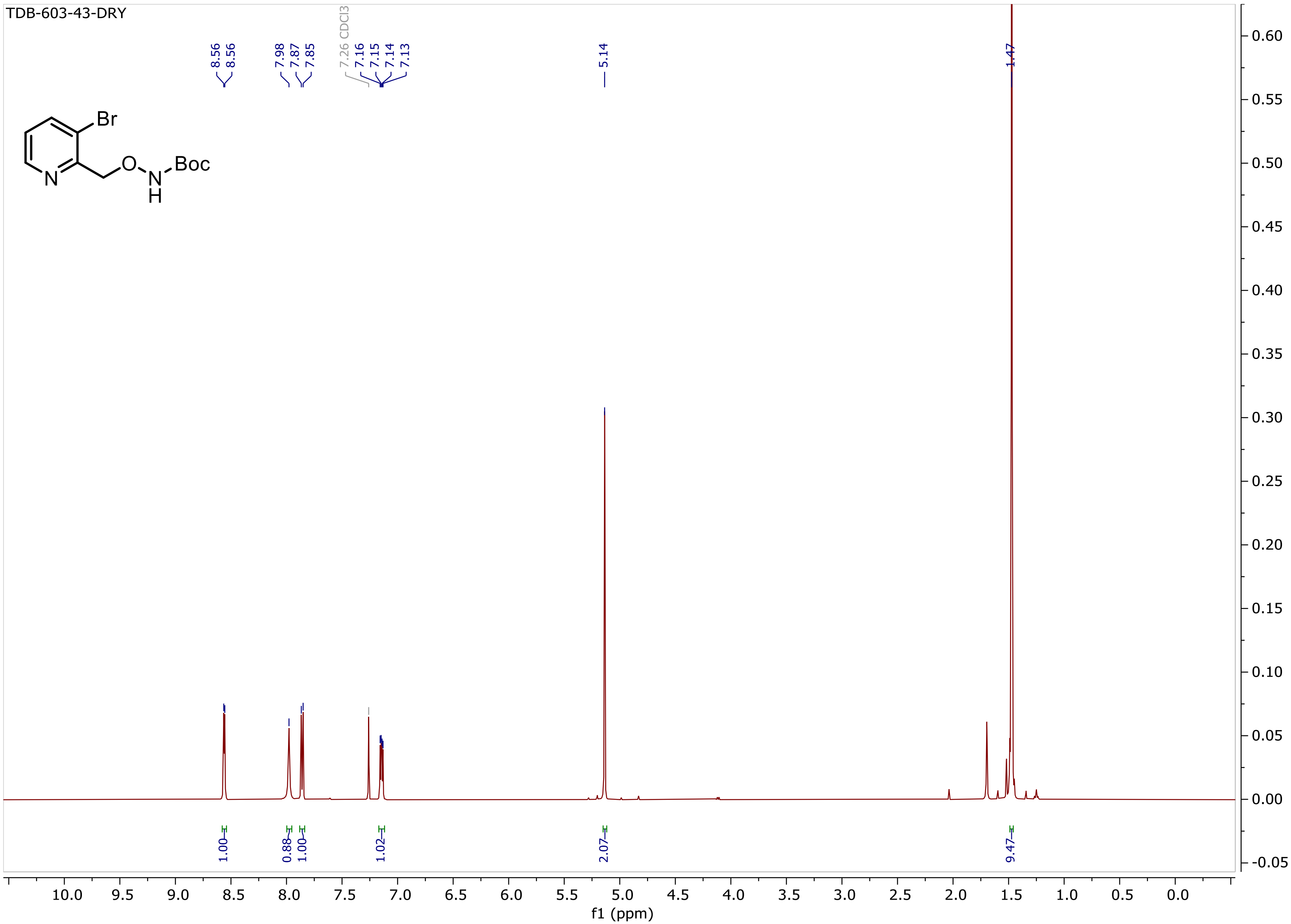

^{13}C NMR (126 MHz, $CDCl_3$) spectrum of *tert*-butyl ((3-bromopyridin-2-yl)methoxy)carbamate (**S2**):

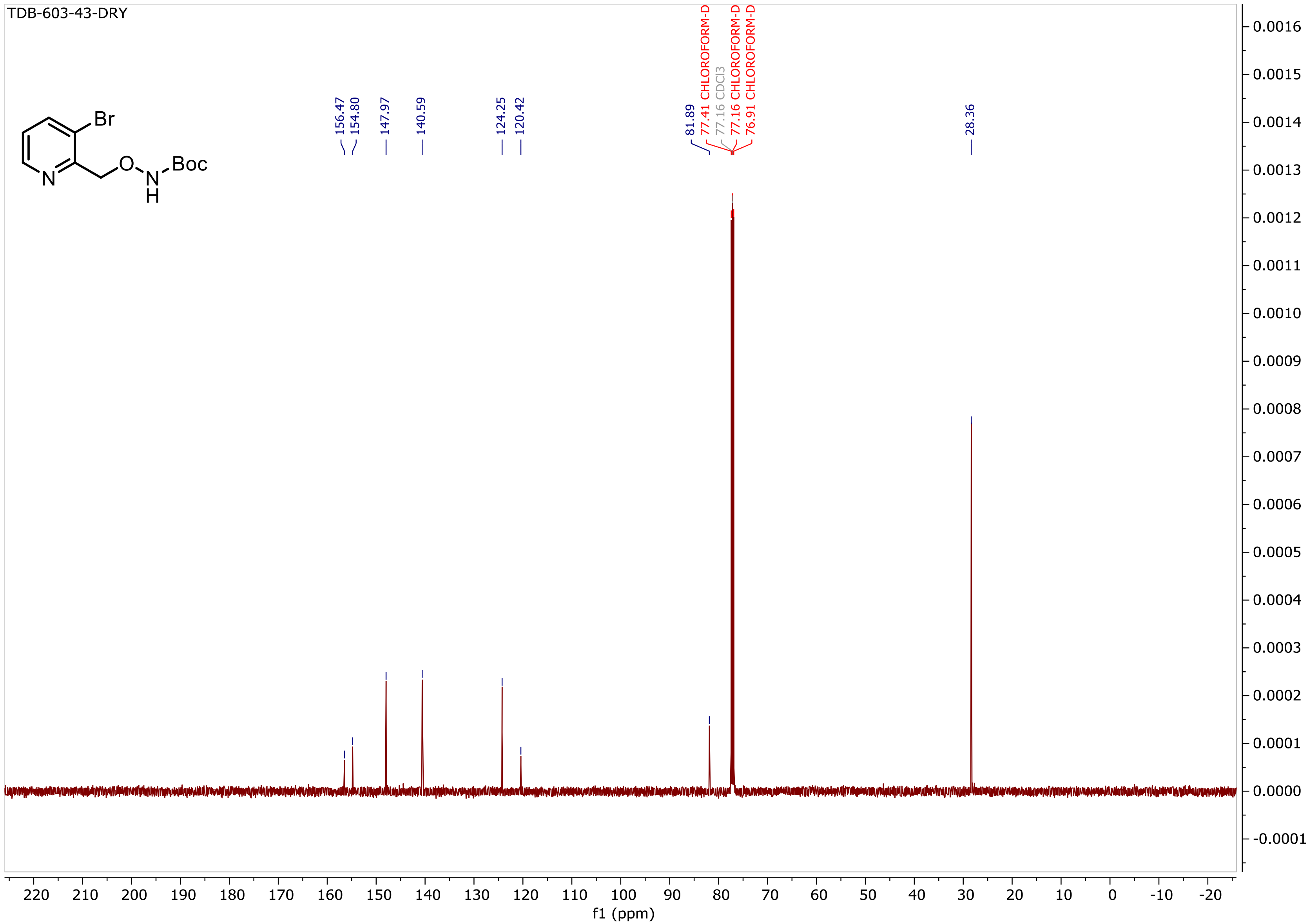

^{1}H NMR (500 MHz, CDCl$_3$) spectrum of *tert*-butyl isoxazolo[4,3-*b*]pyridine-1(3*H*)-carboxylate (**24**):

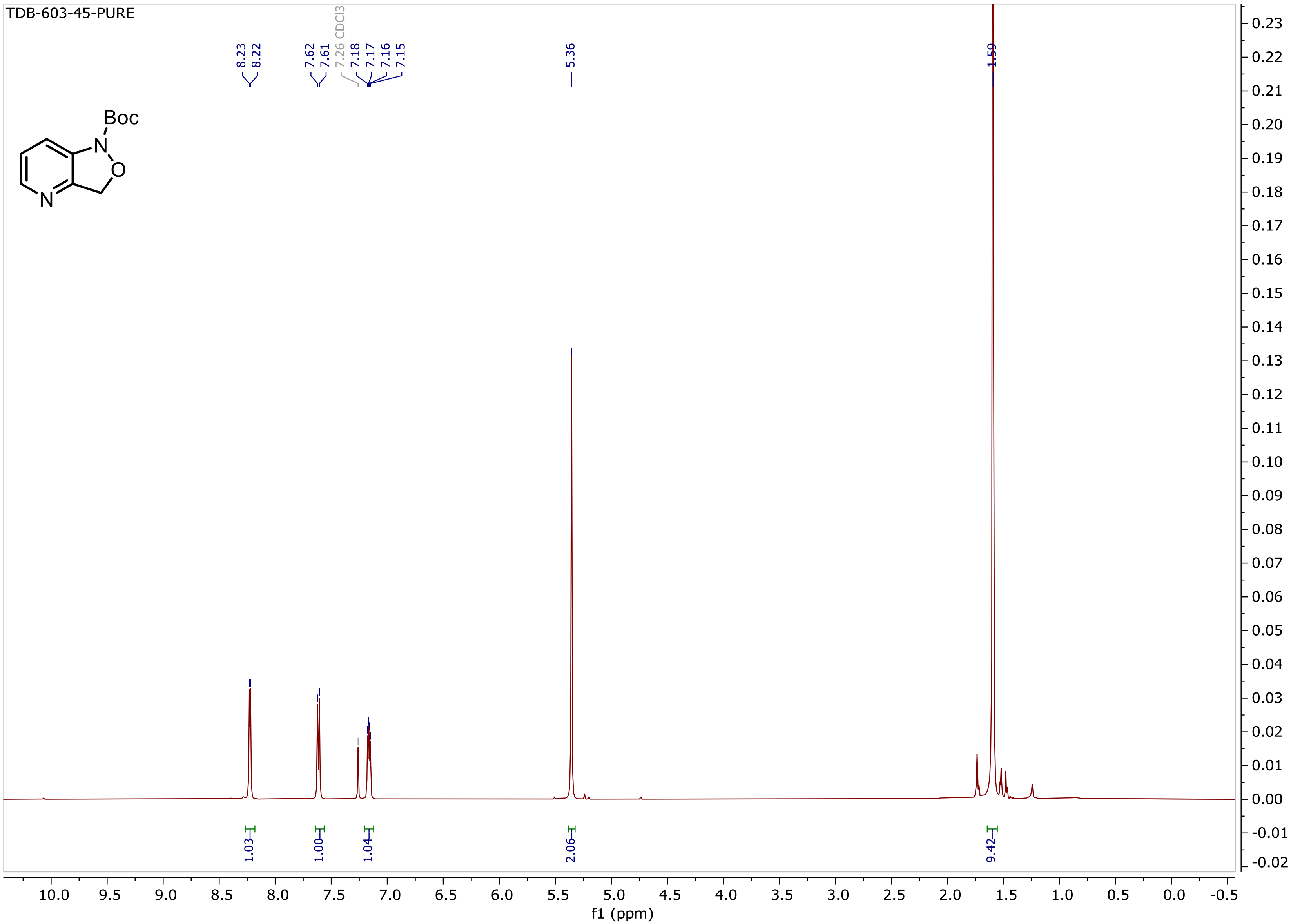

^{13}C NMR (126 MHz, CDCl$_3$) spectrum of *tert*-butyl isoxazolo[4,3-*b*]pyridine-1(3*H*)-carboxylate (**24**):

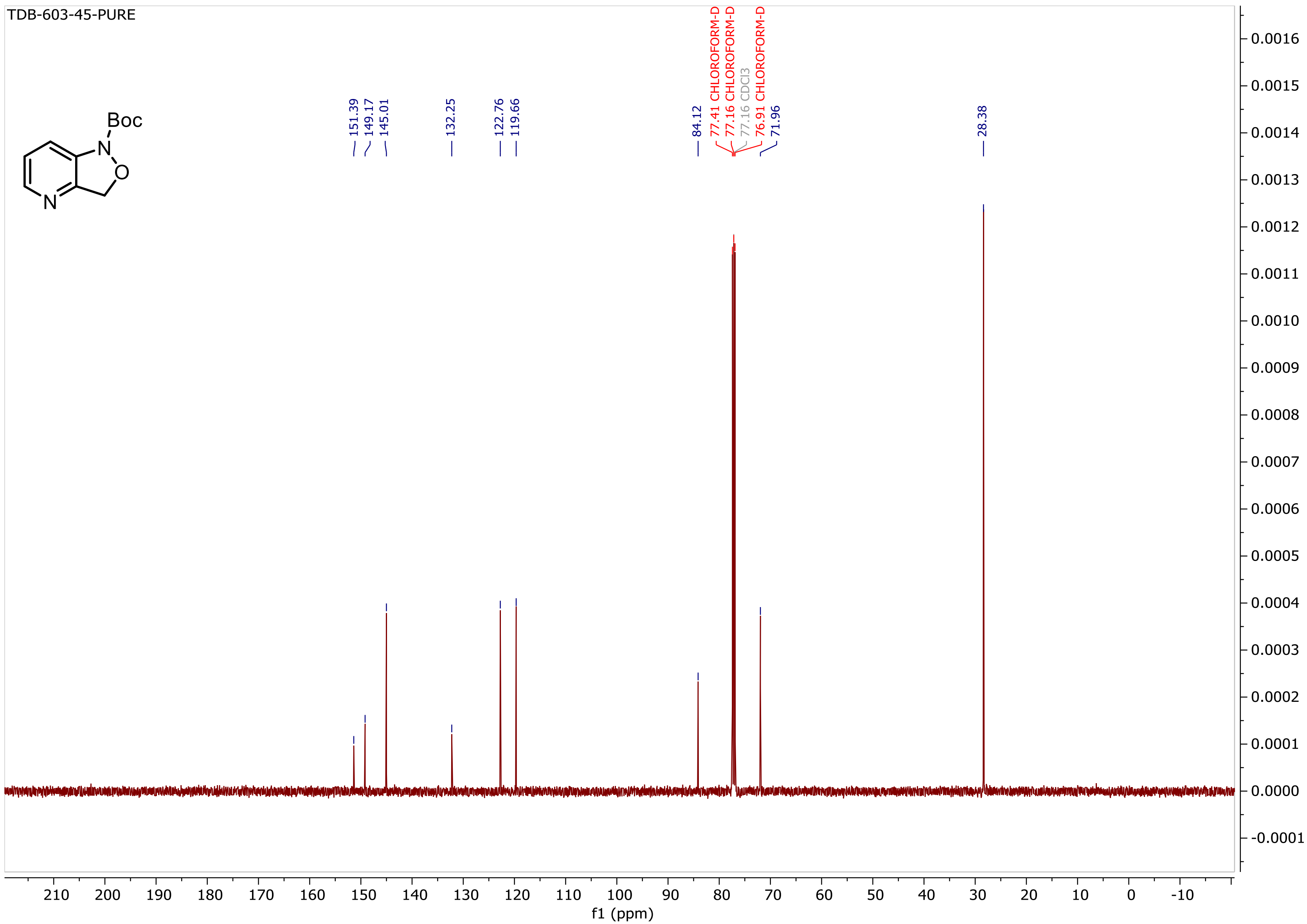

^{1}H NMR (500 MHz, $CDCl_3$) spectrum of 2-bromo-3-(bromomethyl)pyrazine*:*

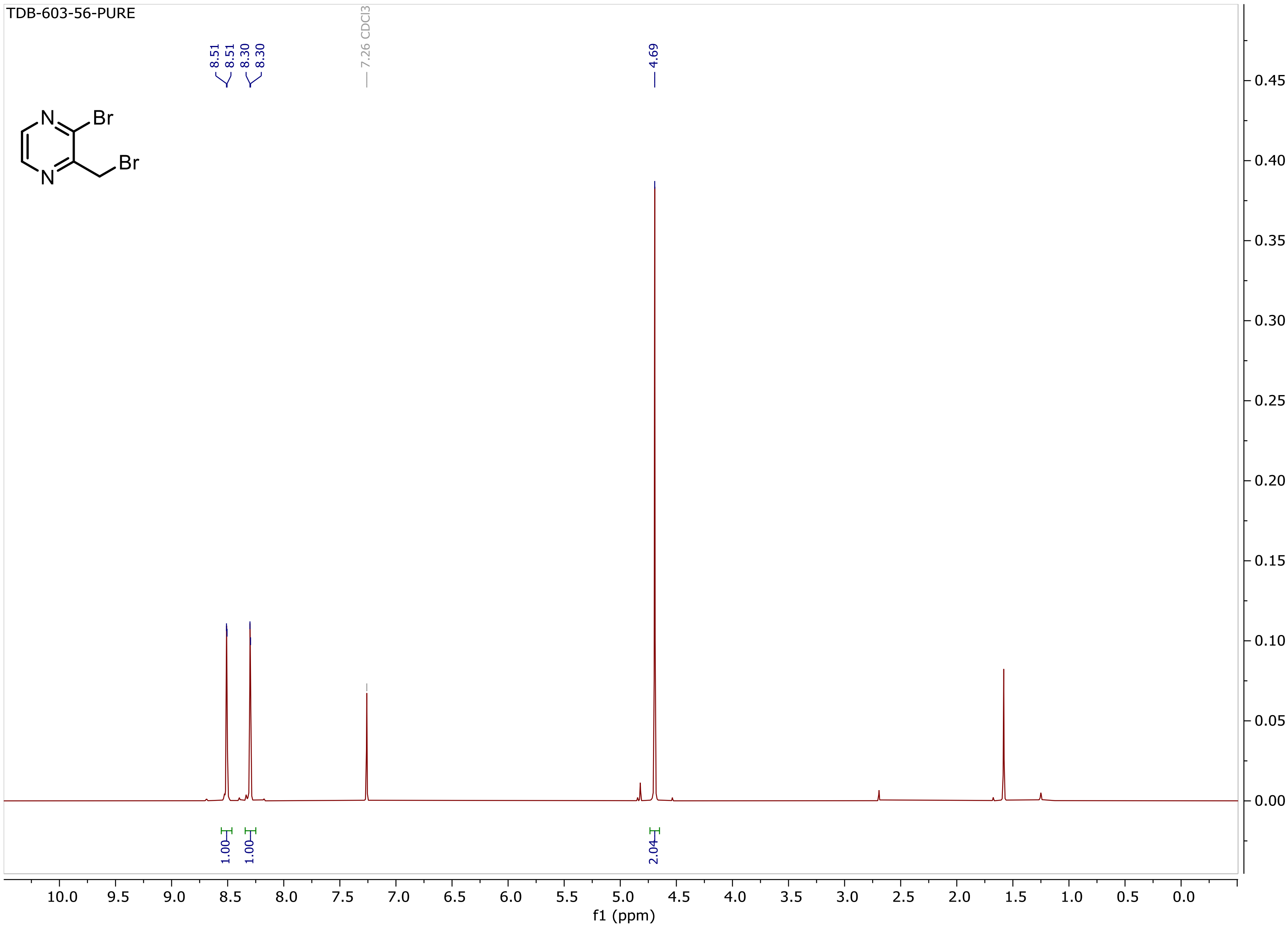

^{13}C NMR (126 MHz, $CDCl_3$) spectrum of 2-bromo-3-(bromomethyl)pyrazine:

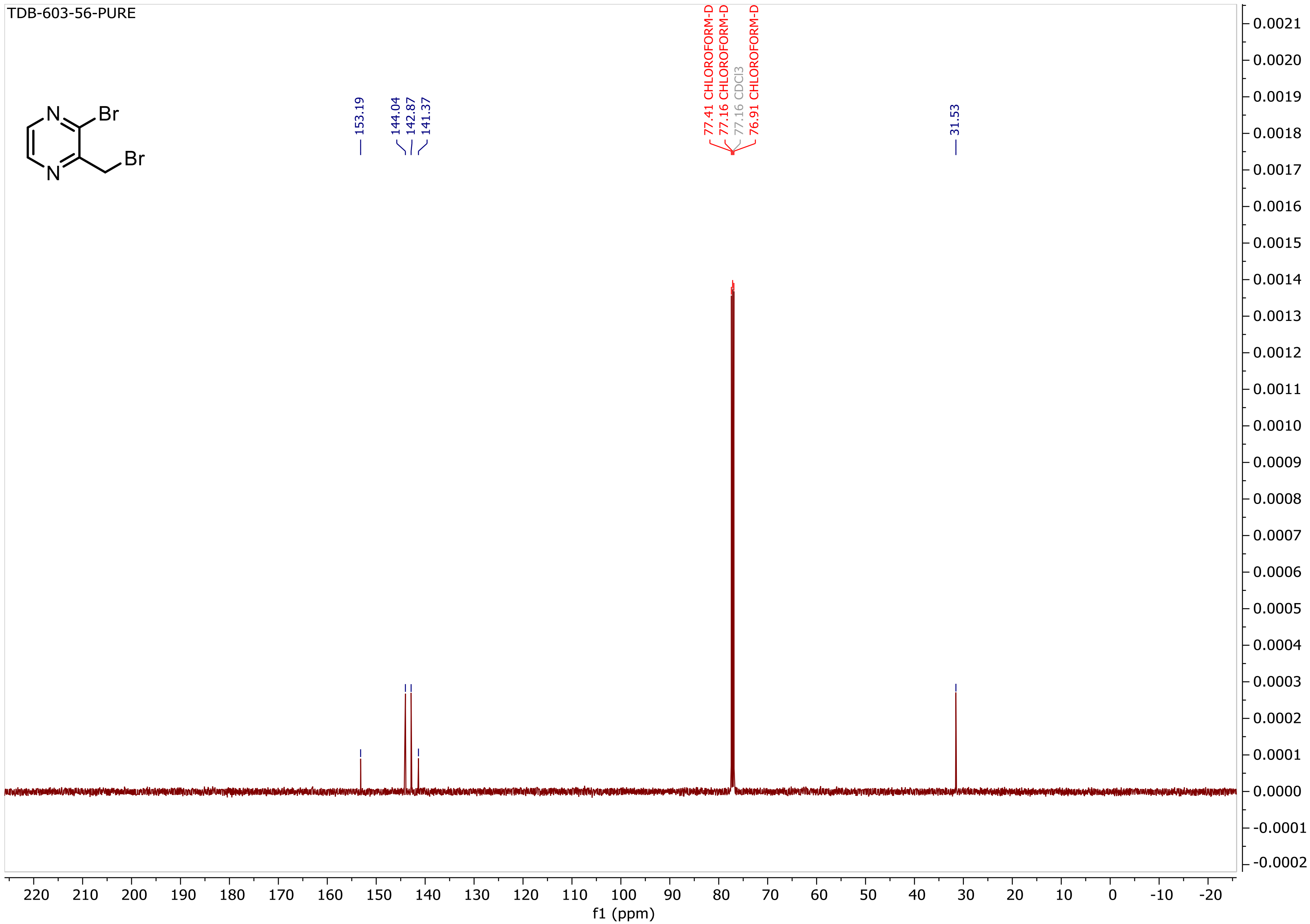

^{1}H NMR (500 MHz, CDCl₃) spectrum of *tert*-butyl ((3-bromopyrazin-2-yl)methoxy)carbamate (**S3**):

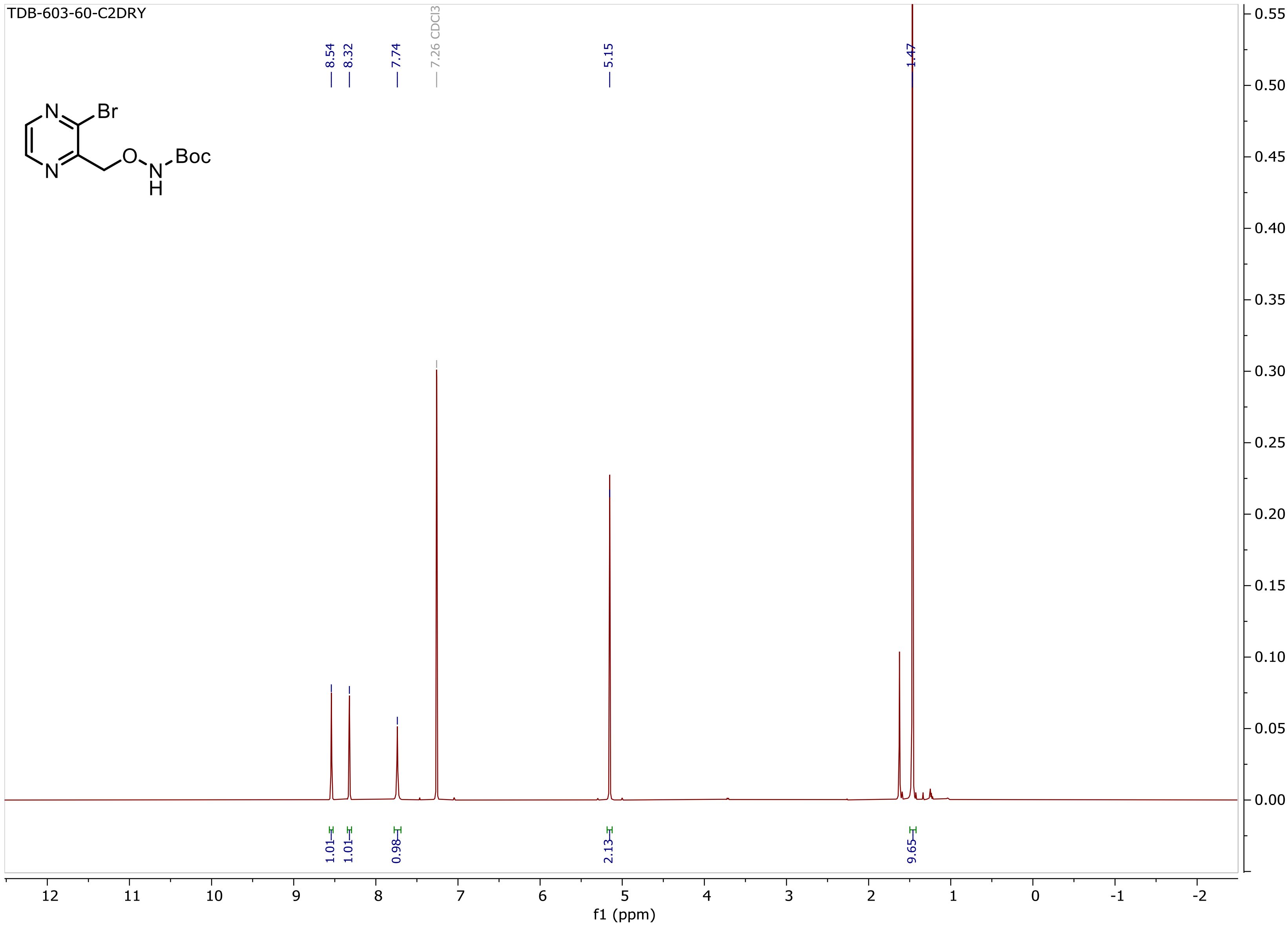

^{13}C NMR (126 MHz, CDCl$_3$) spectrum of *tert*-butyl ((3-bromopyrazin-2-yl)methoxy)carbamate (**S3**):

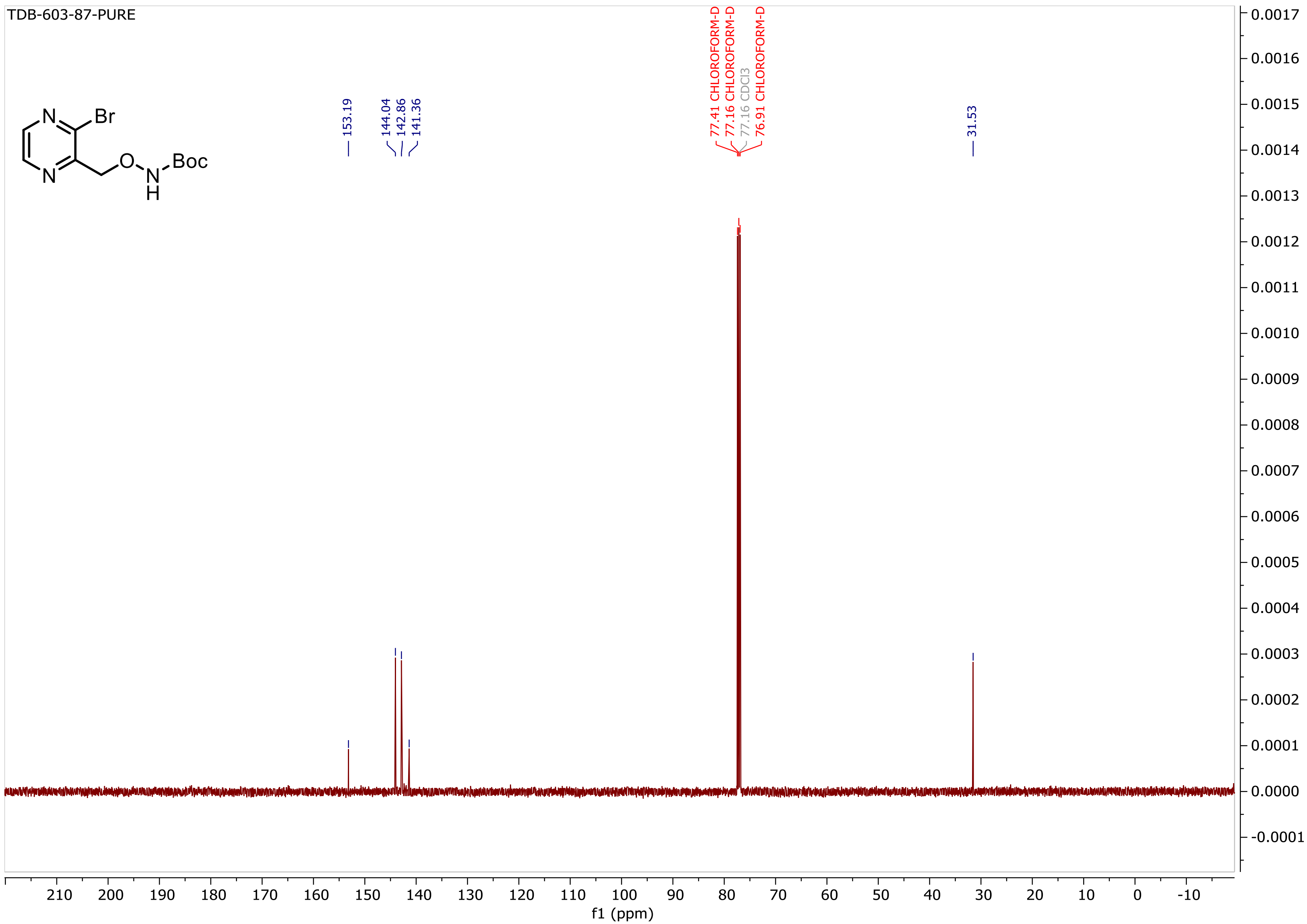

^{1}H NMR (500 MHz, CDCl$_3$) spectrum of *tert*-butyl isoxazolo[3,4-*b*]pyrazine-1(3*H*)-carboxylate (**25**):

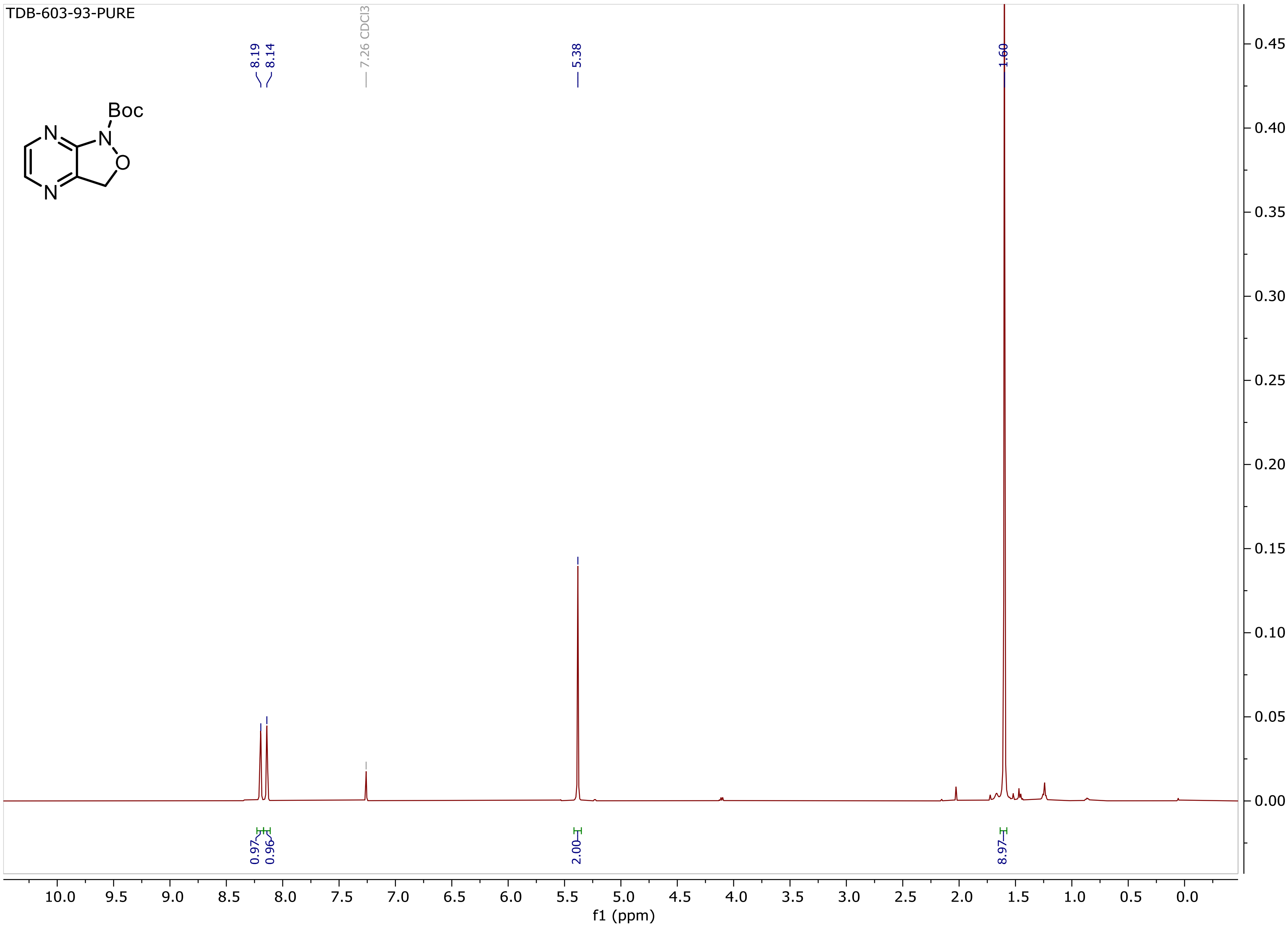

^{13}C NMR (126 MHz, $CDCl_3$) spectrum of *tert*-butyl isoxazolo[3,4-*b*]pyrazine-1(3*H*)-carboxylate (**25**):

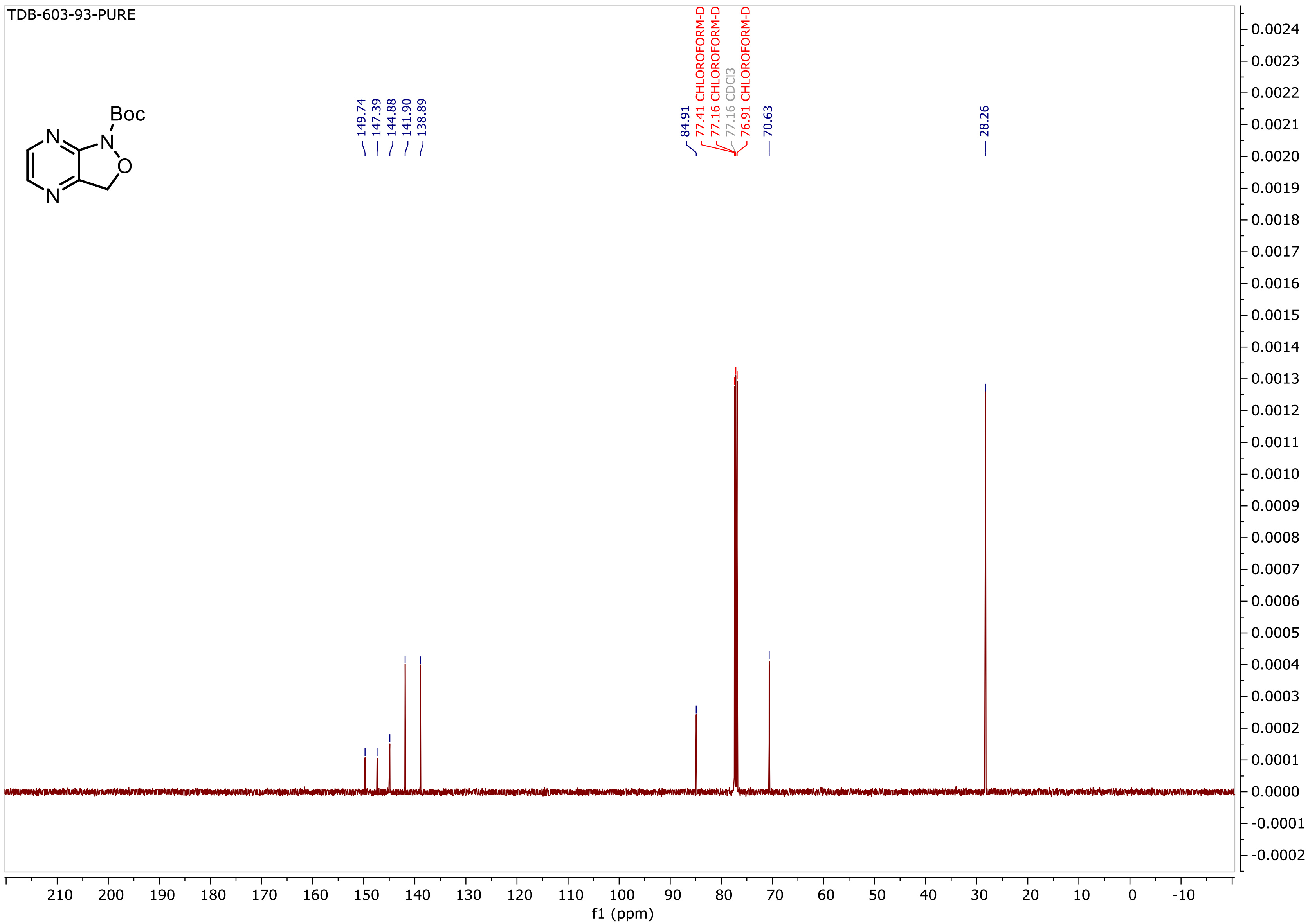

^{1}H NMR (500 MHz, CDCl₃) spectrum of *tert*-butyl (2-bromophenethoxy)carbamate (**S4**):

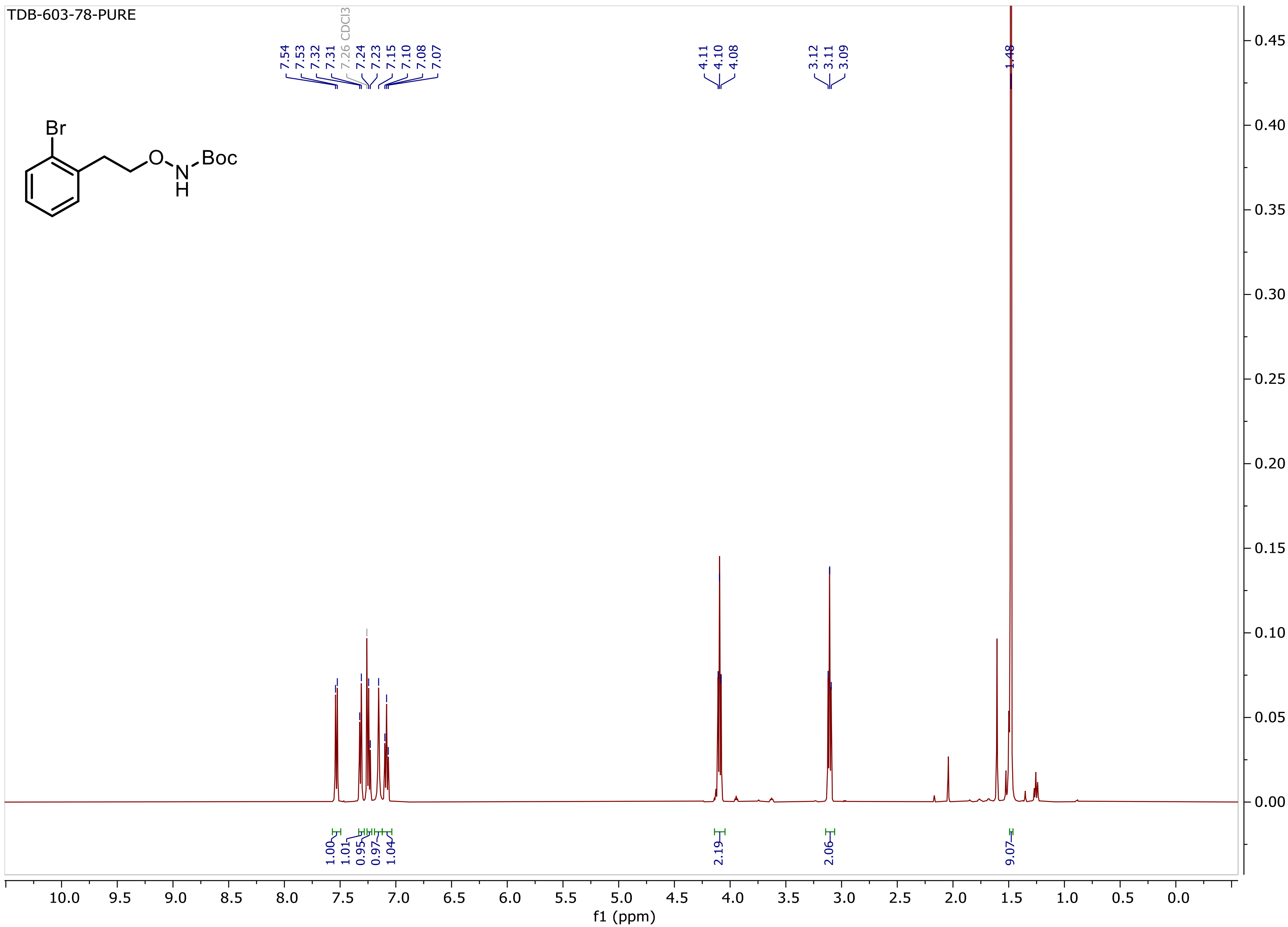

^{13}C NMR (126 MHz, CDCl$_3$) spectrum of *tert*-butyl (2-bromophenethoxy)carbamate (**S4**):

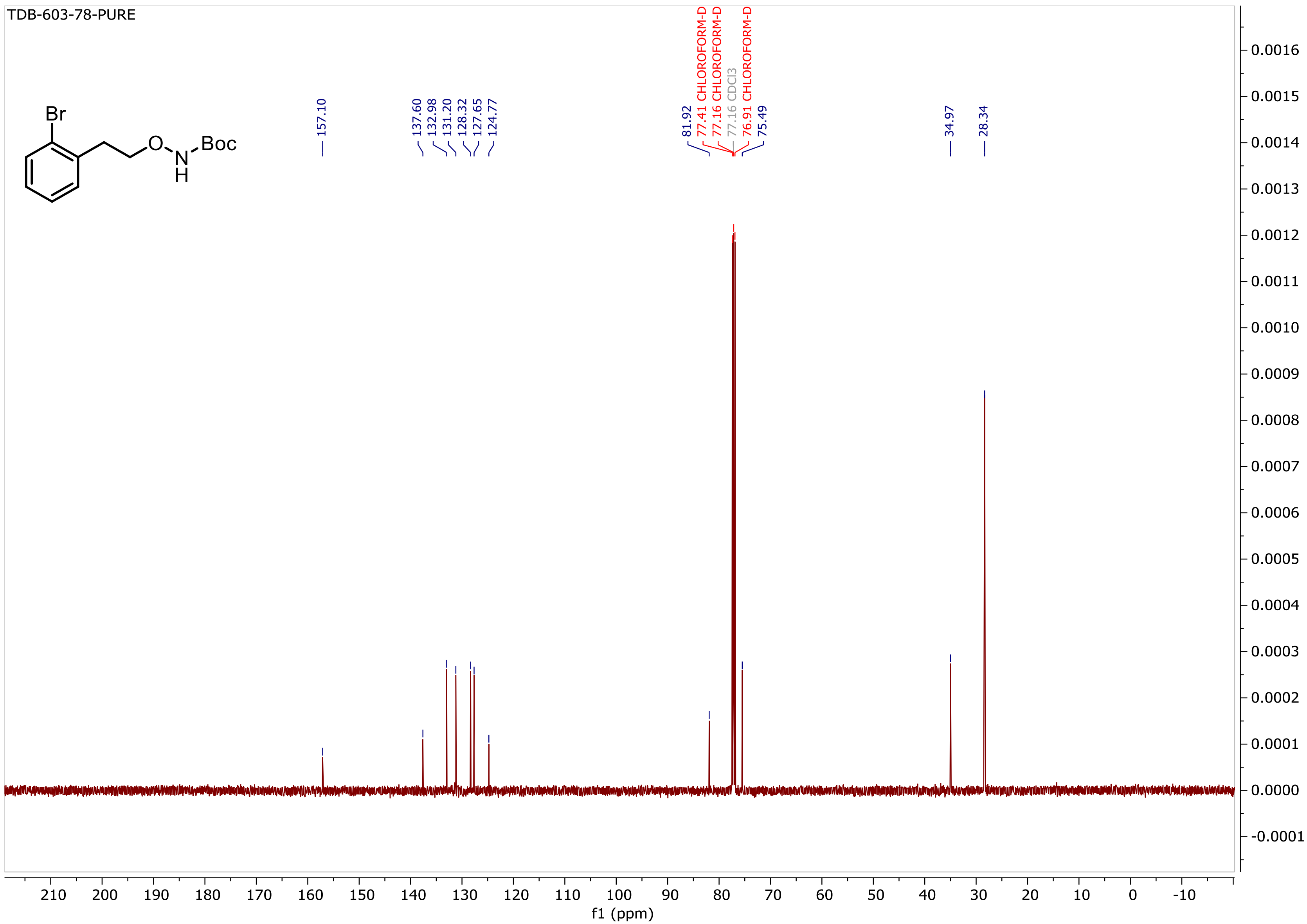

^{1}H NMR (500 MHz, CDCl3) spectrum of *tert*-butyl 3,4-dihydro-1*H*-benzo[*c*][1,2]oxazine-1-carboxylate (**26**):

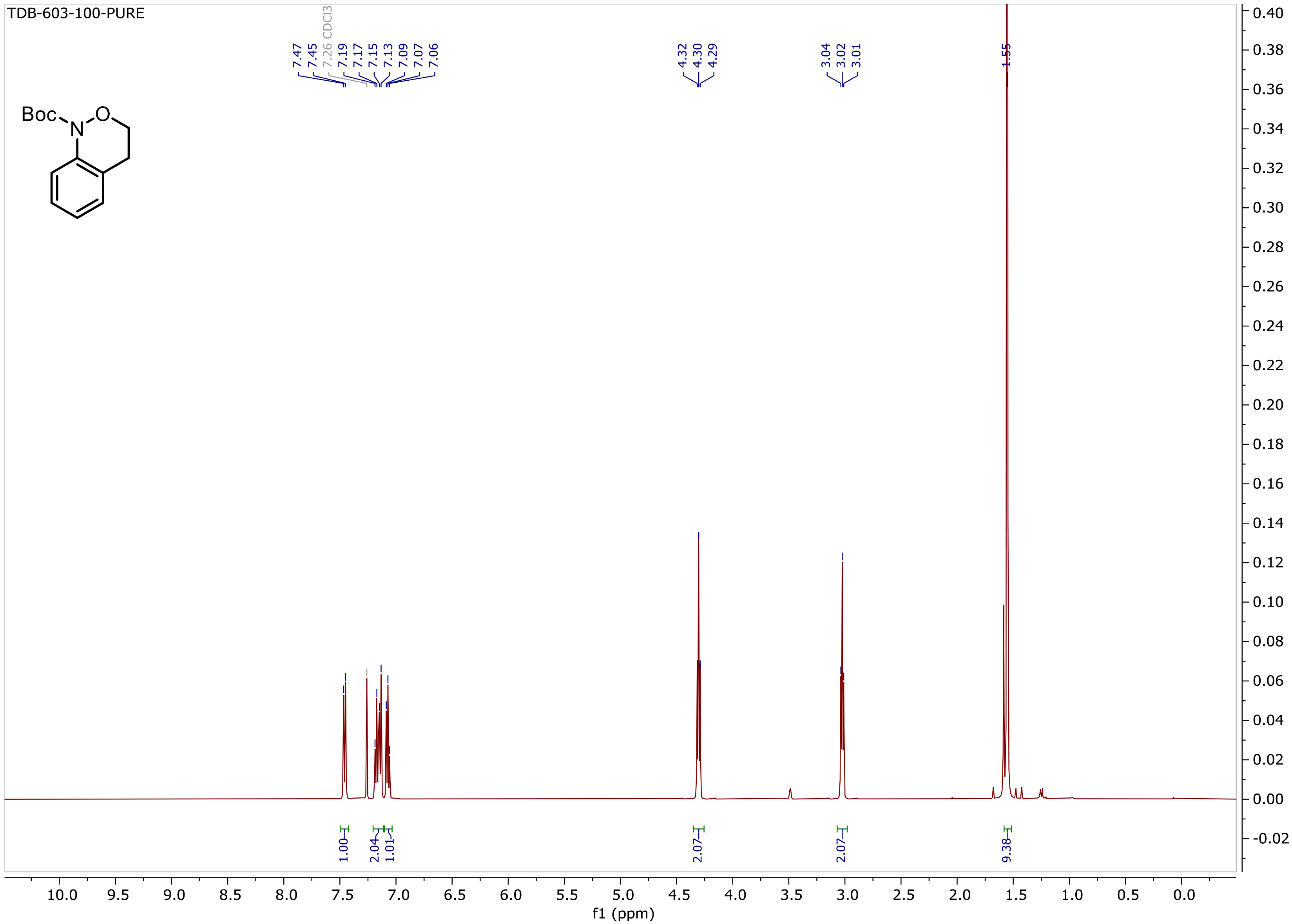

^{13}C NMR (126 MHz, CDCl$_3$) spectrum of *tert*-butyl 3,4-dihydro-1*H*-benzo[*c*][1,2]oxazine-1-carboxylate (**26**):

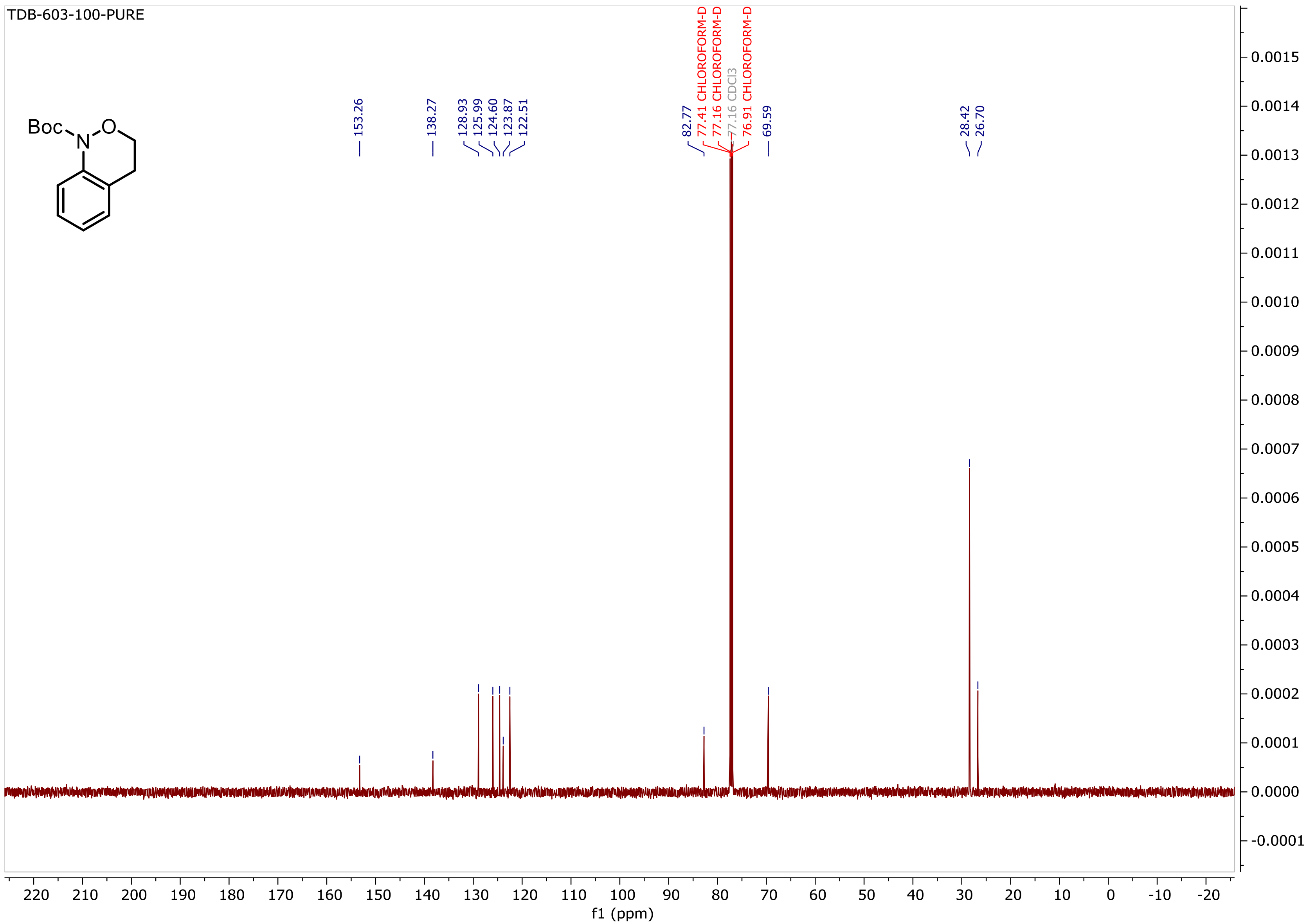

^{1}**H NMR (500 MHz, CDCl$_3$)** spectrum of *tert*-butyl (3-(2-bromophenyl)propoxy)carbamate (**S5**):

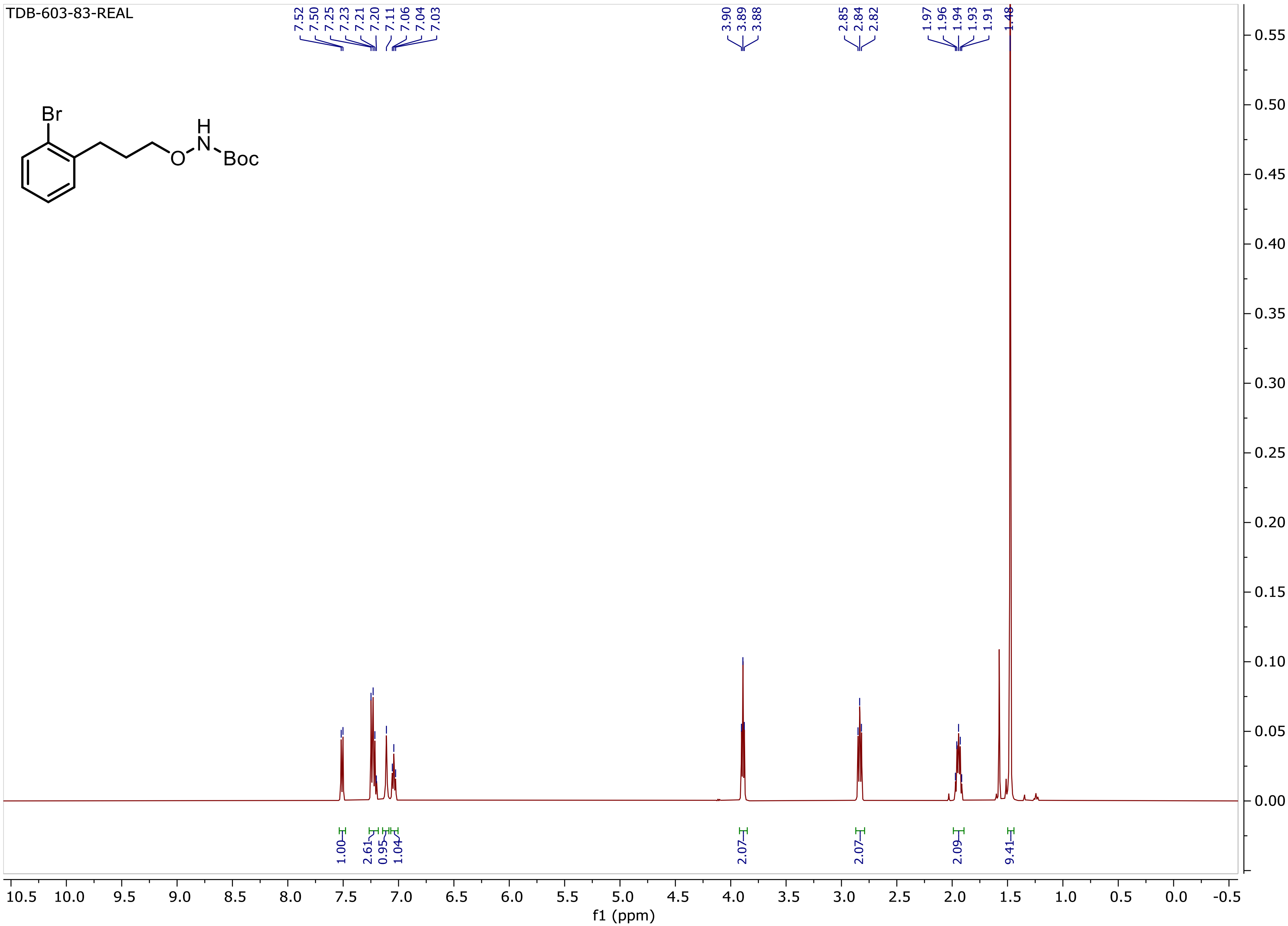

^{13}C NMR (126 MHz, CDCl$_3$) spectrum of *tert*-butyl (3-(2-bromophenyl)propoxy)carbamate (**S5**):

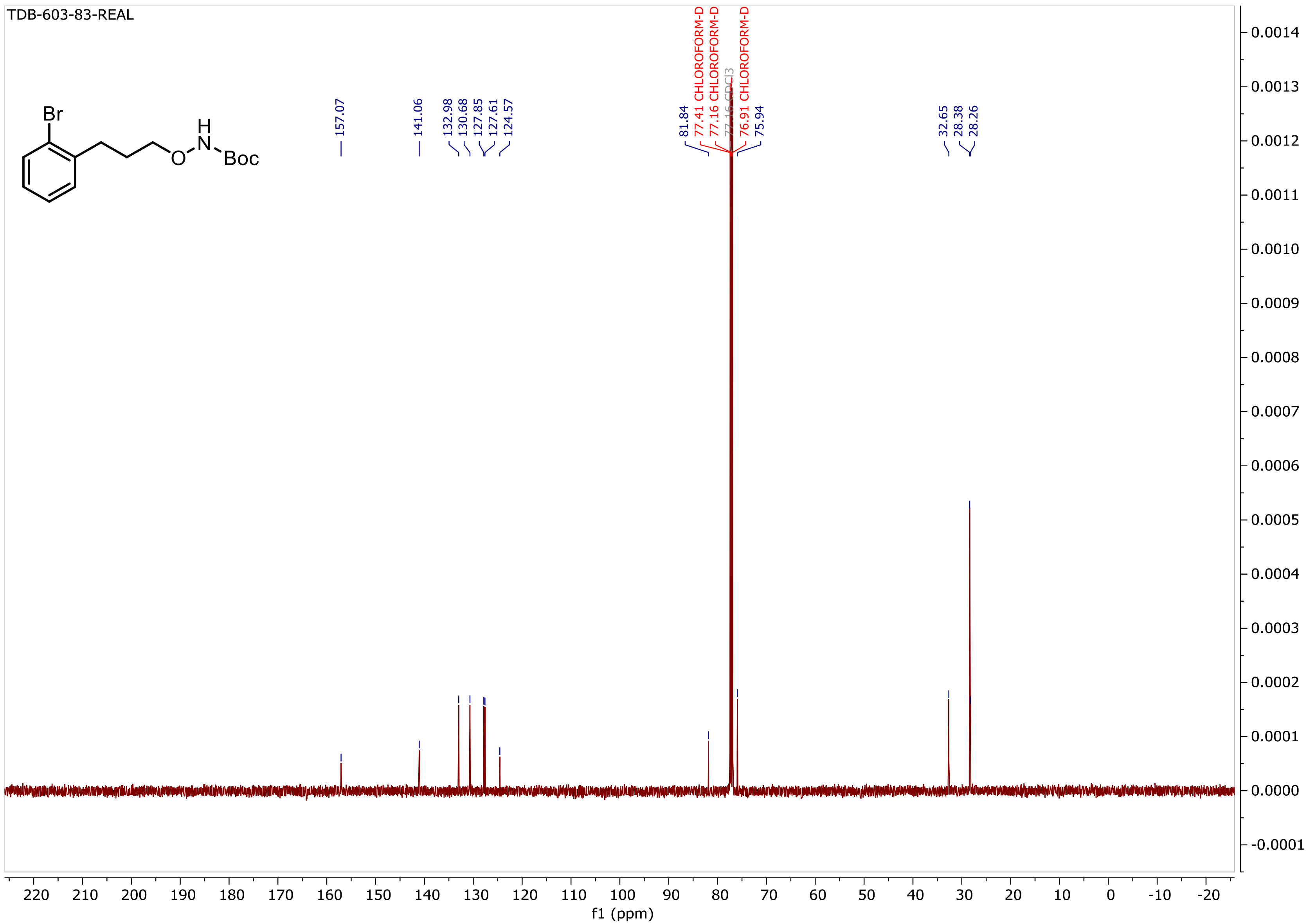

^{1}H NMR (500 MHz, $CDCl_3$) spectrum of *tert*-butyl 4,5-dihydrobenzo[*c*][1,2]oxazepine-1(3*H*)-carboxylate (**27**):

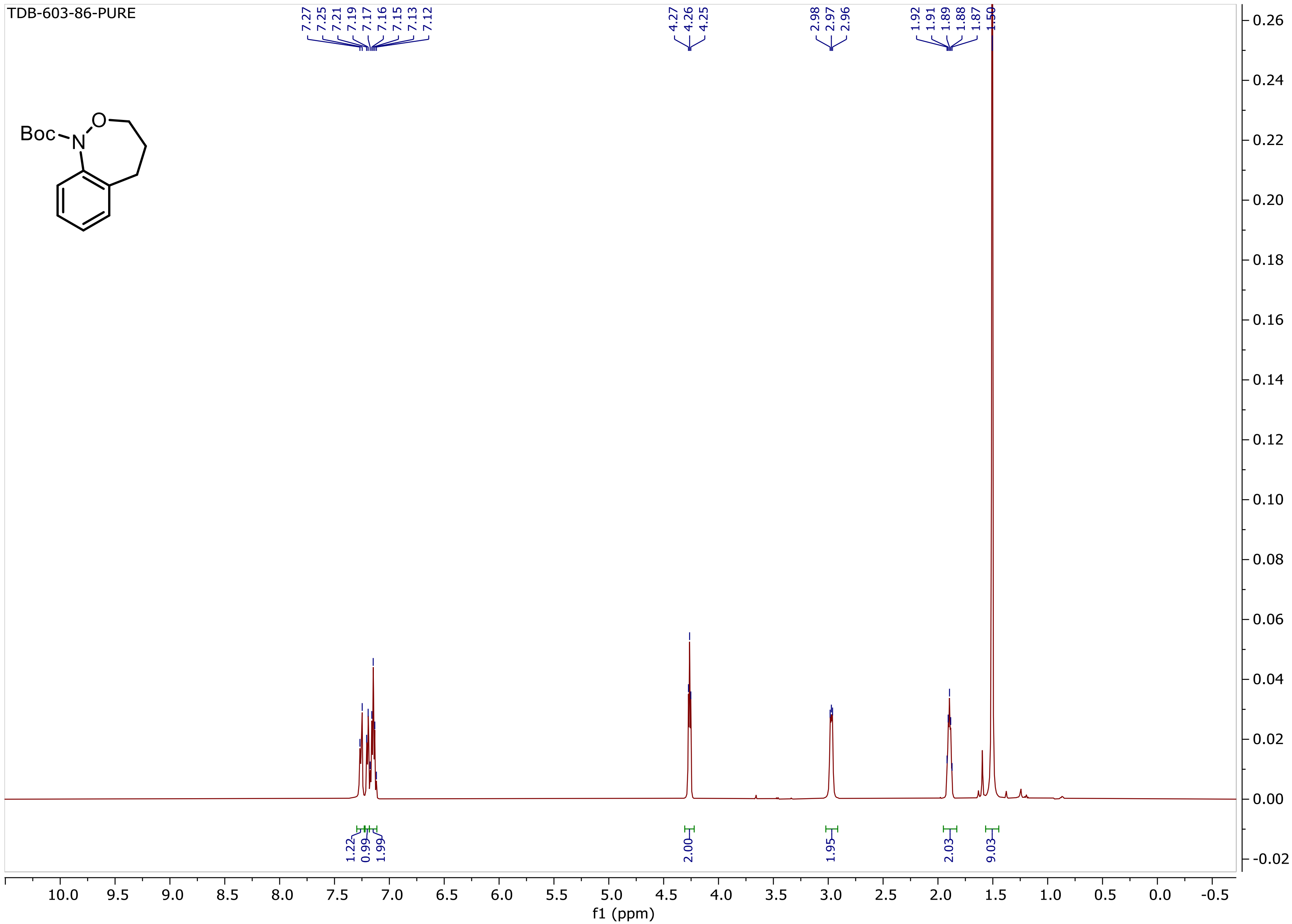

^{13}C NMR (126 MHz, $CDCl_3$) spectrum of *tert*-butyl 4,5-dihydrobenzo[*c*][1,2]oxazepine-1(3*H*)-carboxylate (**27**):

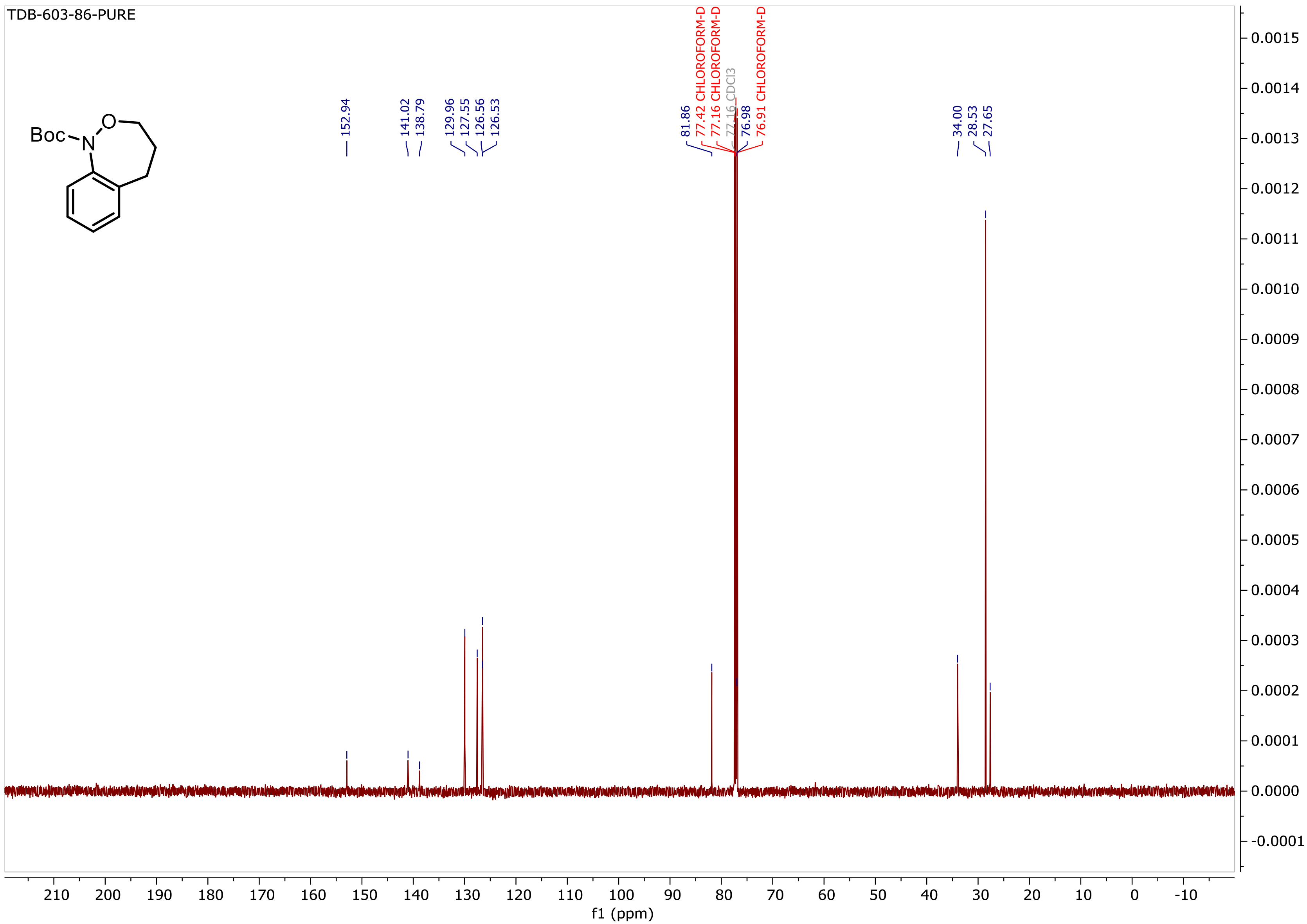

^{1}H NMR (500 MHz, CDCl$_3$) spectrum of *tert*-butyl 4-(benzo[*c*]isoxazol-1(3*H*)-ylmethyl)piperidine-1-carboxylate (**31**):

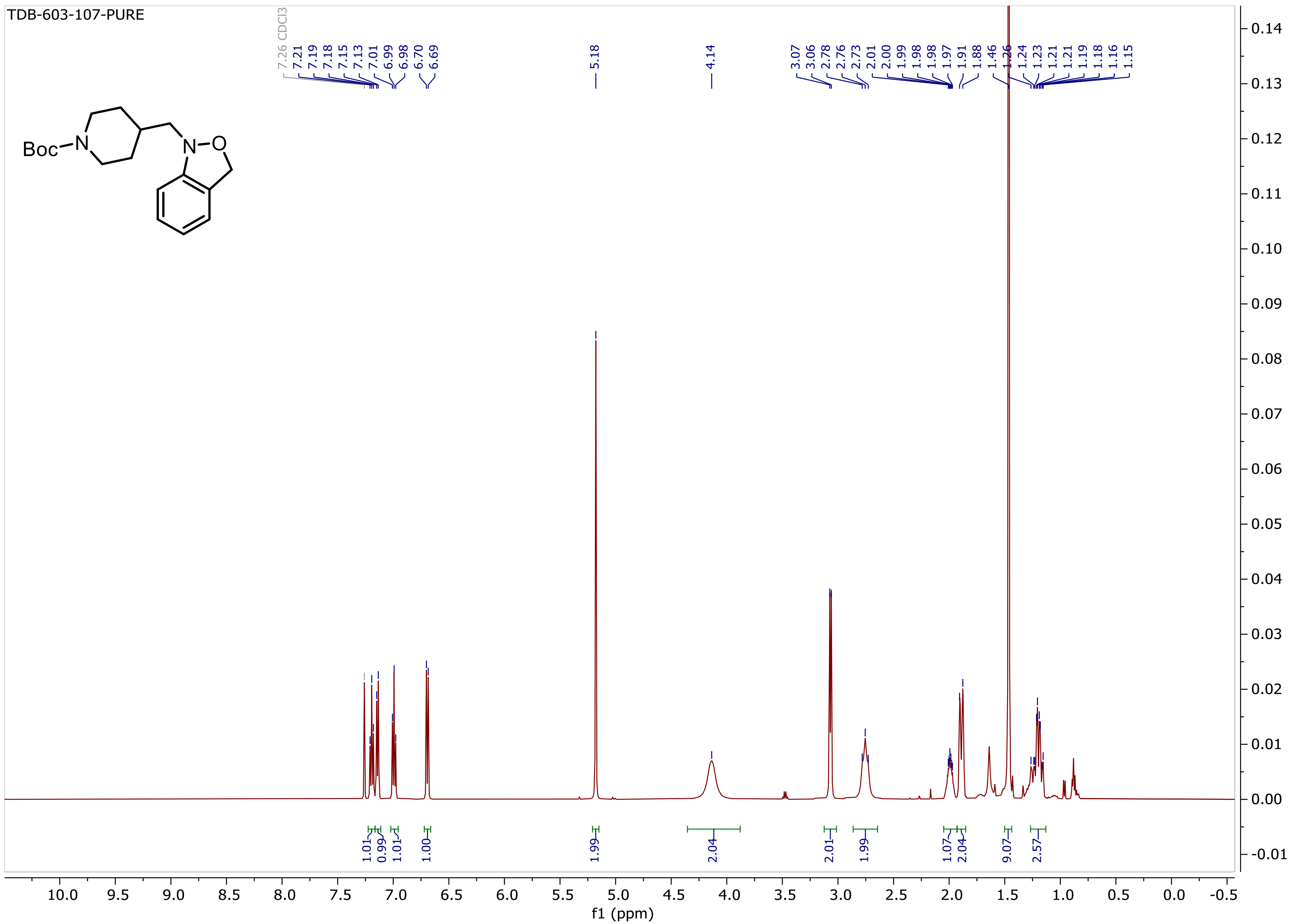

^{13}C NMR (126 MHz, CDCl$_3$) spectrum of *tert*-butyl 4-(benzo[*c*]isoxazol-1(3*H*)-ylmethyl)piperidine-1-carboxylate (**31**):

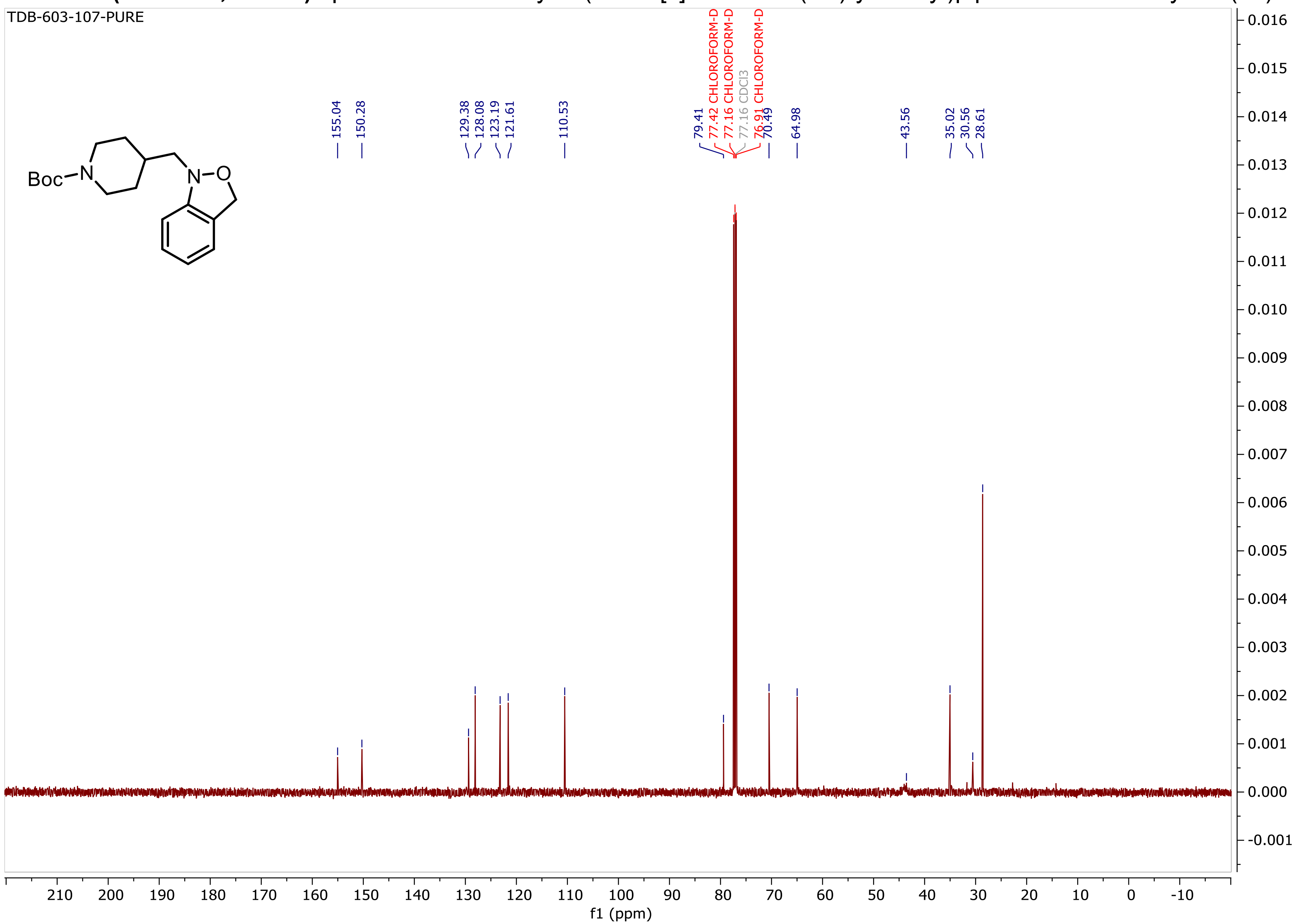

^{1}H NMR (500 MHz, $CDCl_3$) spectrum of *tert*-butyl (2-(7-methylbenzo[*c*]isoxazol-1(3*H*)-yl)ethyl)carbamate (**33**):

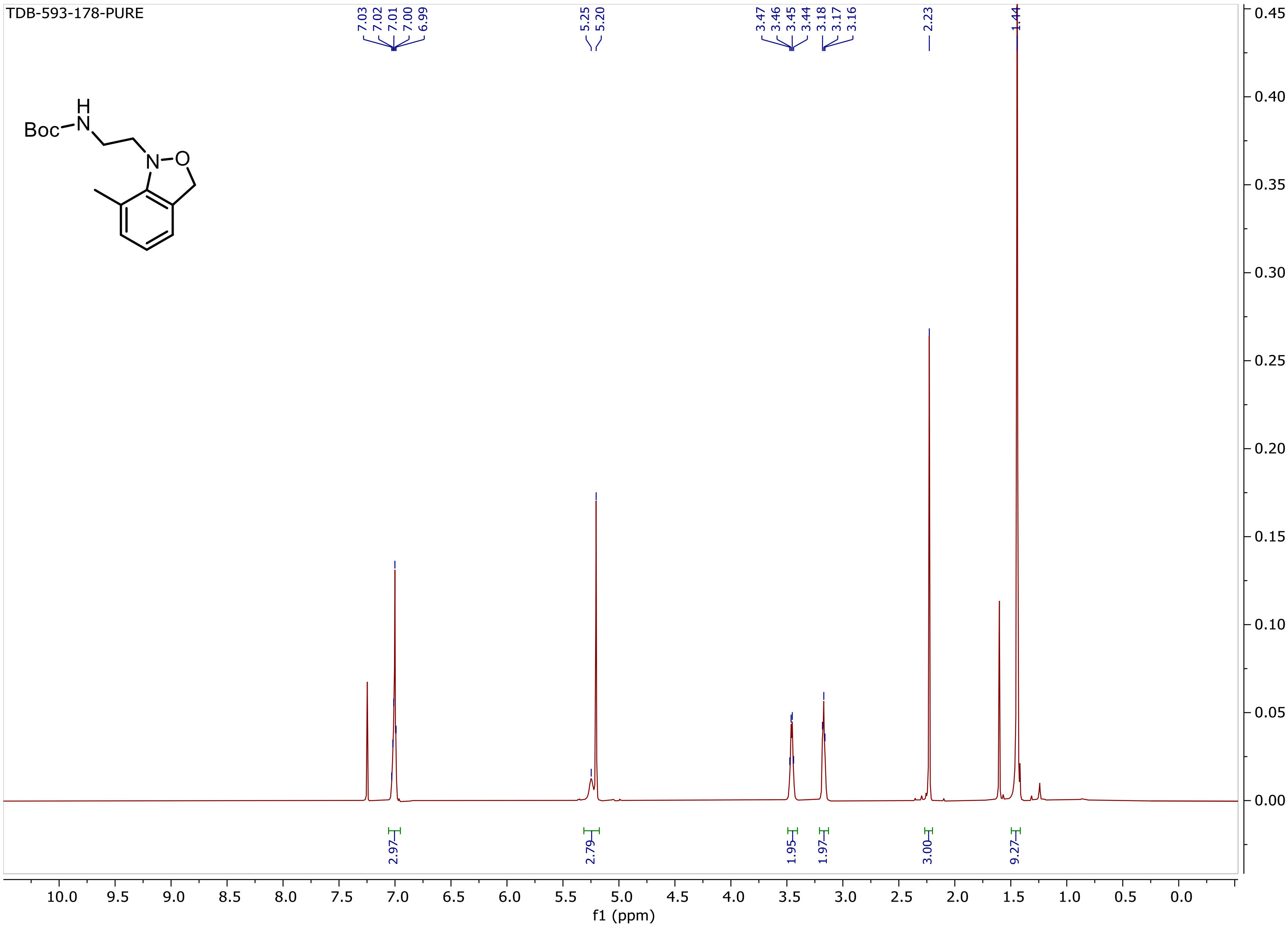

^{13}C NMR (126 MHz, CDCl$_3$) spectrum of *tert*-butyl (2-(7-methylbenzo[*c*]isoxazol-1(3*H*)-yl)ethyl)carbamate (**33**):

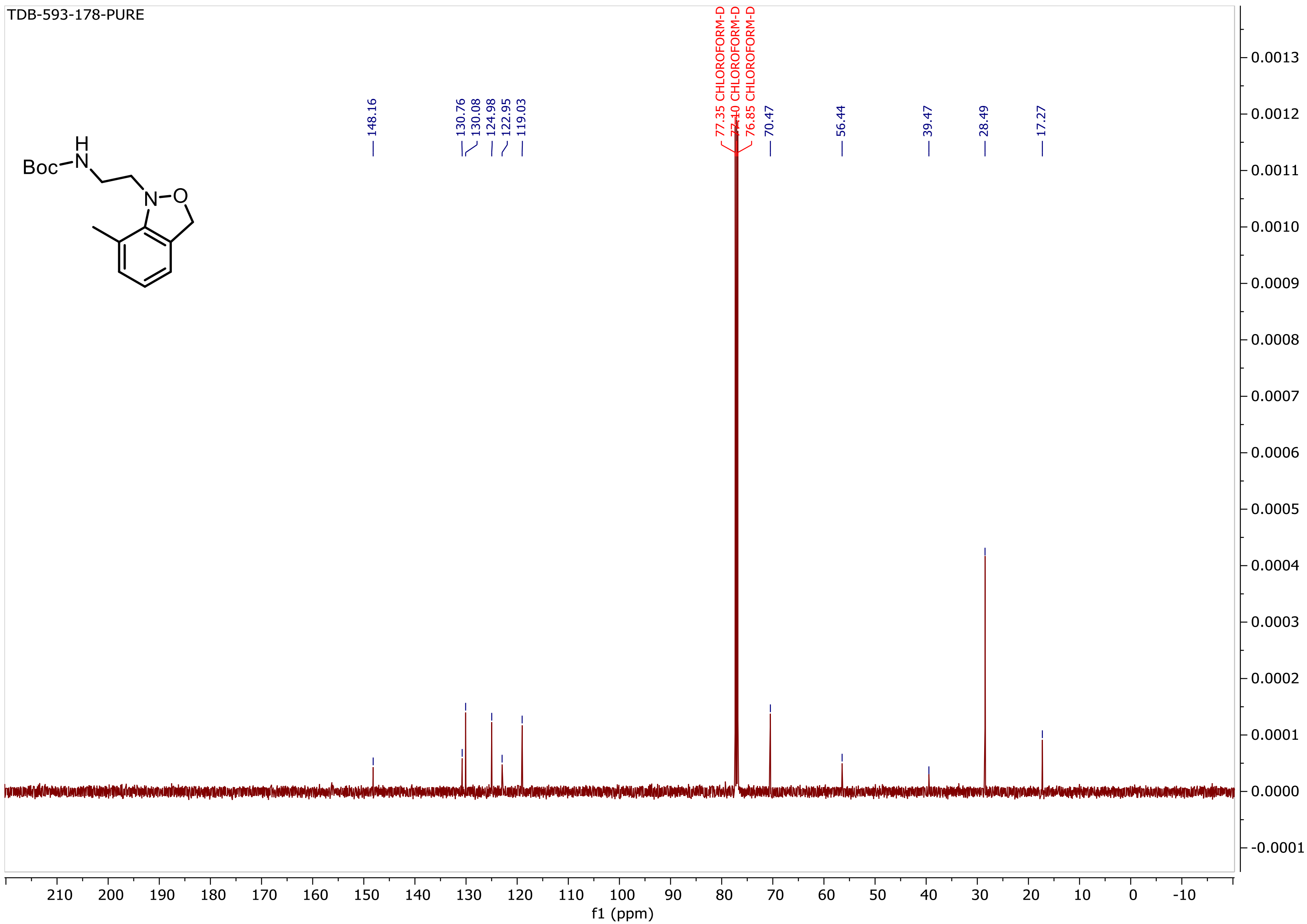

^{1}H NMR (500 MHz, CDCl$_3$) spectrum of 1-(cyclopropylmethyl)-1,3-dihydroisoxazolo[4,3-*b*]pyridine (**35**):

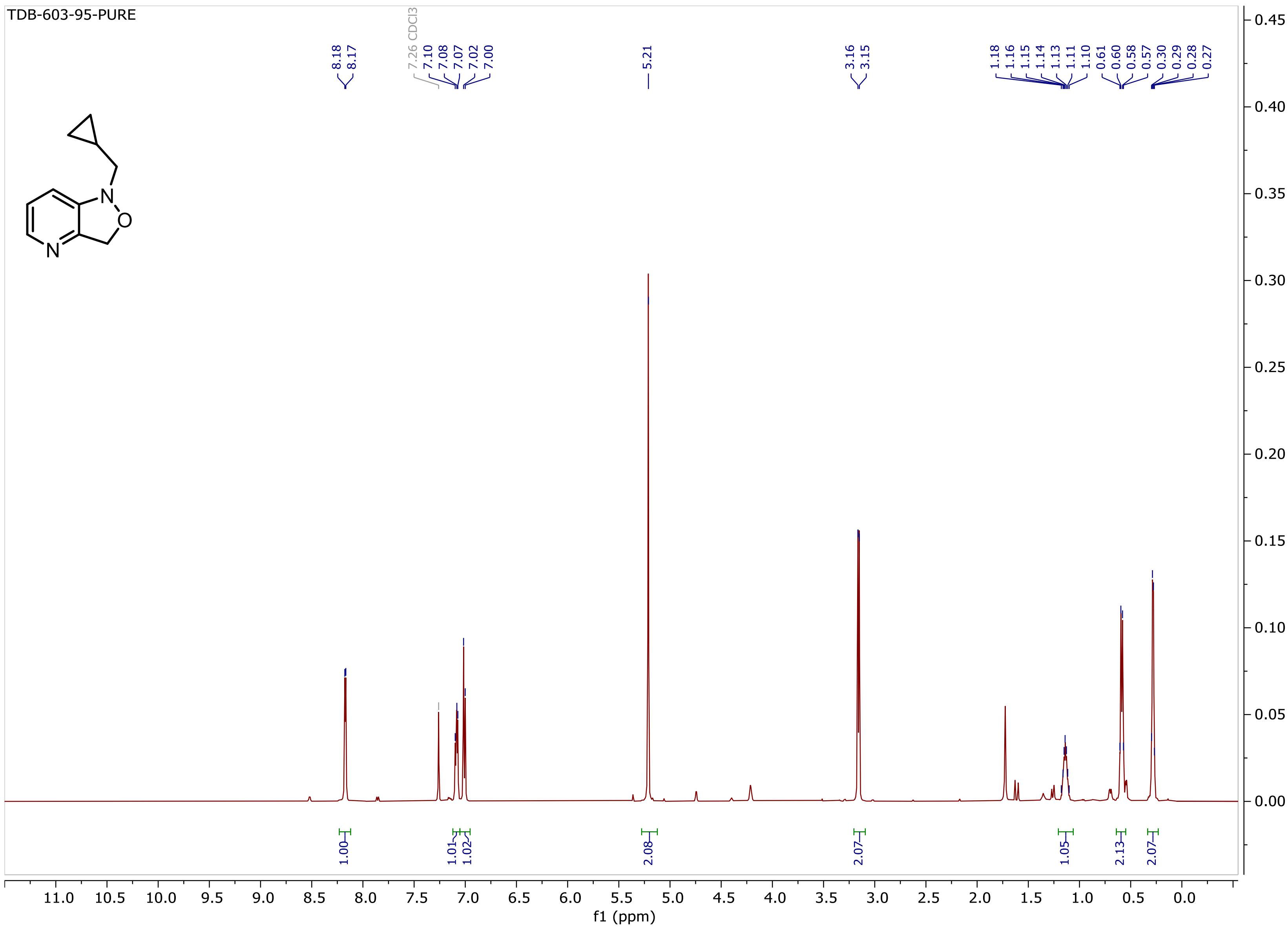

^{13}C NMR (126 MHz, CDCl$_3$) spectrum of 1-(cyclopropylmethyl)-1,3-dihydroisoxazolo[4,3-*b*]pyridine (**35**):

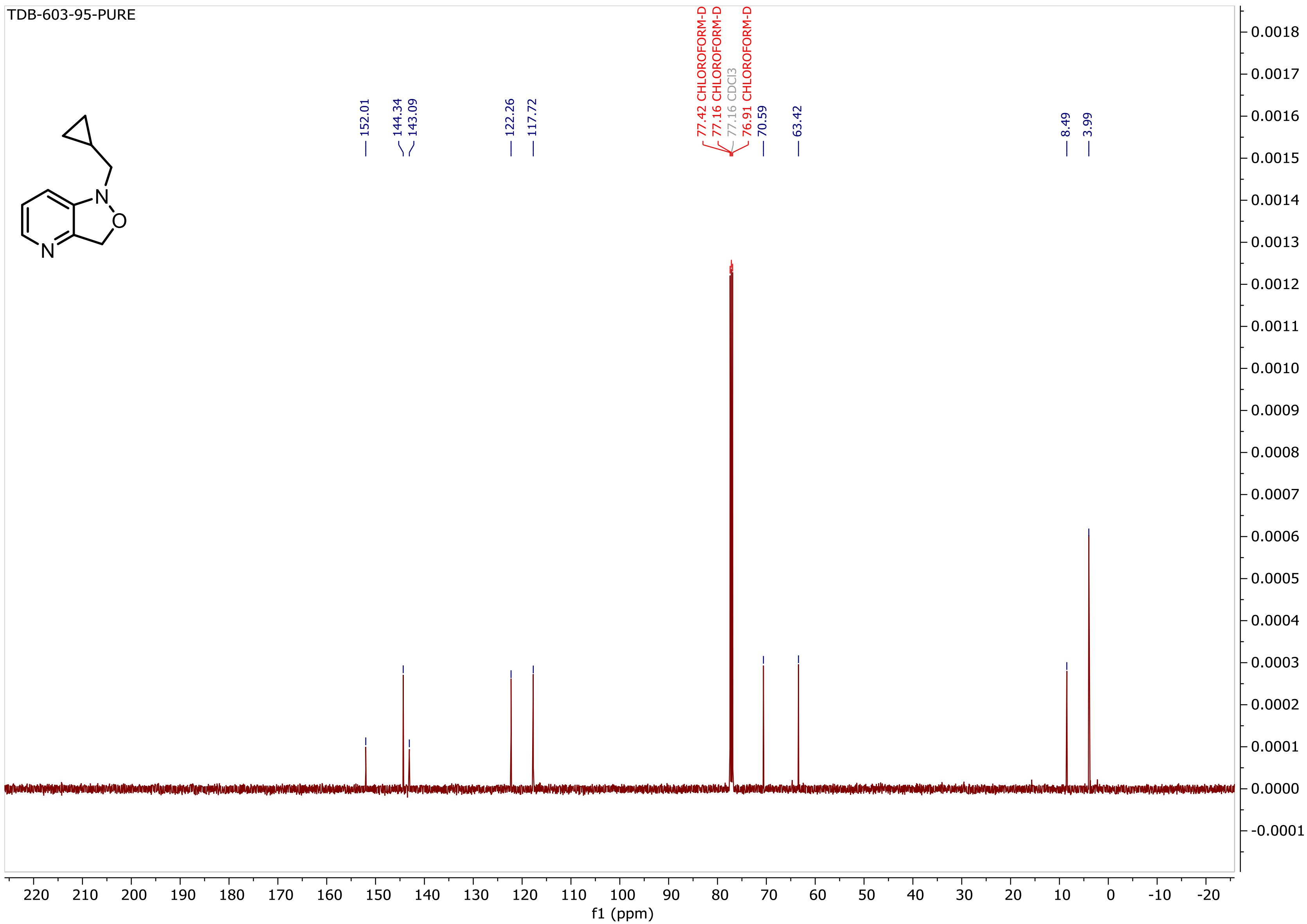

^{1}**H NMR (500 MHz, CDCl$_3$)** spectrum of *tert*-butyl (2,3-dihydrobenzofuran-4-yl)carbamate (**37**):

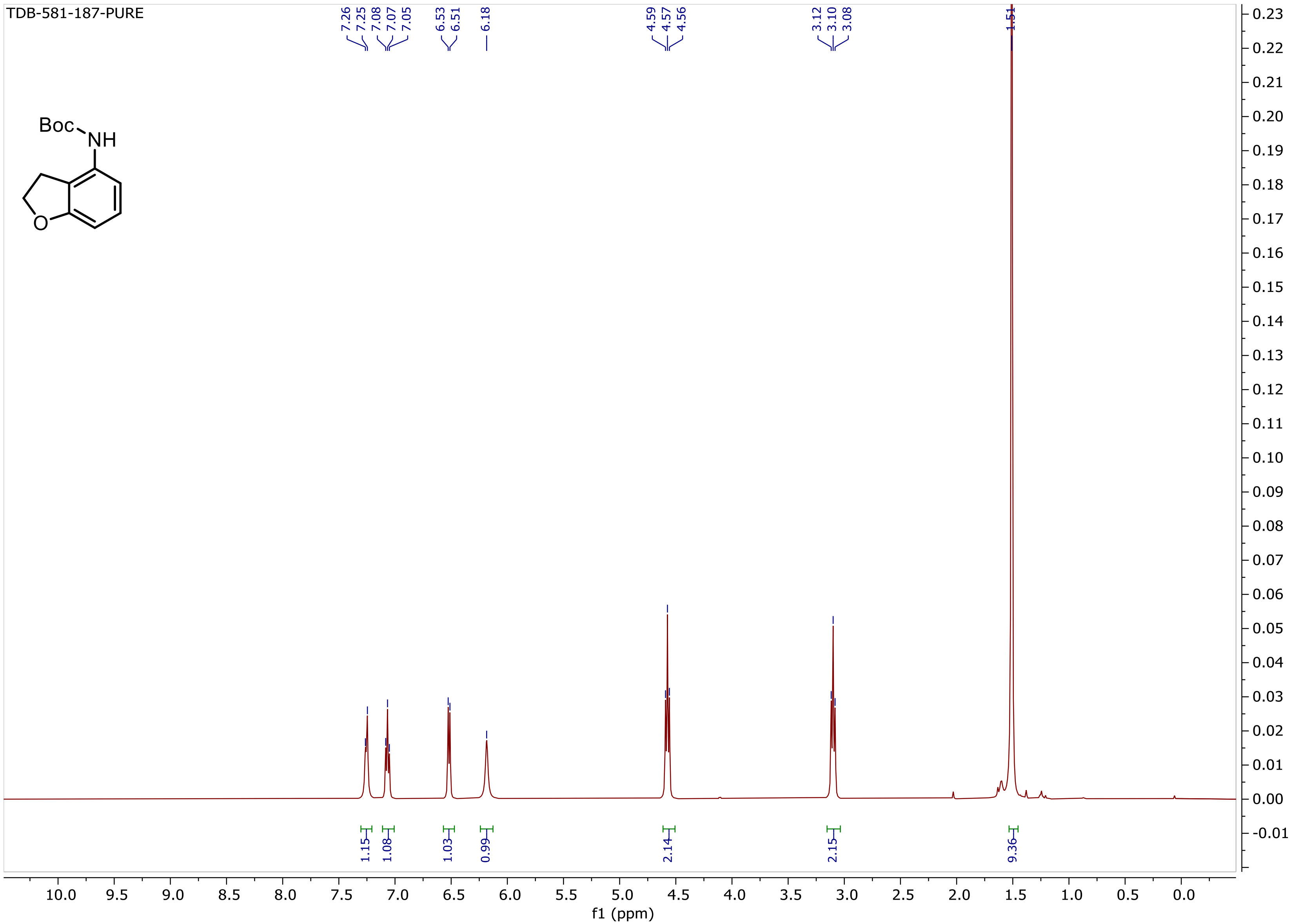

^{13}C NMR (126 MHz, CDCl$_3$) spectrum of *tert*-butyl (2,3-dihydrobenzofuran-4-yl)carbamate (**37**):

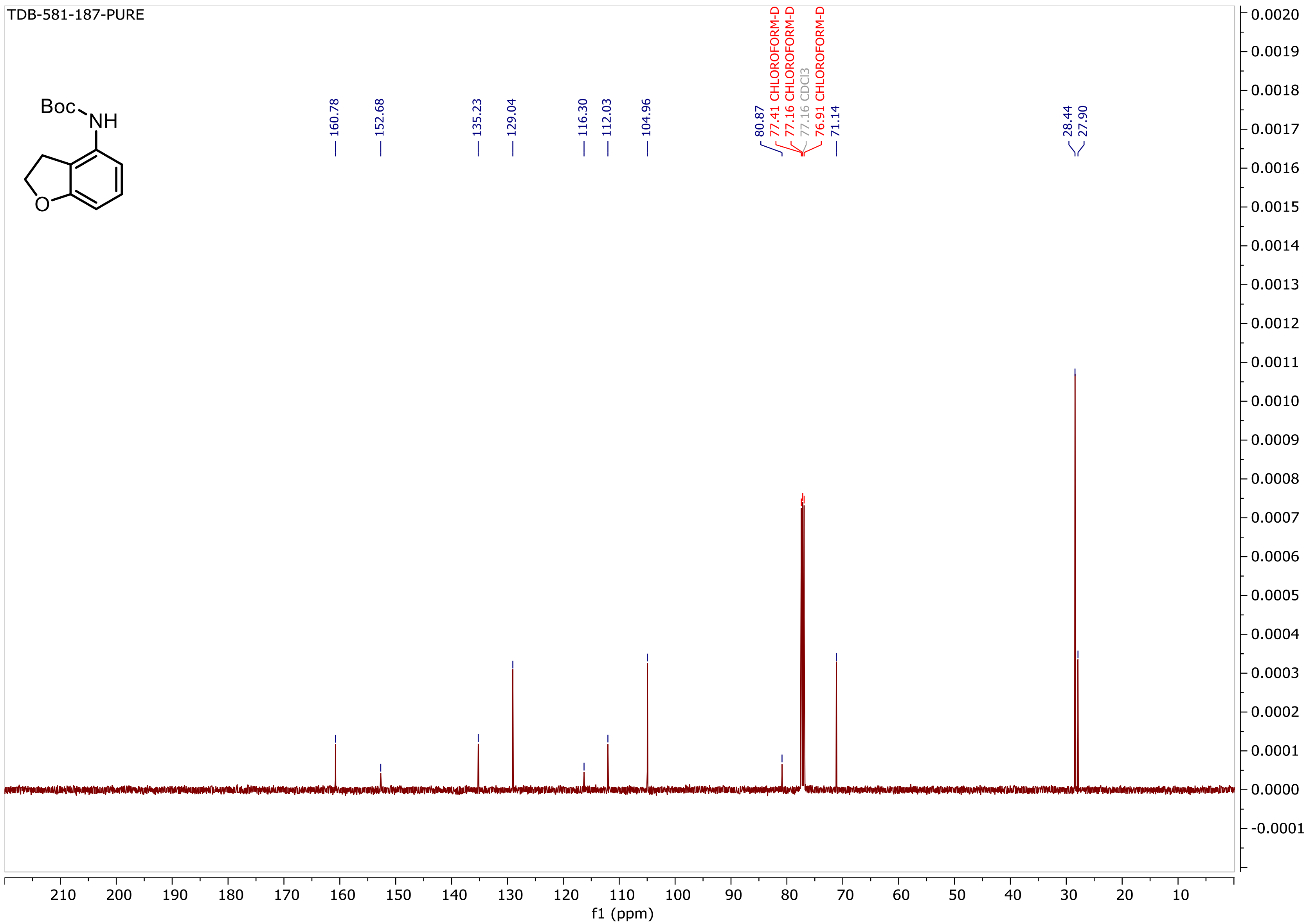

^{1}H NMR (500 MHz, CDCl$_3$) spectrum of *tert*-butyl (5-bromo-2,3-dihydrobenzofuran-4-yl)carbamate (**38**):

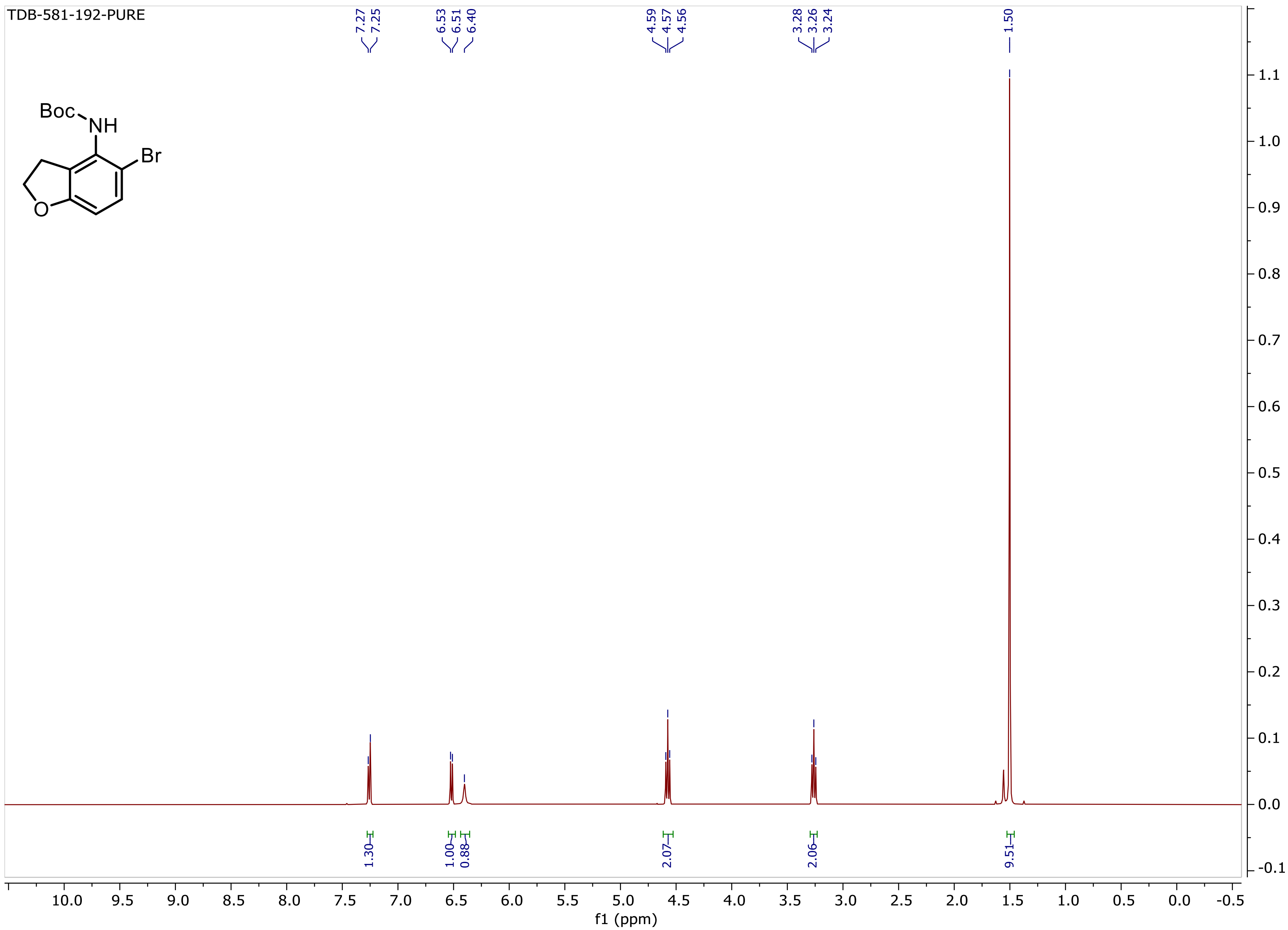

^{13}C NMR (126 MHz, CDCl$_3$) spectrum of *tert*-butyl (5-bromo-2,3-dihydrobenzofuran-4-yl)carbamate (**38**):

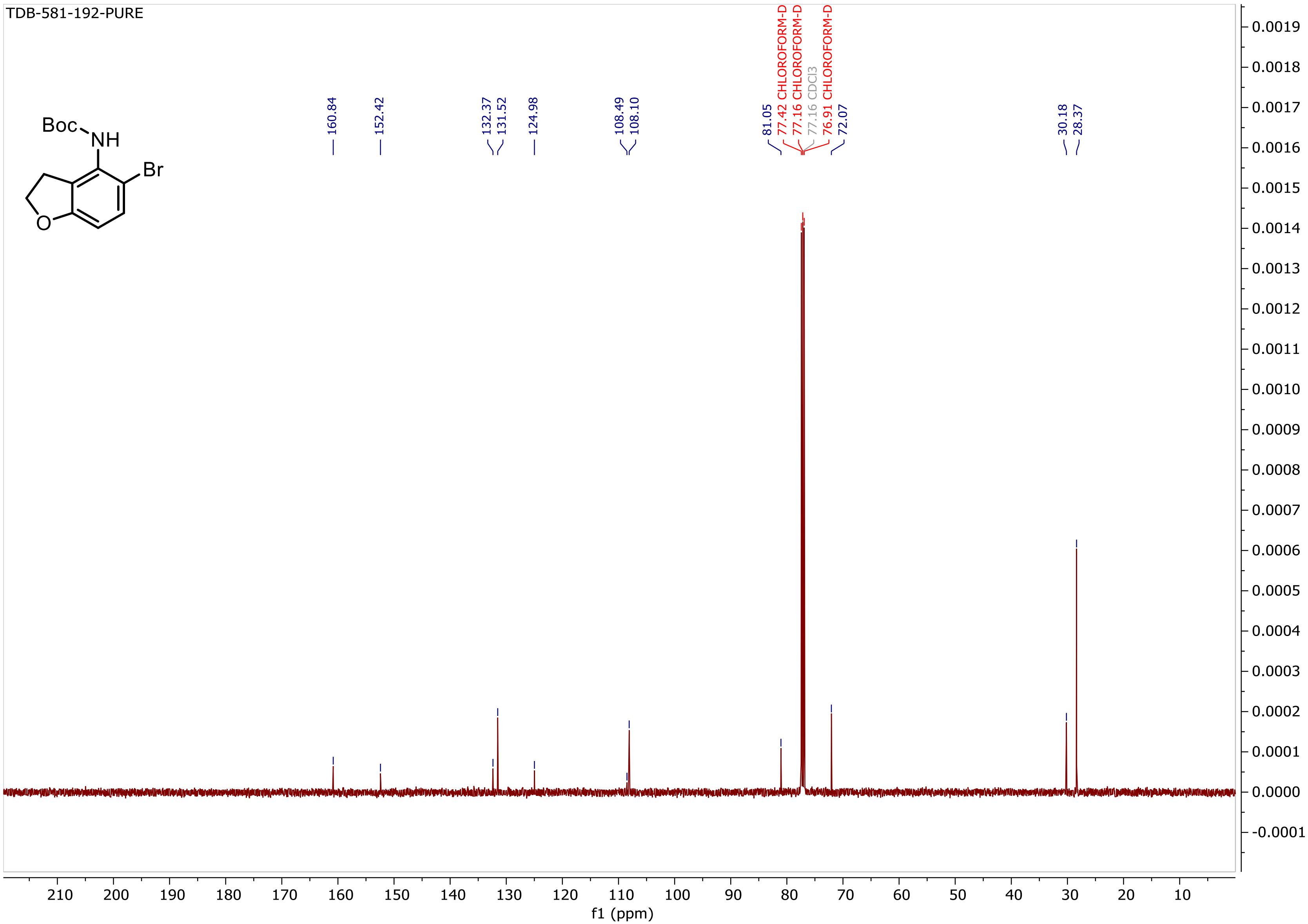

^{1}H NMR (500 MHz, CDCl$_3$) spectrum of *tert*-butyl (5-formyl-2,3-dihydrobenzofuran-4-yl)carbamate (**39**):

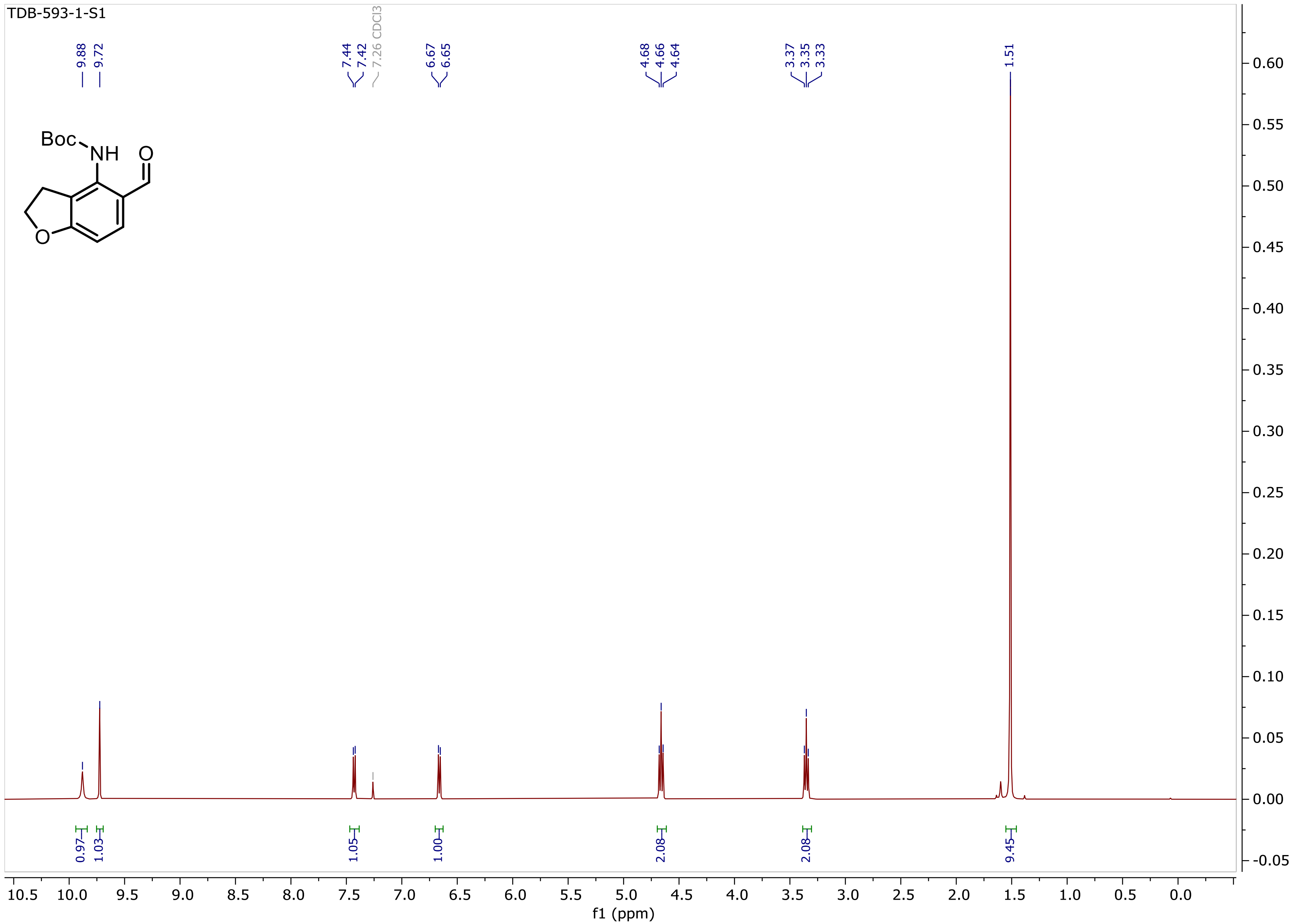

^{13}**C NMR (126 MHz, CDCl$_3$)** spectrum of *tert*-butyl (5-formyl-2,3-dihydrobenzofuran-4-yl)carbamate (**39**):

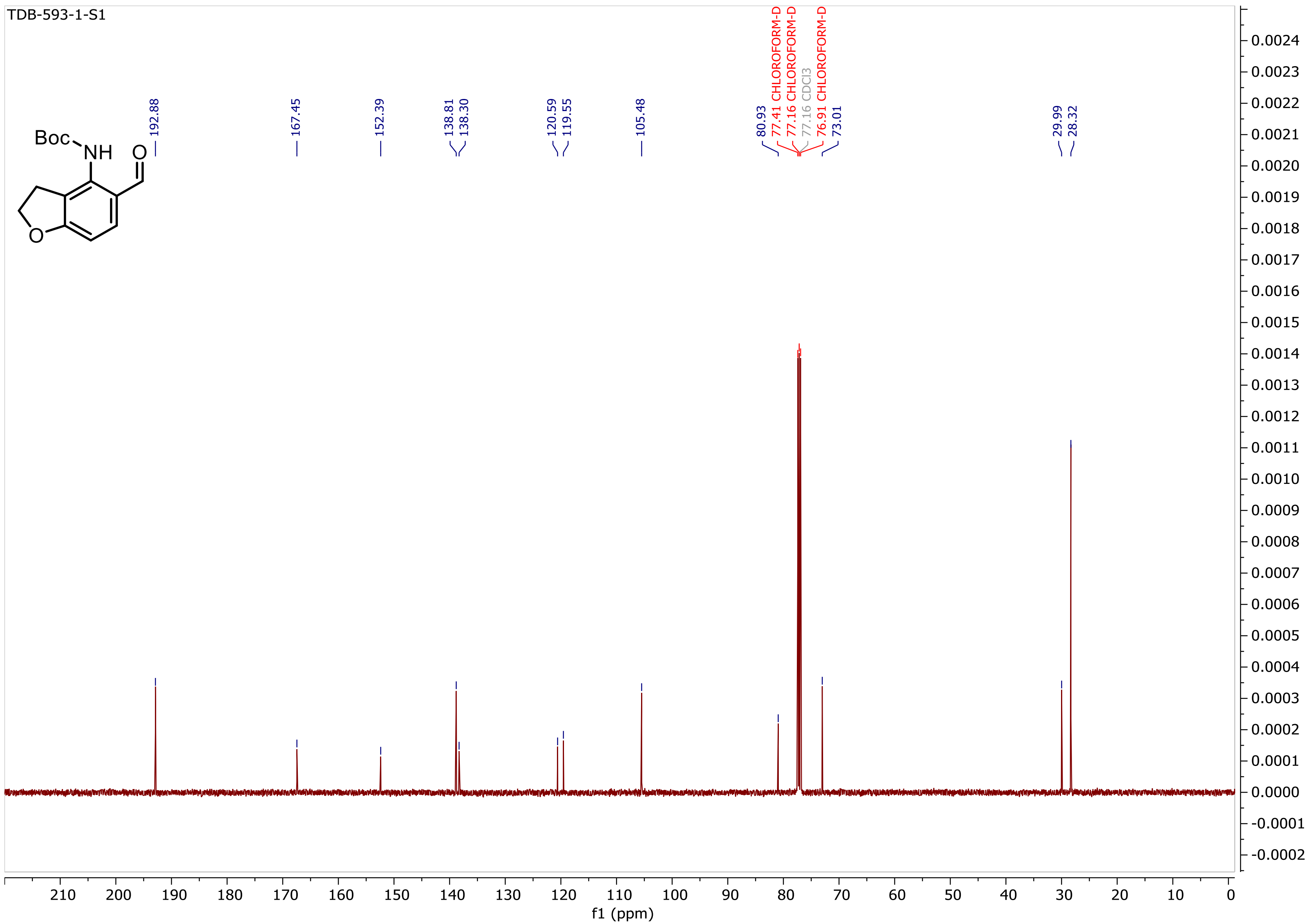

^{1}H NMR (500 MHz, $CDCl_3$) spectrum of 4-amino-2,3-dihydrobenzofuran-5-carbaldehyde (**40**):

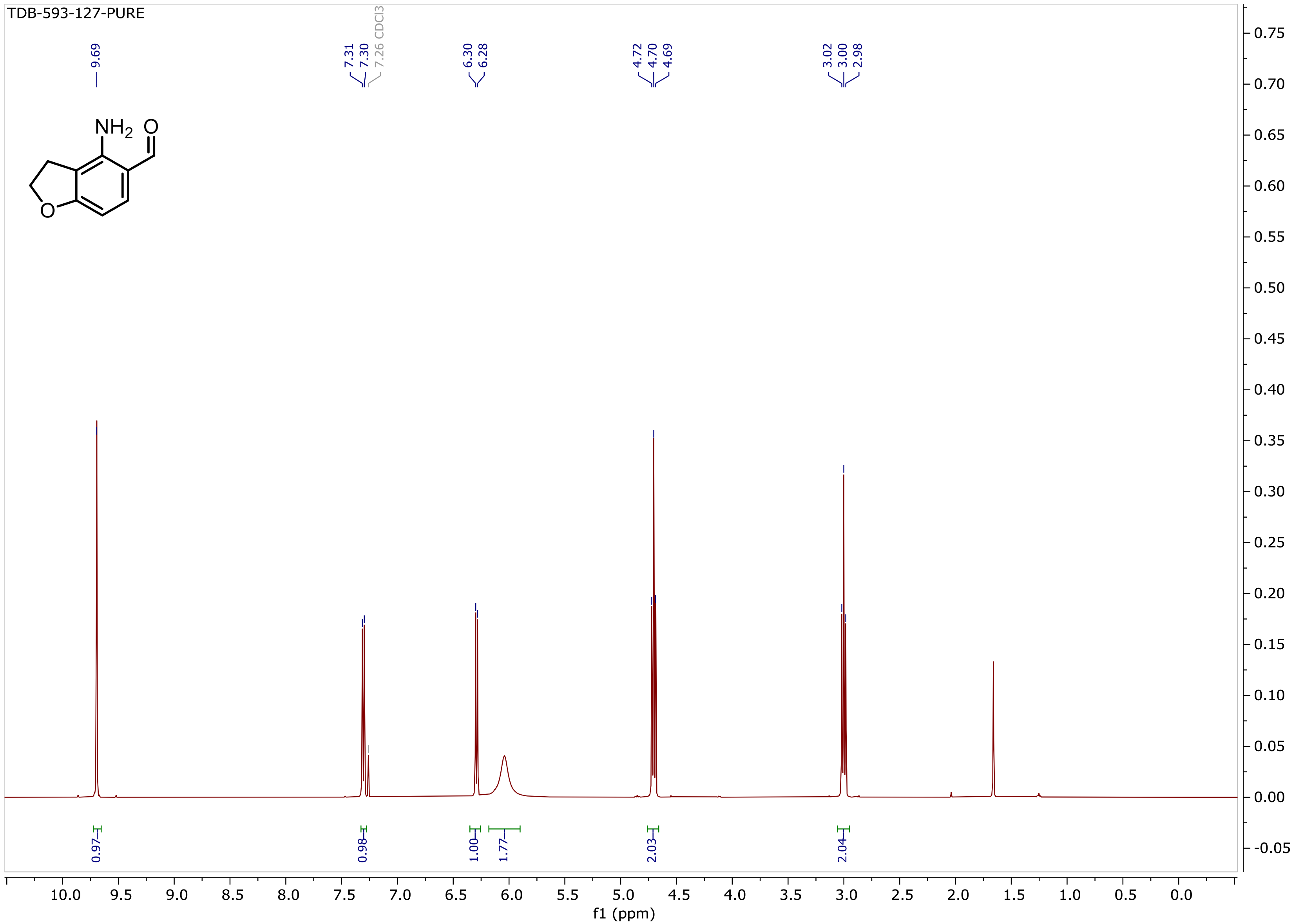

^{13}**C NMR (126 MHz, CDCl$_3$)** spectrum of 4-amino-2,3-dihydrobenzofuran-5-carbaldehyde (**40**):

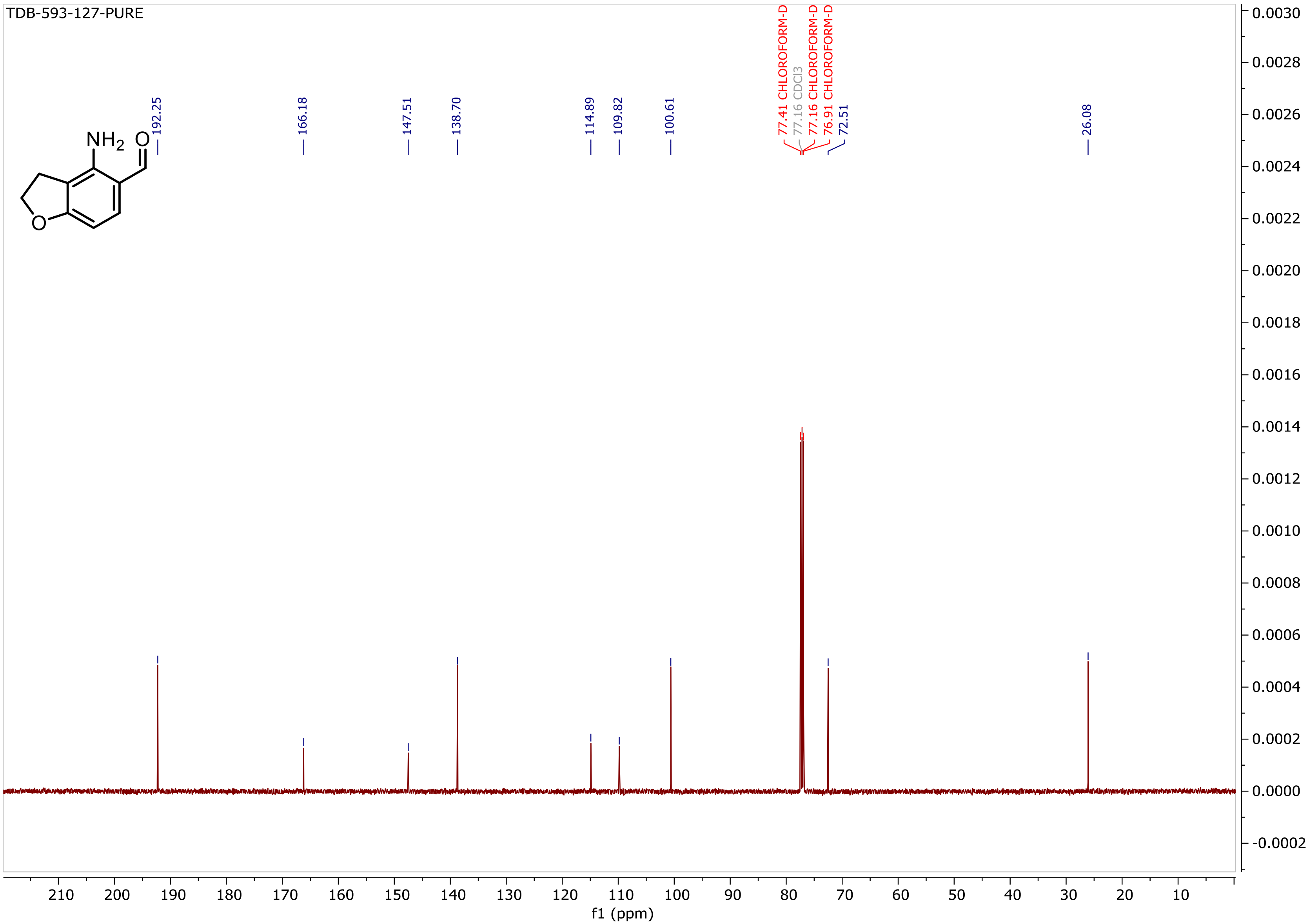

^{1}H NMR (500 MHz, CDCl$_3$) spectrum of 4-bromo-2,3-dihydrobenzofuran-5-carbaldehyde (**41**):

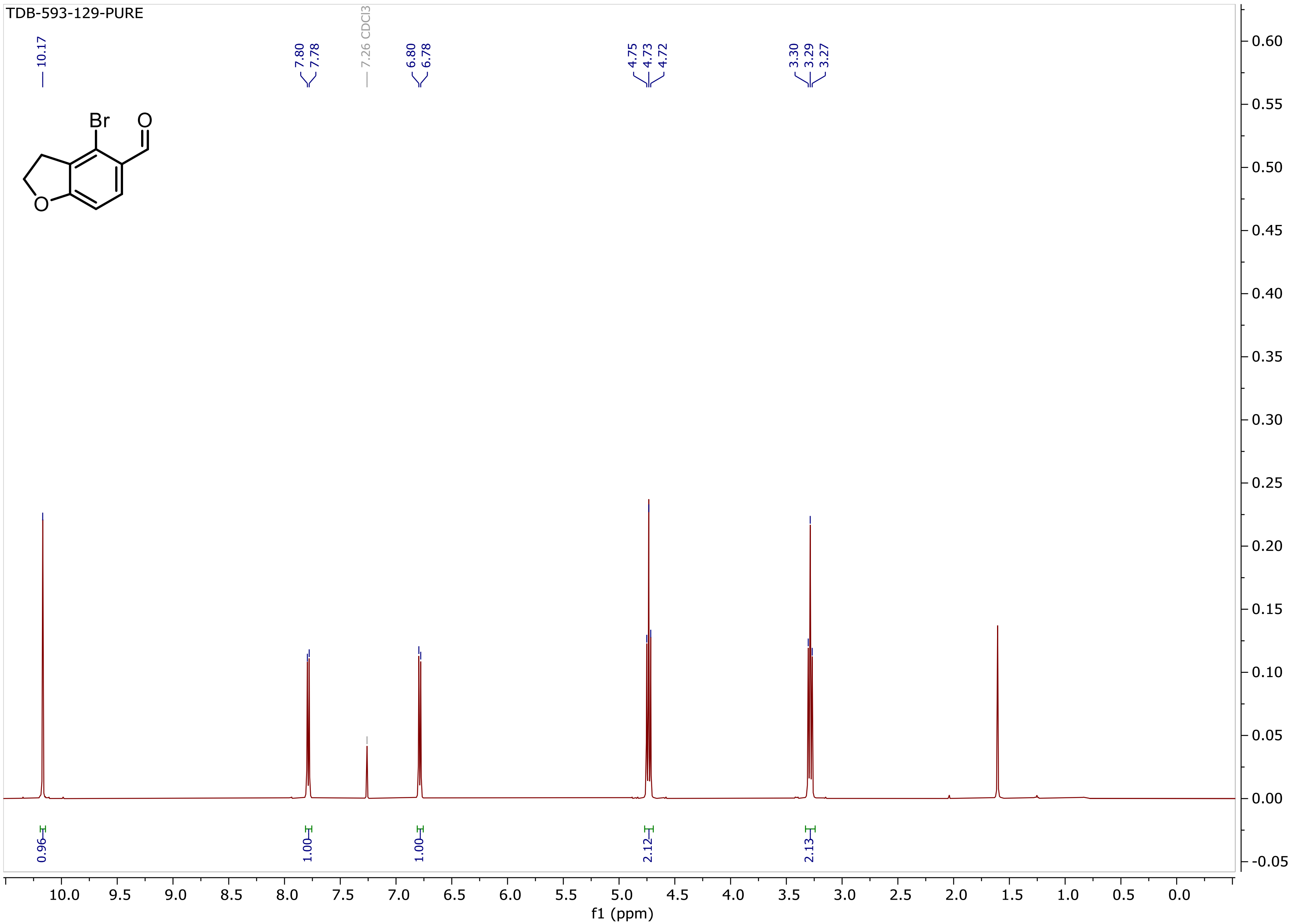

^{13}C NMR (126 MHz, $CDCl_3$) spectrum of 4-bromo-2,3-dihydrobenzofuran-5-carbaldehyde (**41**):

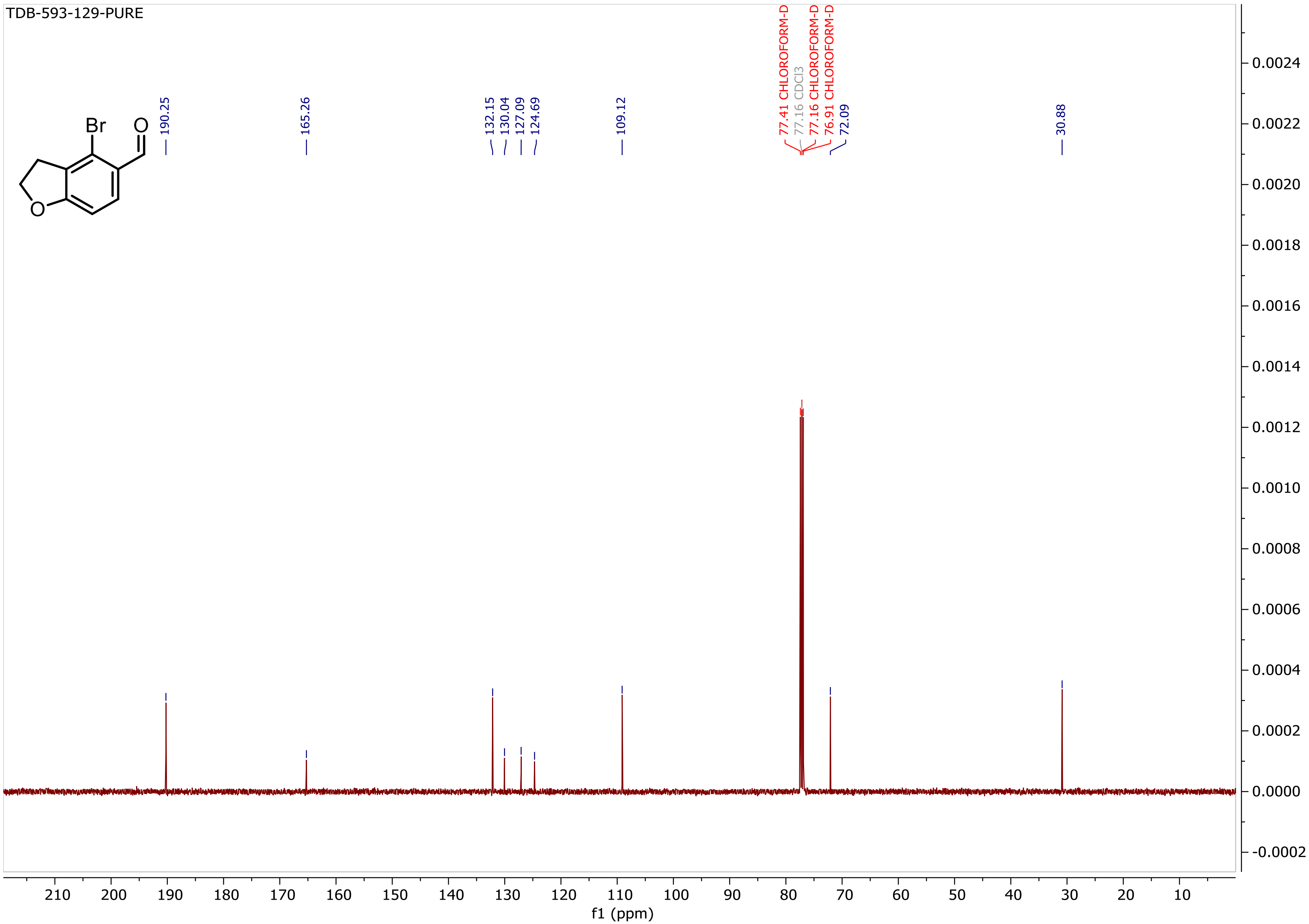

^{1}H NMR (500 MHz, CDCl$_3$) spectrum of (4-bromo-2,3-dihydrobenzofuran-5-yl)methanol:

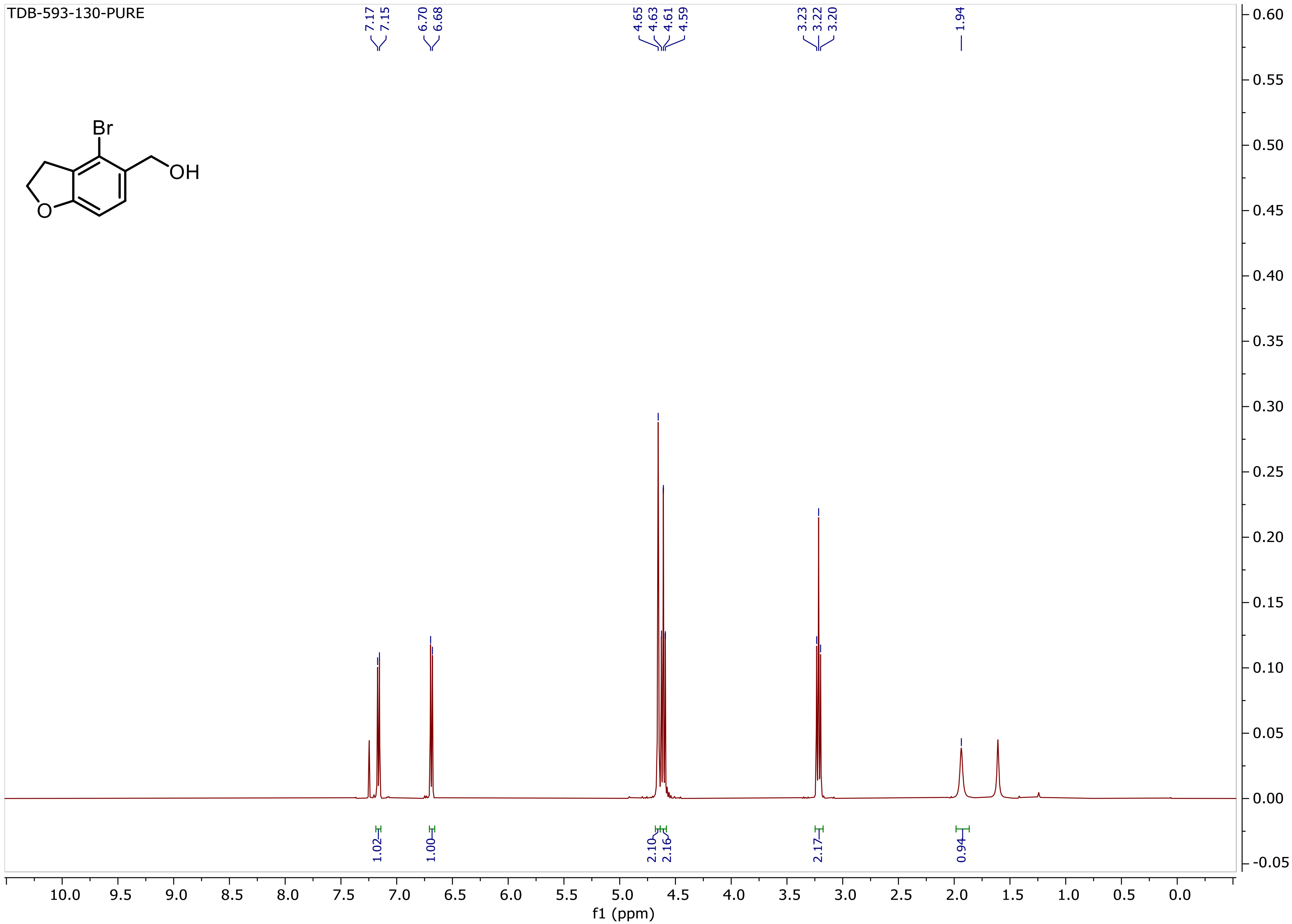

^{13}C NMR (126 MHz, CDCl$_3$) spectrum of (4-bromo-2,3-dihydrobenzofuran-5-yl)methanol:

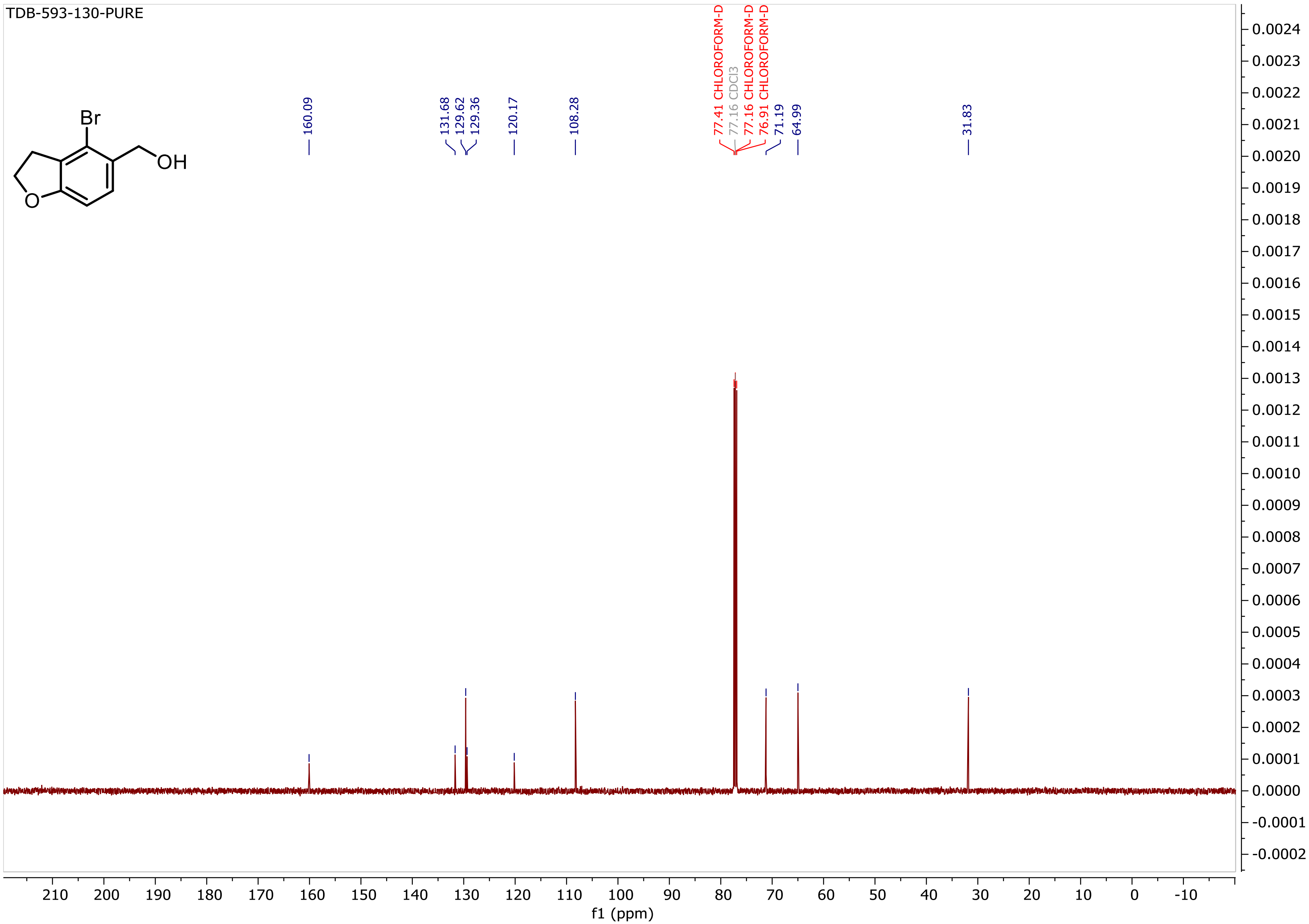

^{1}H NMR (500 MHz, $CDCl_3$) spectrum of *tert*-butyl ((4-bromo-2,3-dihydrobenzofuran-5-yl)methoxy)carbamate (**44**):

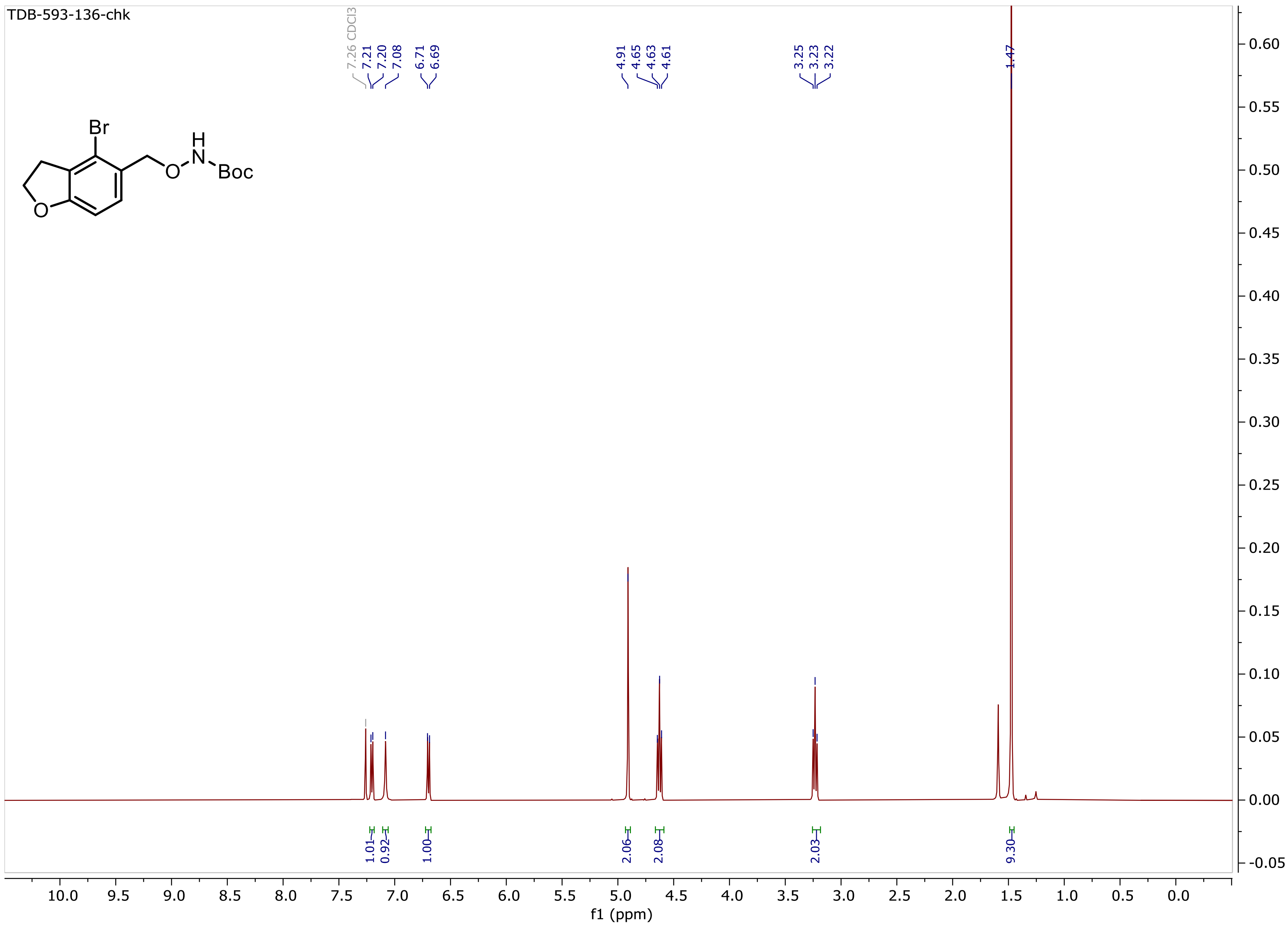

^{13}C NMR (126 MHz, CDCl$_3$) spectrum of *tert*-butyl ((4-bromo-2,3-dihydrobenzofuran-5-yl)methoxy)carbamate (**44**):

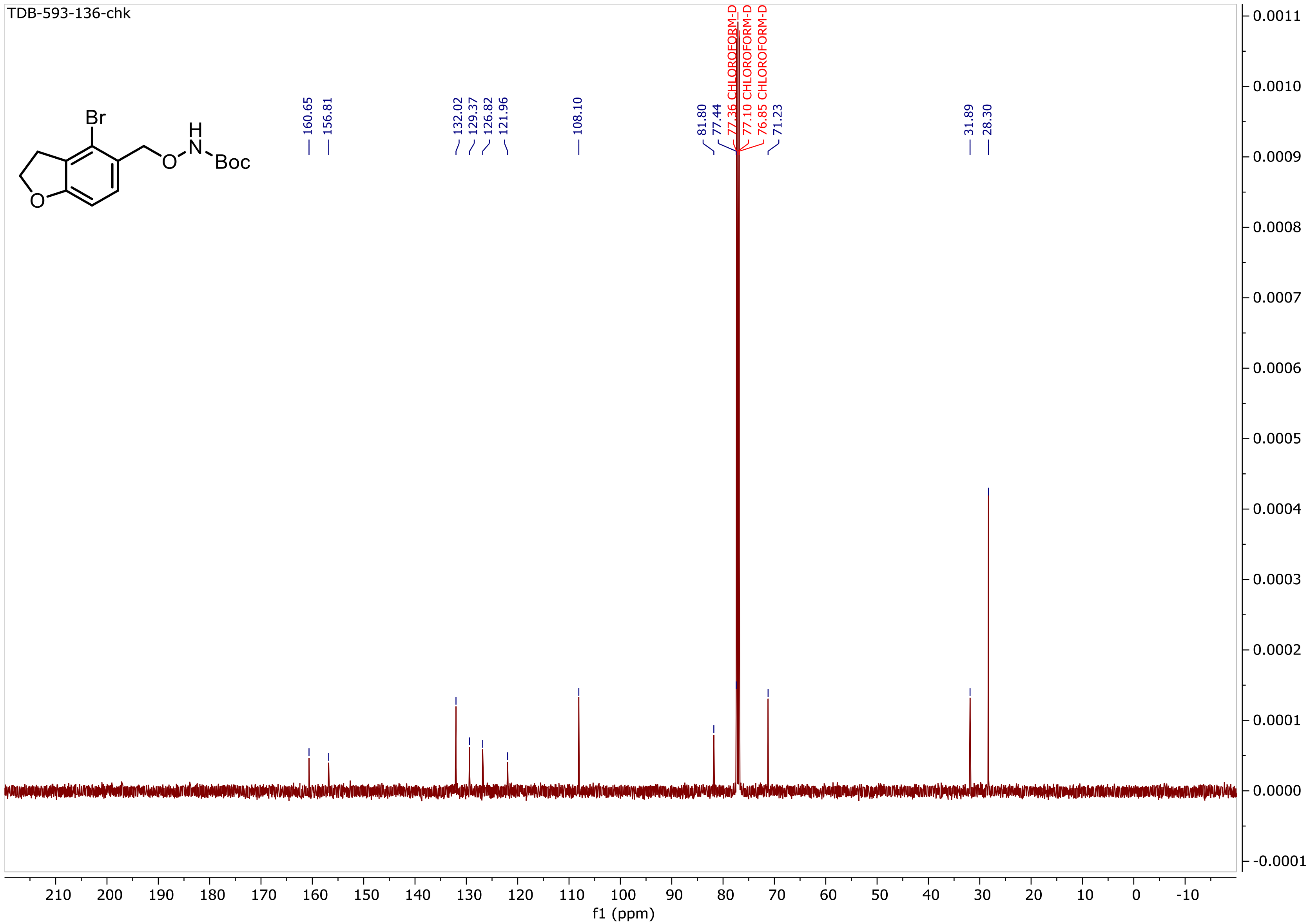

^{1}H NMR (500 MHz, CDCl$_3$) spectrum *tert*-butyl (2-(7,8-dihydrobenzofuro[4,5-*c*]isoxazol-1(3*H*)-yl)ethyl)carbamate (**46**):

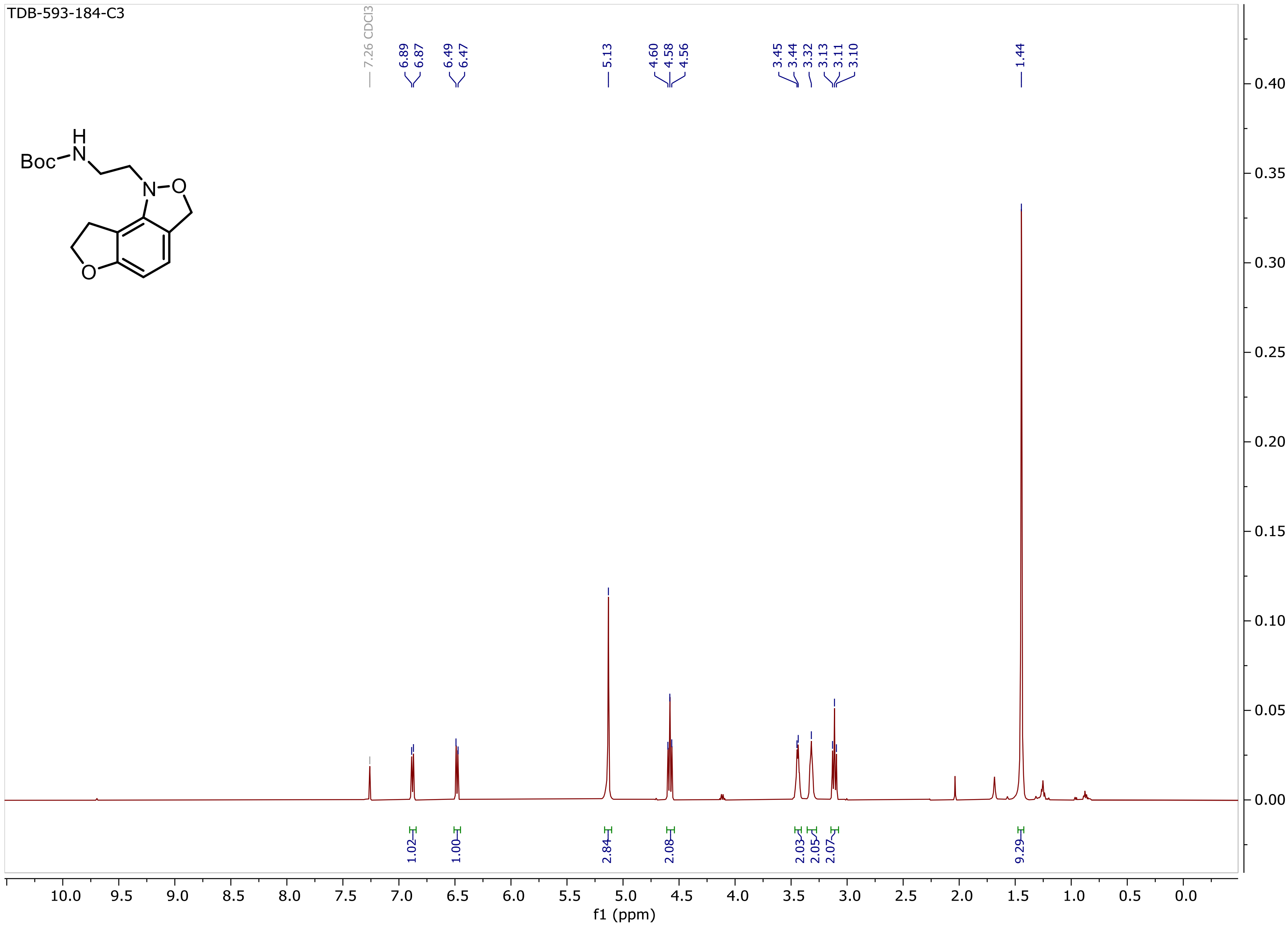

^{13}C NMR (126 MHz, CDCl$_3$) spectrum *tert*-butyl (2-(7,8-dihydrobenzofuro[4,5-c]isoxazol-1(*3H*)-yl)ethyl)carbamate (**46**):

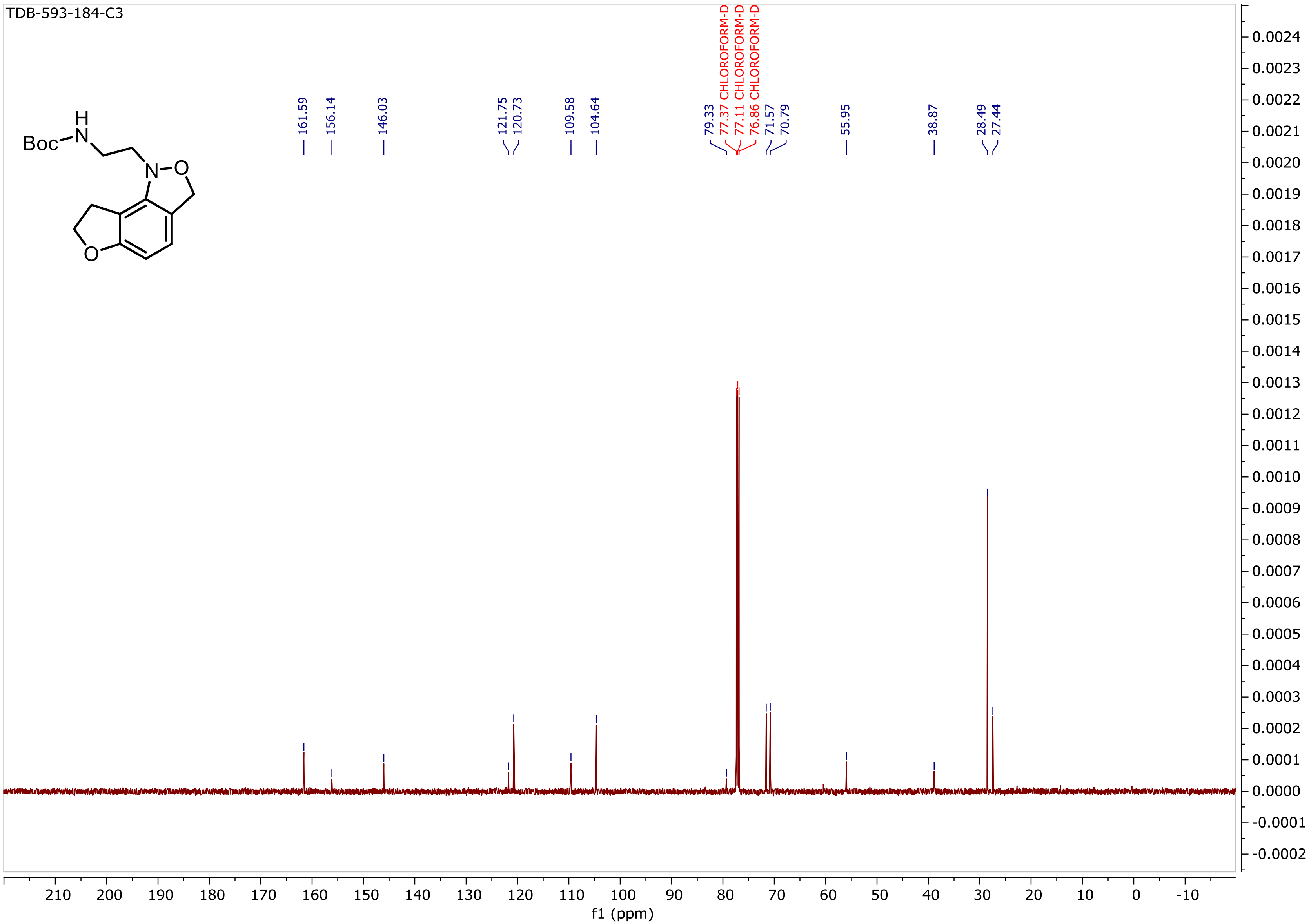

^{1}H NMR (500 MHz, CDCl$_3$) spectrum *N*-(2-(7,8-Dihydrobenzofuro[4,5-c]isoxazol-1(*3H*)-yl)ethyl)propionamide (**2**):

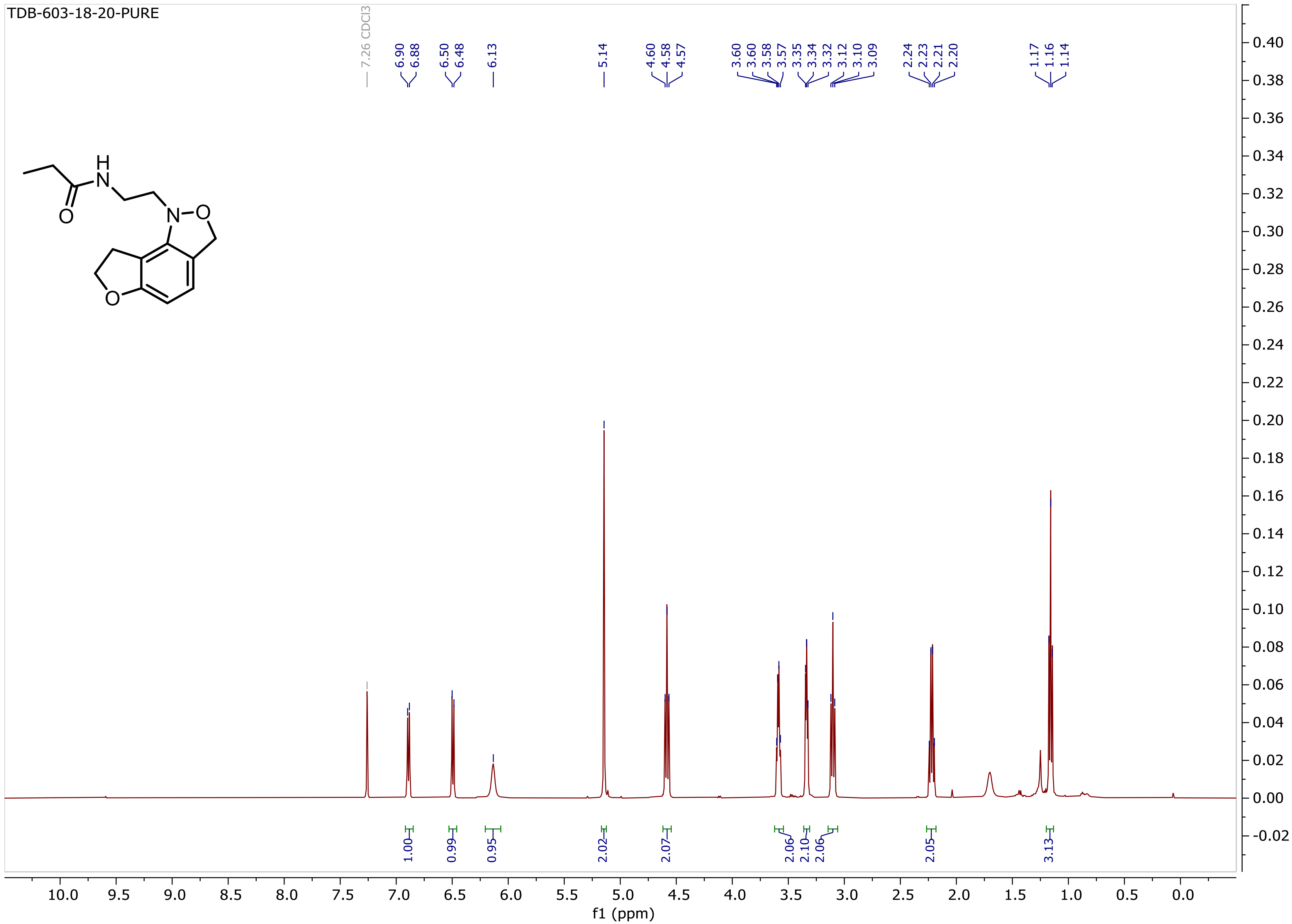

^{13}C NMR (126 MHz, CDCl$_3$) spectrum *N*-(2-(7,8-Dihydrobenzofuro[4,5-c]isoxazol-1(*3H*)-yl)ethyl)propionamide (**2**):

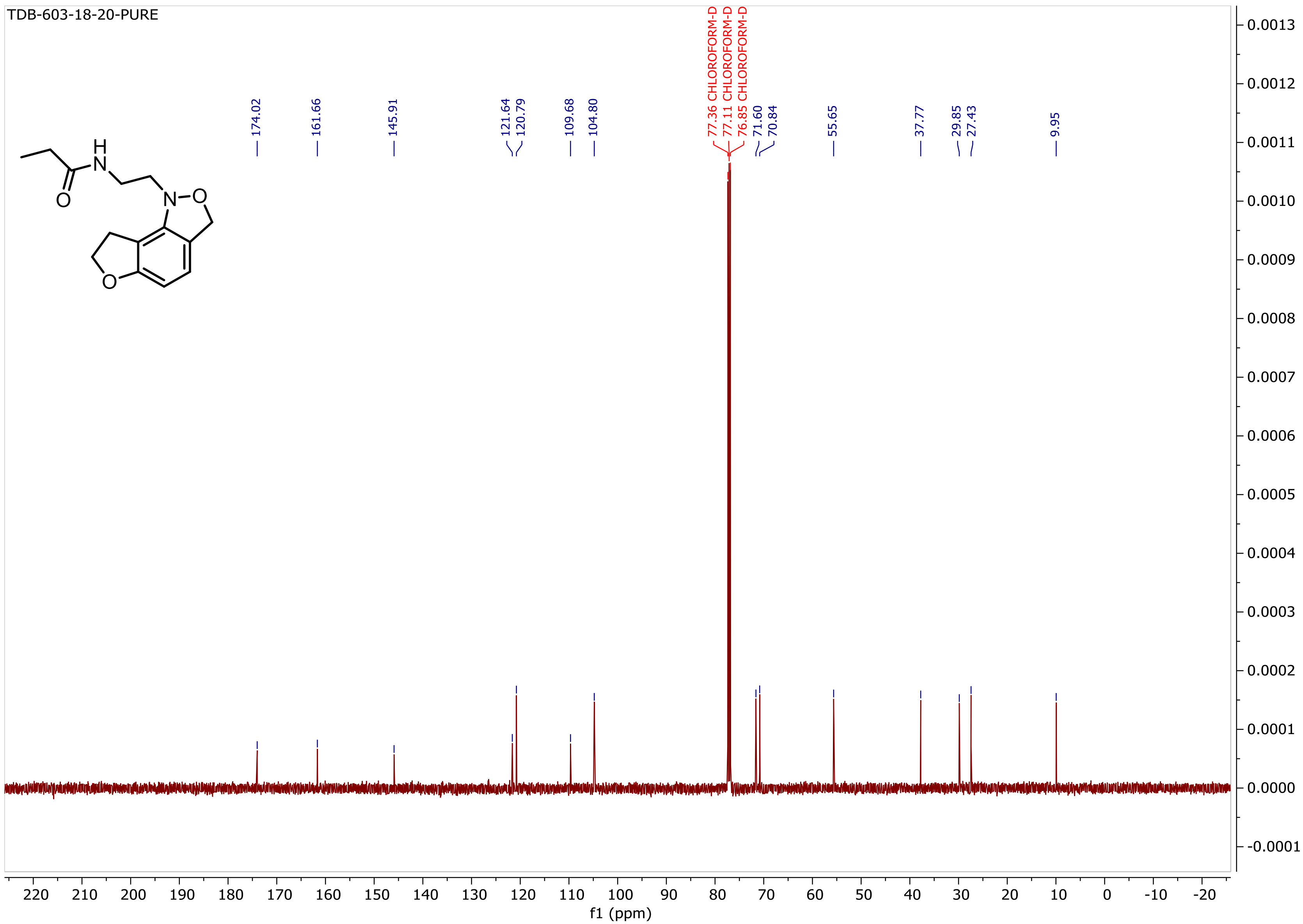

References

(1) Weinstock, L. T.; Cheng, C. C. A Structural Modification Study of Procarbazine. *J. Med. Chem.* **2002**, 22, 594–597. DOI: https://doi.org/10.1021/jm00191a029

(2) Boucher, M. M.; Furigay, M. H.; Quach, P. K.; Brindle, C. S. Liquid–Liquid Extraction Protocol for the Removal of Aldehydes and Highly Reactive Ketones from Mixtures. *Org. Process Res. Dev.* **2017**, 21, 1394–1403. DOI: https://doi.org/10.1021/acs.oprd.7b00231

(3) Bedford, R. B.; Haddow, M. F.; Mitchell, C. J.; Webster, R. L. Mild C–H Halogenation of Anilides and the Isolation of an Unusual Palladium(I)–Palladium(II) Species. *Angew. Chem. Int. Ed.* **2011**, 50, 5524–5527. DOI: https://doi.org/10.1002/anie.201101606

(4) Pharmaron. Laboratory Services, Physicochemical Assays. https://www.pharmaron.com/services/laboratory-services/dmpk-services/us-lab-services-dmpk-e-store/physicochemical/ (accessed 2026-05-06).

(5) Roth, B. L. Assay Protocol Book Version III; National Institute of Mental Health Psychoactive Drug Screening Program (NIMH PDSP): Chapel Hill, NC, 2018. https://pdsp.unc.edu/pdspweb/content/UNC-CH%20Protocol%20Book.pdf.

(6) Pracht, P.; Bohle, F.; Grimme, S. Automated Exploration of the Low-Energy Chemical Space with Fast Quantum Chemical Methods. *Phys. Chem. Chem. Phys.* **2020**, 22, 7169–7192. DOI: https://doi.org/10.1039/C9CP06869D

(7) Pracht, P.; Grimme, S.; Bannwarth, C.; et al. CREST—A Program for the Exploration of Low-Energy Molecular Chemical Space. *J. Chem. Phys.* **2024**, 160, 114110. DOI: https://doi.org/10.1063/5.0197592

(8) Spicher, S.; Grimme, S. Robust Atomistic Modeling of Materials, Organometallic, and Biochemical Systems. *Angew. Chem. Int. Ed.* **2020**, 59, 15665–15673. DOI: https://doi.org/10.1002/anie.202004239

(9) Anstine, D.; Zubatyuk, R.; Isayev, O. AIMNet2: A Neural Network Potential to Meet Your Neutral, Charged, Organic, and Elemental-Organic Needs. *ChemRxiv* **2024**. DOI: https://doi.org/10.26434/chemrxiv-2023-296ch-v3

(10) Lin, Y.S.; Li, G.D.; Mao, S.P.; Chai, J.D. Long-Range Corrected Hybrid Density Functionals with Improved Dispersion Corrections. *J. Chem. Theory Comput.* **2013**, 9, 263–272. DOI: https://doi.org/10.1021/ct300715s

(11) Rowan Scientific. https://www.rowansci.com (accessed 2026-05-06).

(12) Ehlert, S.; Stahn, M.; Spicher, S.; Grimme, S. Robust and Efficient Implicit Solvation Model for Fast Semiempirical Methods. *J. Chem. Theory Comput.* **2021**, 17, 4250–4261. DOI: https://doi.org/10.1021/acs.jctc.1c00471

(13) de Souza, B. GOAT: A Global Optimization Algorithm for Molecules and Atomic Clusters. *Angew. Chem. Int. Ed.* **2025**, 64, e202500393. DOI: https://doi.org/10.1002/anie.202500393

(14) Bannwarth, C.; Ehlert, S.; Grimme, S. GFN2-xTB—An Accurate and Broadly Parametrized Self-Consistent Tight-Binding Quantum Chemical Method with Multipole Electrostatics and Density-Dependent Dispersion Contributions. *J. Chem. Theory Comput.* **2019**, 15, 1652–1671. DOI: https://doi.org/10.1021/acs.jctc.8b01176

(15) Bannwarth, C.; Caldeweyher, E.; Ehlert, S.; Hansen, A.; Pracht, P.; Seibert, J.; Spicher, S.; Grimme, S. Extended Tight-Binding Quantum Chemistry Methods. *WIREs Comput. Mol. Sci.* **2021**, 11, e1493. DOI: https://doi.org/10.1002/wcms.1493

(16) Neese, F. Software Update: The ORCA Program System—Version 6.0. *WIREs Comput. Mol. Sci.* **2025**, 15, e70019. DOI: https://doi.org/10.1002/wcms.70019

(17) Neese, F. The ORCA Program System. *WIREs Comput. Mol. Sci.* **2012**, 2, 73–78. DOI: https://doi.org/10.1002/wcms.81

(18) Neese, F.; Wennmohs, F.; Hansen, A.; Becker, U. Efficient, Approximate and Parallel Hartree–Fock and Hybrid DFT Calculations. A 'Chain-of-Spheres' Algorithm for the Hartree–Fock Exchange. *Chem. Phys.* **2009**, 356, 98–109. DOI: https://doi.org/10.1016/j.chemphys.2008.10.036

(19) Grimme, S.; Antony, J.; Ehrlich, S.; Krieg, H. A Consistent and Accurate Ab Initio Parametrization of Density Functional Dispersion Correction (DFT-D) for the 94 Elements H–Pu. *J. Chem. Phys.* **2010**, 132, 154104. DOI: https://doi.org/10.1063/1.3382344

(20) Grimme, S.; Ehrlich, S.; Goerigk, L. Effect of the Damping Function in Dispersion Corrected Density Functional Theory. *J. Comput. Chem.* **2011**, 32, 1456–1465. DOI: https://doi.org/10.1002/jcc.21759

(21) Helmich-Paris, B.; de Souza, B.; Neese, F.; Izsák, R. An Improved Chain of Spheres for Exchange Algorithm. *J. Chem. Phys.* **2021**, 155, 104109. DOI: https://doi.org/10.1063/5.0058766

(22) Bykov, D.; Petrenko, T.; Izsák, R.; et al. Efficient Implementation of the Analytic Second Derivatives of Hartree–Fock and Hybrid DFT Energies: A Detailed Analysis of Different Approximations. *Mol. Phys.* **2015**, 113, 1961–1977. DOI: https://doi.org/10.1080/00268976.2015.1025114

(23) Garcia-Ratés, M.; Neese, F. Effect of the Solute Cavity on the Solvation Energy and Its Derivatives within the Framework of the Gaussian Charge Scheme. *J. Comput. Chem.* **2020**, 41, 922–939. DOI: https://doi.org/10.1002/jcc.26139

(24) Stylianakis, I.; Zervos, N.; Lii, J.-H.; Pantazis, D. A.; Kolocouris, A. Conformational Energies of Reference Organic Molecules: Benchmarking of Common Efficient Computational Methods against Coupled Cluster Theory. *J. Comput.-Aided Mol. Des.* **2023**, 37, 607–656. DOI: https://doi.org/10.1007/s10822-023-00513-5

(25) Cavasin, A. T.; Hillisch, A.; Uellendahl, F.; Schneckener, S.; Göller, A. H. Reliable and Performant Identification of Low-Energy Conformers in the Gas Phase and Water. *J. Chem. Inf. Model.* **2018**, 58, 1005–1020. DOI: https://doi.org/10.1021/acs.jcim.8b00151

(26) Fogueri, U. R.; Kozuch, S.; Karton, A.; Martin, J. M. L. The Melatonin Conformer Space: Benchmark and Assessment of Wave Function and DFT Methods for a Paradigmatic Biological and Pharmacological Molecule. *J. Phys. Chem. A* **2013**, 117, 2269–2277. DOI: https://doi.org/10.1021/jp312644t

(27) Stauch, B.; Johansson, L. C.; McCorvy, J. D.; et al. Structural Basis of Ligand Recognition at the Human MT1 Melatonin Receptor. *Nature* **2019**, 569, 284–288. DOI: https://doi.org/10.1038/s41586-019-1141-3

(28) Jorgensen, W. L.; Gao, J. Cis–Trans Energy Difference for the Peptide Bond in the Gas Phase and in Aqueous Solution. *J. Am. Chem. Soc.* **1988**, 110, 4212–4216. DOI: https://doi.org/10.1021/ja00221a020

(29) Sherman, W.; Day, T.; Jacobson, M. P.; Friesner, R. A.; Farid, R. Novel Procedure for Modeling Ligand/Receptor Induced Fit Effects. *J. Med. Chem.* **2006**, 49, 534–553. DOI: https://doi.org/10.1021/jm050540c

(30) Friesner, R. A.; Banks, J. L.; Murphy, R. B.; Halgren, T. A.; Klicic, J. J.; Mainz, D. T.; Repasky, M. P.; Knoll, E. H.; Shelley, M.; Perry, J. K. Glide: A New Approach for Rapid, Accurate Docking and Scoring. 1. Method and Assessment of Docking Accuracy. *J. Med. Chem.* **2004**, 47, 1739–1749. DOI: https://doi.org/10.1021/jm0306430

(31) Sherman, W.; Beard, H. S.; Farid, R. Use of an Induced Fit Receptor Structure in Virtual Screening. *Chem. Biol. Drug Des.* **2006**, 67, 83–84. DOI: https://doi.org/10.1111/j.1747-0285.2005.00327.x

(32) Jacobson, M. P.; Pincus, D. L.; Rapp, C. S.; et al. A Hierarchical Approach to All-Atom Protein Loop Prediction. *Proteins: Struct., Funct., Bioinf.* **2004**, 55, 351–367. DOI: https://doi.org/10.1002/prot.10613

(33) Schrödinger Release 2025-1; Schrödinger, LLC: New York, NY, 2025.

(34) Wallace, E. R. S.; Frey, N. C.; Rackers, J. A. Strain Problems Got You in a Twist? Try StrainRelief: A Quantum-Accurate Tool for Ligand Strain Calculations. *J. Chem. Inf. Model.* **2025**, 65, 6613–6620. DOI: https://doi.org/10.1021/acs.jcim.5c00586

.
